# Supplementary figures and images for: Saccharomyces cerevisiae DJ-1 paralogs maintain genome integrity through glycation repair of nucleic acids and proteins
Source: eLife. 2023 Aug 7;12:e88875. doi: 10.7554/eLife.88875 (PMC10431920; doi:10.7554/eLife.88875)

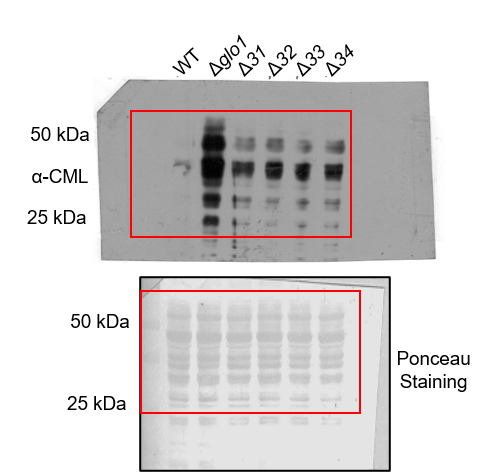

Supplement: Figure 1—source data 1. [file elife-88875-fig1-data1.zip › Figure 1B croped labelled.tif]

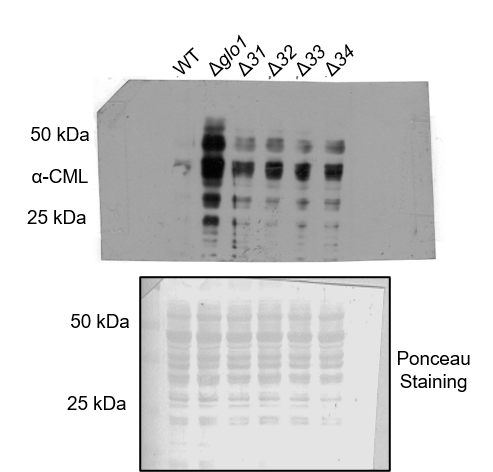

Supplement: Figure 1—source data 1. [file elife-88875-fig1-data1.zip › Figure 1B Original uncropped.tif]

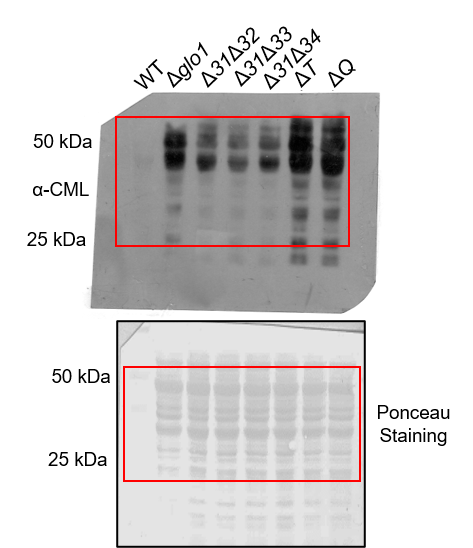

Supplement: Figure 1—source data 2. [file elife-88875-fig1-data2.zip › Figure 1C cropped labelled.tif]

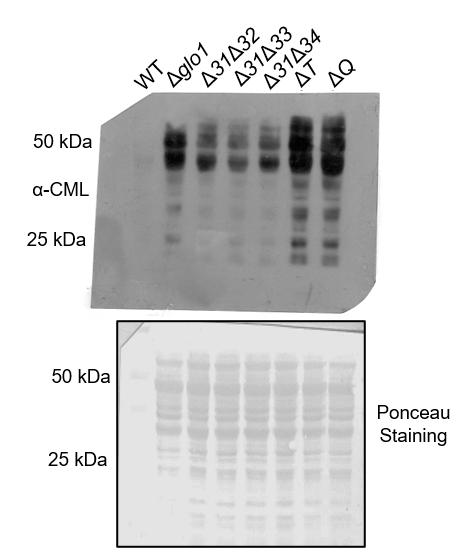

Supplement: Figure 1—source data 2. [file elife-88875-fig1-data2.zip › Figure 1C Original uncropped.tif]

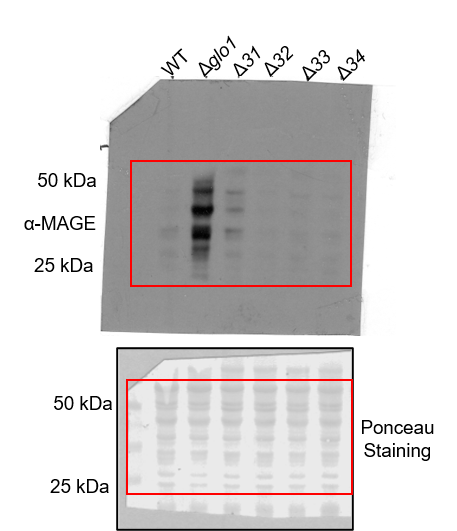

Supplement: Figure 1—source data 3. [file elife-88875-fig1-data3.zip › Figure 1D Cropped labelled.tif]

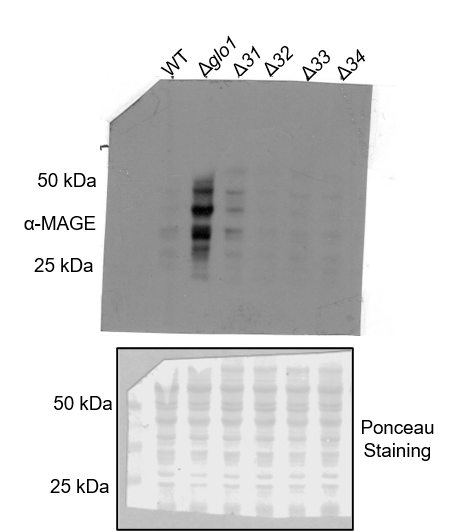

Supplement: Figure 1—source data 3. [file elife-88875-fig1-data3.zip › Figure 1D Orginal uncropped.tif]

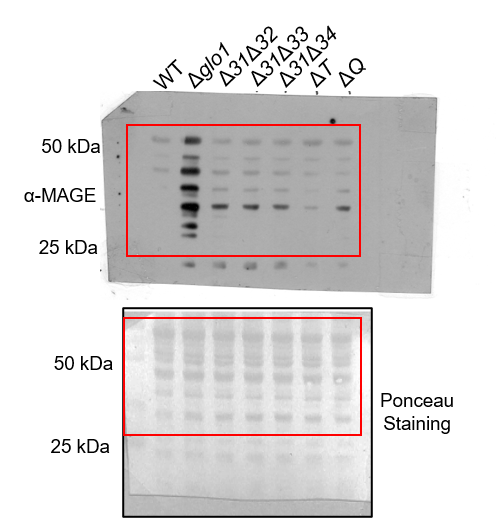

Supplement: Figure 1—source data 4. [file elife-88875-fig1-data4.zip › Figure 1E Cropped labelled.tif]

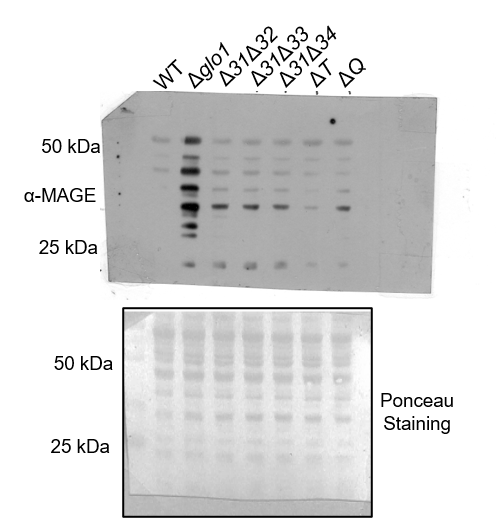

Supplement: Figure 1—source data 4. [file elife-88875-fig1-data4.zip › Figure 1E Original uncropped.tif]

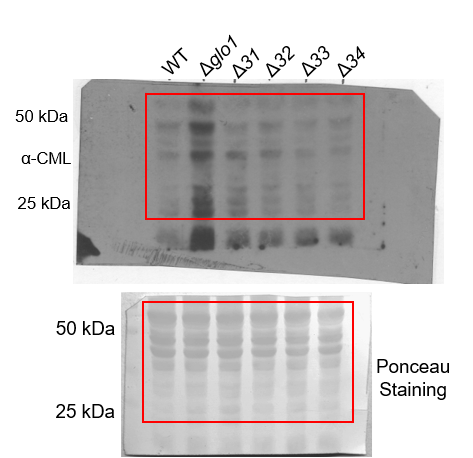

Supplement: Figure 1—figure supplement 1—source data 1. [file elife-88875-fig1-figsupp1-data1.zip › Figure S1B Cropped labelled.tif]

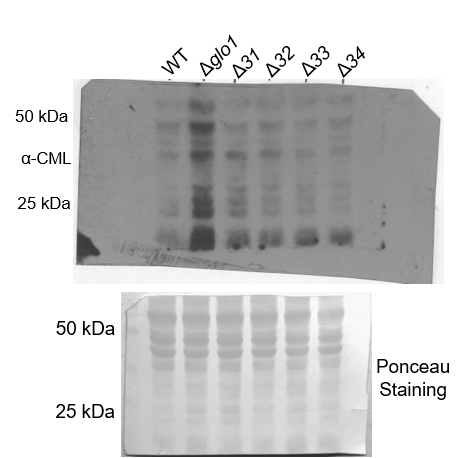

Supplement: Figure 1—figure supplement 1—source data 1. [file elife-88875-fig1-figsupp1-data1.zip › Figure S1B Original uncropped.tif]

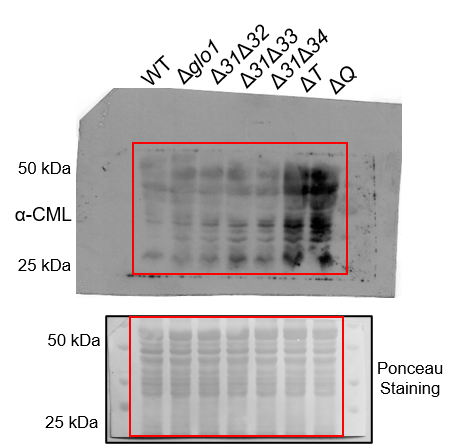

Supplement: Figure 1—figure supplement 1—source data 2. [file elife-88875-fig1-figsupp1-data2.zip › Figure S1C Cropped labelled.tif]

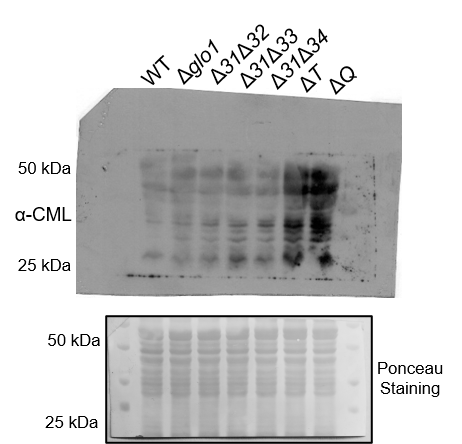

Supplement: Figure 1—figure supplement 1—source data 2. [file elife-88875-fig1-figsupp1-data2.zip › Figure S1C Original uncropped.tif]

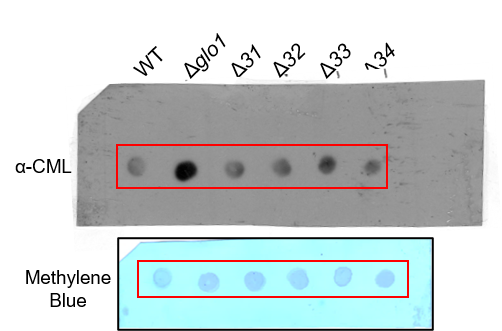

Supplement: Figure 2—source data 1. [file elife-88875-fig2-data1.zip › Figure 2A Cropped labelled.tif]

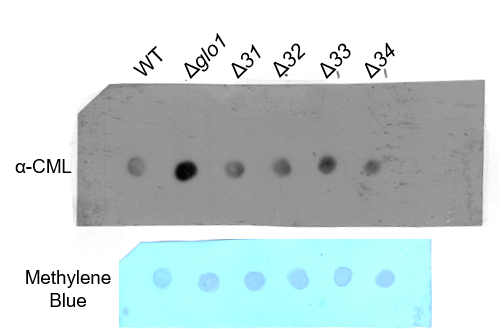

Supplement: Figure 2—source data 1. [file elife-88875-fig2-data1.zip › Figure 2A Original uncropped.tif]

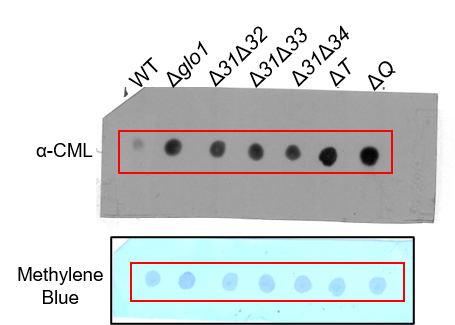

Supplement: Figure 2—source data 2. [file elife-88875-fig2-data2.zip › Figure 2B Cropped labelled.tif]

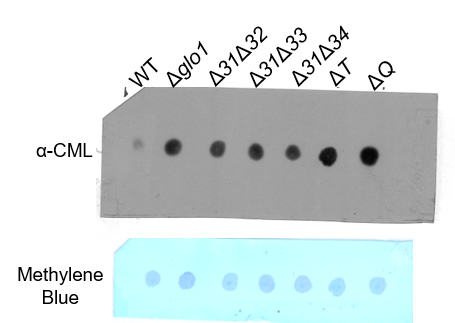

Supplement: Figure 2—source data 2. [file elife-88875-fig2-data2.zip › Figure 2B Original uncropped.tif]

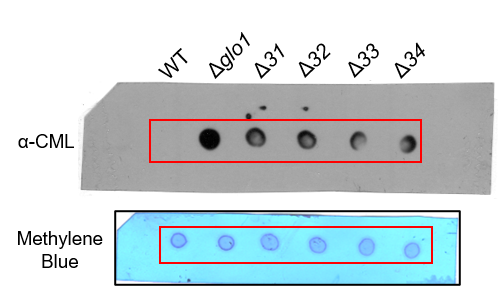

Supplement: Figure 2—source data 3. [file elife-88875-fig2-data3.zip › Figure 2C Cropped labelled.tif]

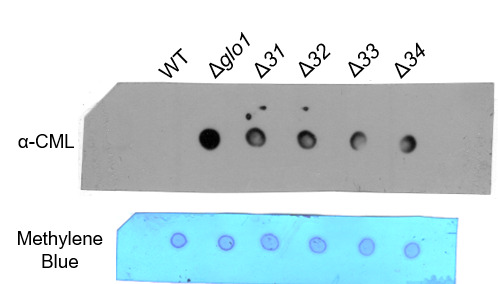

Supplement: Figure 2—source data 3. [file elife-88875-fig2-data3.zip › Figure 2C Original uncropped.tif]

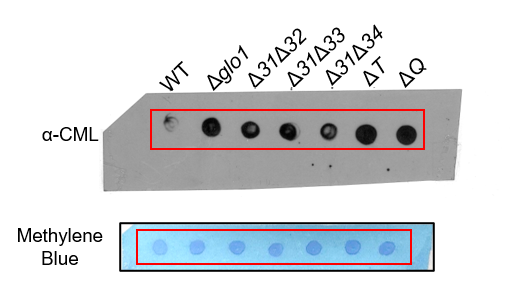

Supplement: Figure 2—source data 4. [file elife-88875-fig2-data4.zip › Figure 2D Cropped labelled.tif]

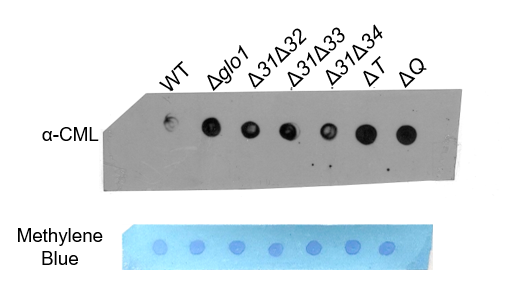

Supplement: Figure 2—source data 4. [file elife-88875-fig2-data4.zip › Figure 2D Original uncropped.tif]

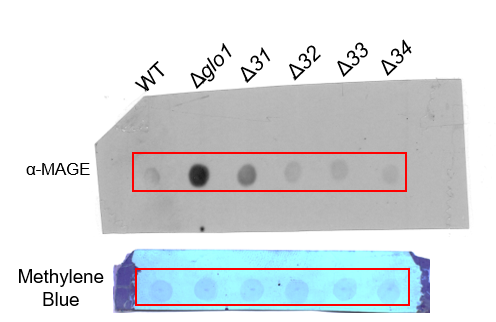

Supplement: Figure 2—source data 7. [file elife-88875-fig2-data7.zip › Figure 2G Cropped labelled.tif]

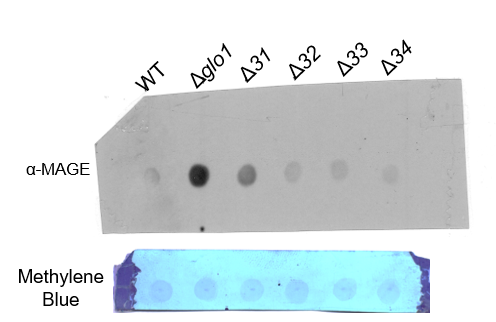

Supplement: Figure 2—source data 7. [file elife-88875-fig2-data7.zip › Figure 2G Original uncropped.tif]

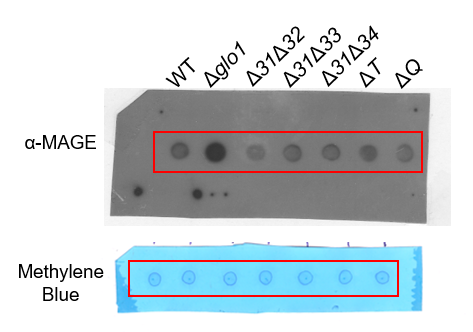

Supplement: Figure 2—source data 8. [file elife-88875-fig2-data8.zip › Figure 2H Cropped labelled.tif]

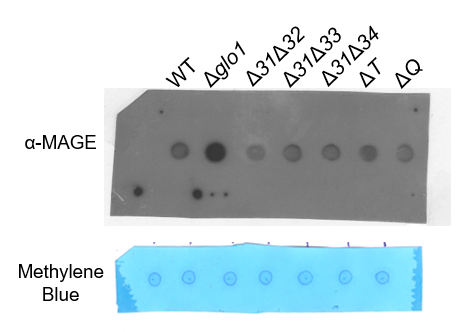

Supplement: Figure 2—source data 8. [file elife-88875-fig2-data8.zip › Figure 2H Original uncropped.tif]

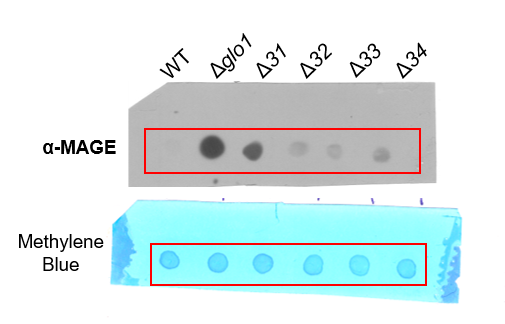

Supplement: Figure 2—source data 9. [file elife-88875-fig2-data9.zip › Figure 2I Cropped labelled.tif]

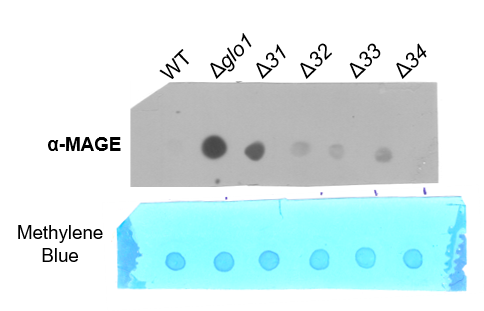

Supplement: Figure 2—source data 9. [file elife-88875-fig2-data9.zip › Figure 2I Original uncropped.tif]

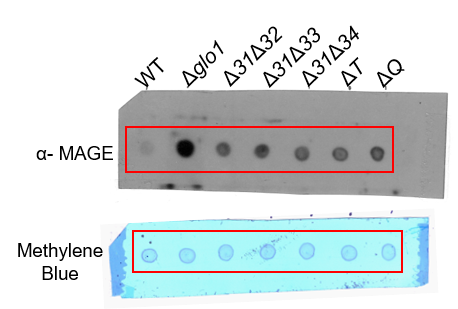

Supplement: Figure 2—source data 10. [file elife-88875-fig2-data10.zip › Figure 2J Cropped labeled.tif]

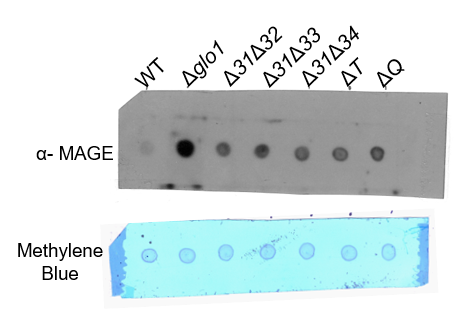

Supplement: Figure 2—source data 10. [file elife-88875-fig2-data10.zip › Figure 2J Original uncropped.tif]

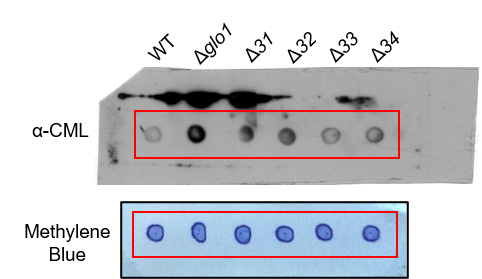

Supplement: Figure 2—figure supplement 1—source data 1. [file elife-88875-fig2-figsupp1-data1.zip › Figure S2A Cropped labelled.tif]

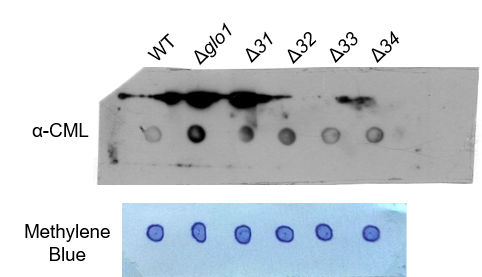

Supplement: Figure 2—figure supplement 1—source data 1. [file elife-88875-fig2-figsupp1-data1.zip › Figure S2A Original uncropped.tif]

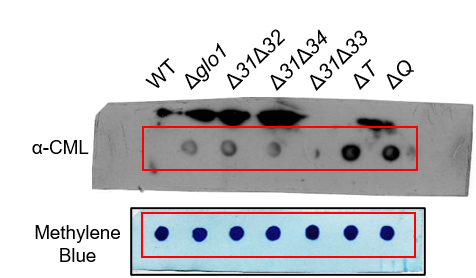

Supplement: Figure 2—figure supplement 1—source data 2. [file elife-88875-fig2-figsupp1-data2.zip › Figure S2B Cropped labelled.tif]

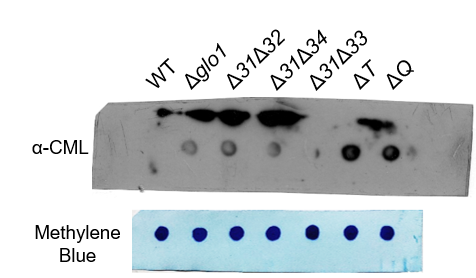

Supplement: Figure 2—figure supplement 1—source data 2. [file elife-88875-fig2-figsupp1-data2.zip › Figure S2B Original uncropped.tif]

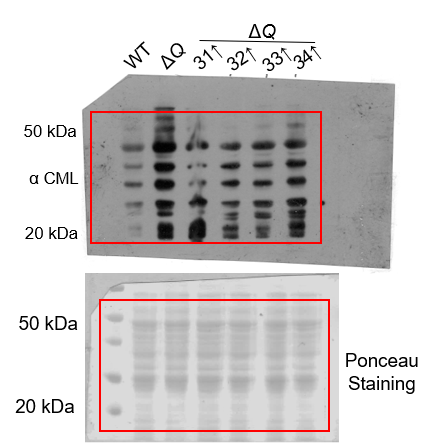

Supplement: Figure 3—source data 3. [file elife-88875-fig3-data3.zip › Figure 3D Cropped labelled.tif]

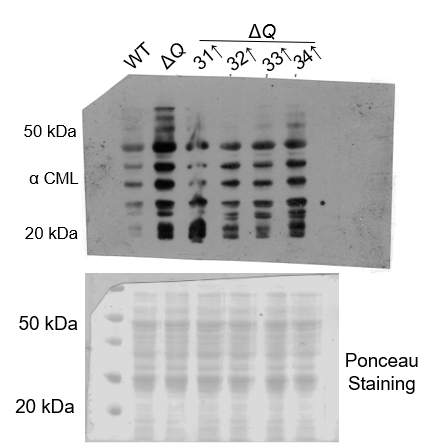

Supplement: Figure 3—source data 3. [file elife-88875-fig3-data3.zip › Figure 3D Original uncropped.tif]

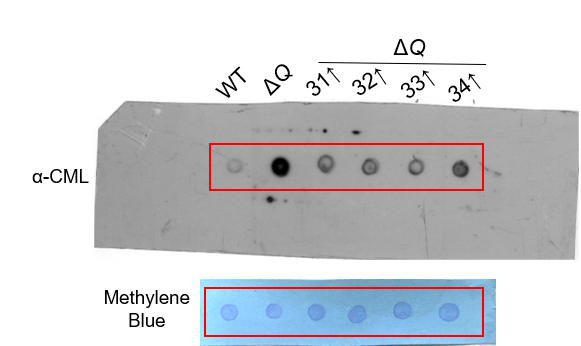

Supplement: Figure 3—source data 4. [file elife-88875-fig3-data4.zip › Figure 3E Cropped labelled.tif]

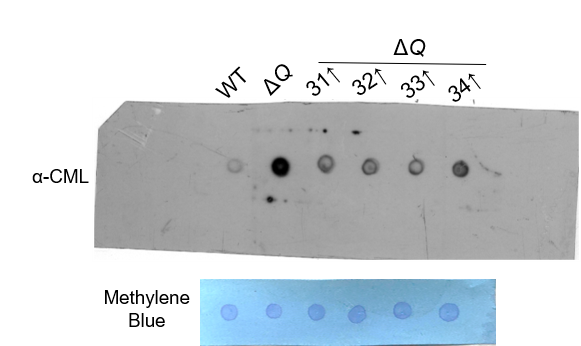

Supplement: Figure 3—source data 4. [file elife-88875-fig3-data4.zip › Figure 3E Original uncropped.tif]

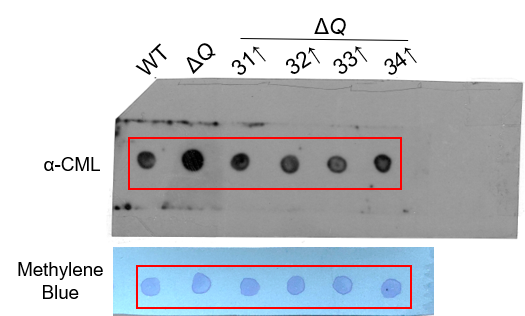

Supplement: Figure 3—source data 5. [file elife-88875-fig3-data5.zip › Figure 3F Cropped labelled.tif]

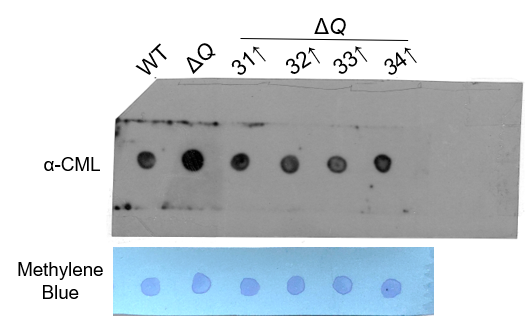

Supplement: Figure 3—source data 5. [file elife-88875-fig3-data5.zip › Figure 3F Original uncropped.tif]

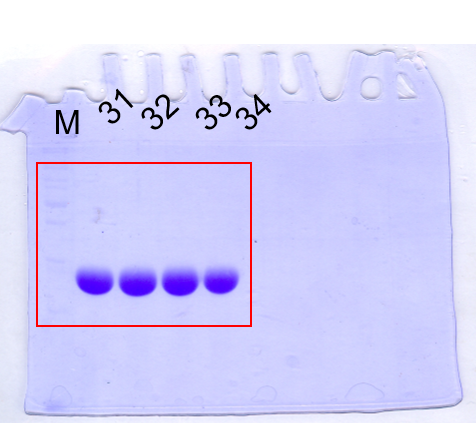

Supplement: Figure 3—figure supplement 1—source data 1. [file elife-88875-fig3-figsupp1-data1.zip › Figure S3A cropped labelled.tif]

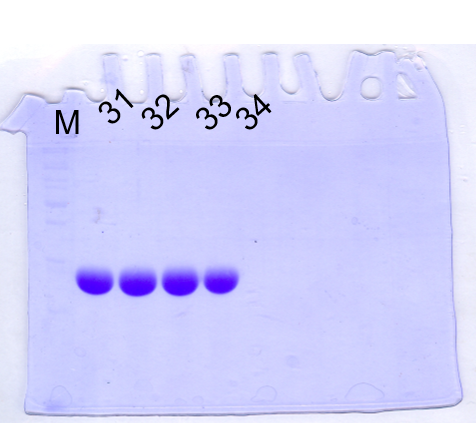

Supplement: Figure 3—figure supplement 1—source data 1. [file elife-88875-fig3-figsupp1-data1.zip › figure S3A uncropped original.tif]

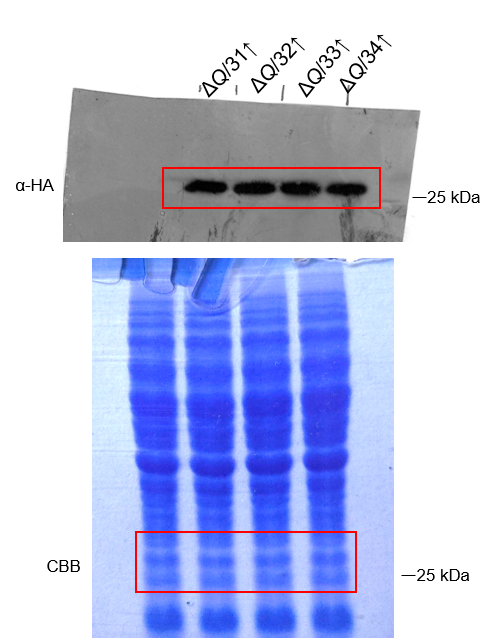

Supplement: Figure 3—figure supplement 1—source data 2. [file elife-88875-fig3-figsupp1-data2.zip › Figure S3C Cropped labelled.tif]

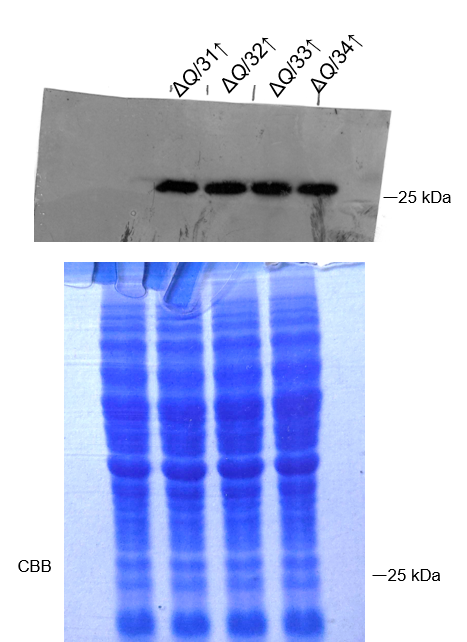

Supplement: Figure 3—figure supplement 1—source data 2. [file elife-88875-fig3-figsupp1-data2.zip › Figure S3C Original Uncropped.tif]

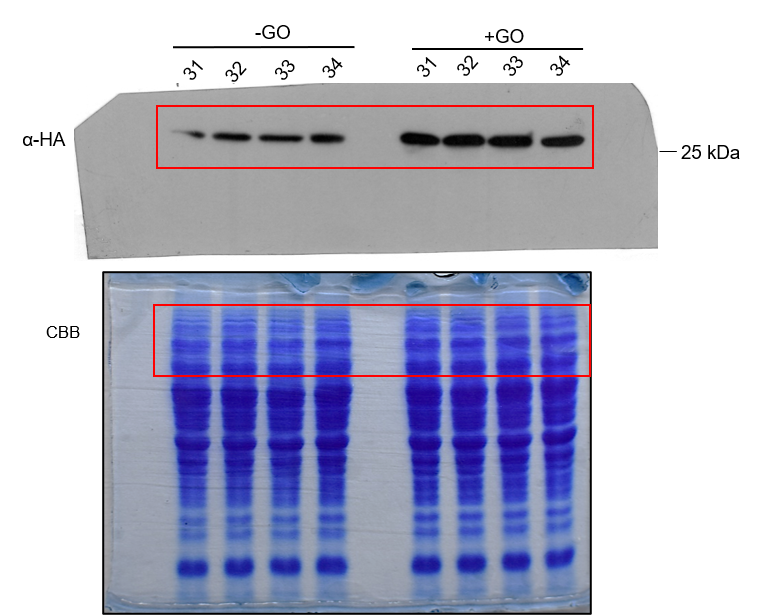

Supplement: Figure 3—figure supplement 1—source data 3. [file elife-88875-fig3-figsupp1-data3.zip › Figure S3D Cropped labelled.tif]

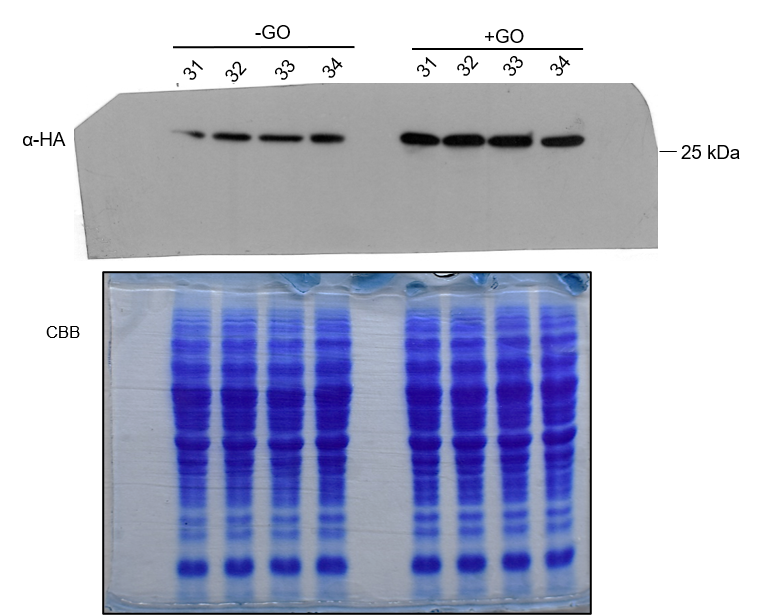

Supplement: Figure 3—figure supplement 1—source data 3. [file elife-88875-fig3-figsupp1-data3.zip › Figure S3D Original Uncropped.tif]

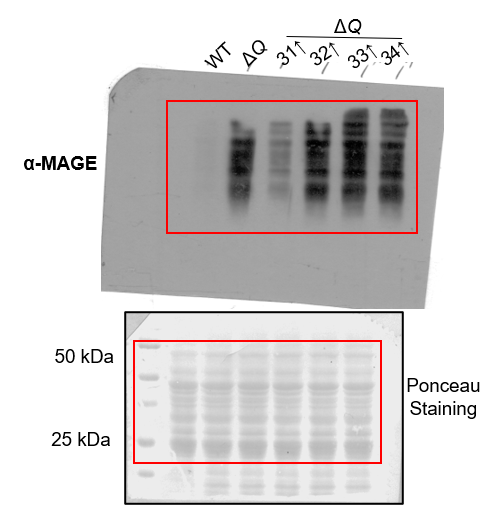

Supplement: Figure 4—source data 2. [file elife-88875-fig4-data2.zip › Figure 4C Cropped labelled.tif]

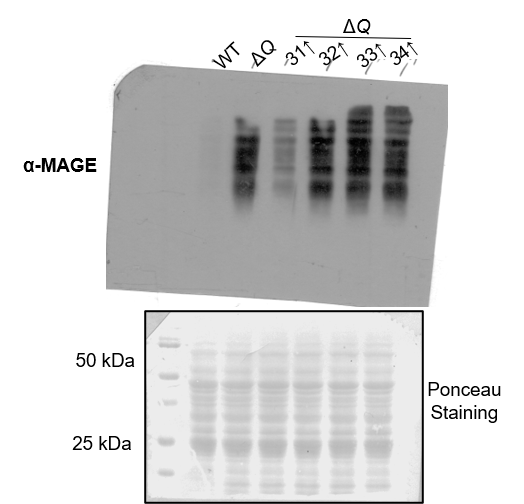

Supplement: Figure 4—source data 2. [file elife-88875-fig4-data2.zip › Figure 4C Original uncropped.tif]

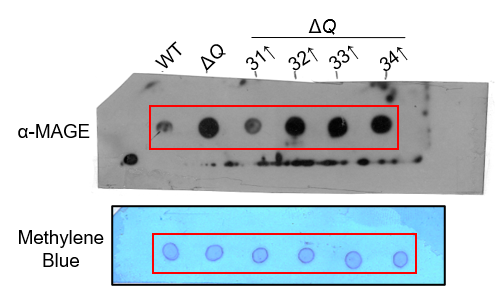

Supplement: Figure 4—source data 3. [file elife-88875-fig4-data3.zip › Figure 4D Cropped labelled.tif]

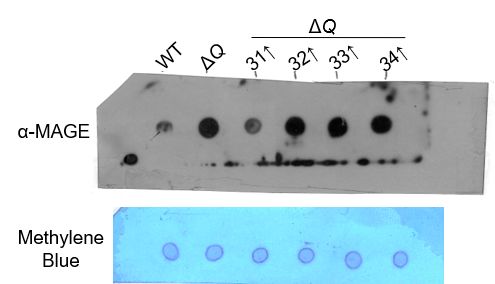

Supplement: Figure 4—source data 3. [file elife-88875-fig4-data3.zip › Figure 4D Original uncropped.tif]

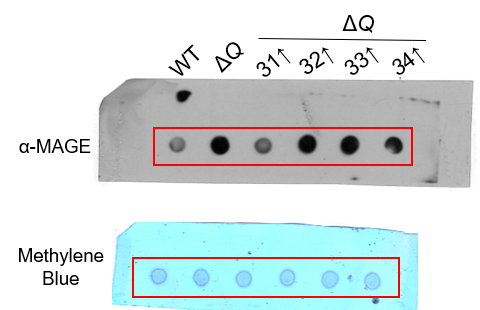

Supplement: Figure 4—source data 4. [file elife-88875-fig4-data4.zip › Figure 4E Cropped labelled.tif]

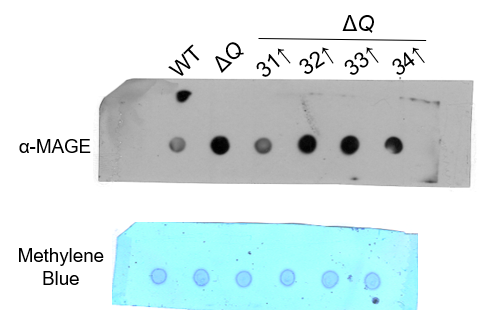

Supplement: Figure 4—source data 4. [file elife-88875-fig4-data4.zip › Figure 4E Original uncropped.tif]

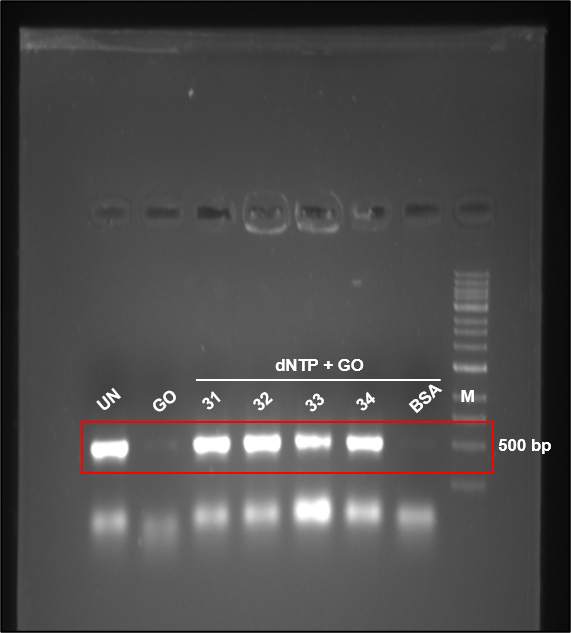

Supplement: Figure 5—source data 1. [file elife-88875-fig5-data1.zip › Figure 5B Cropped labelled.tif]

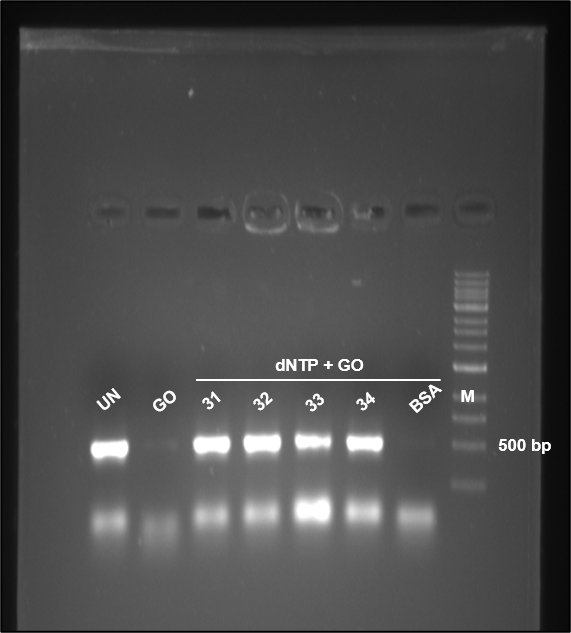

Supplement: Figure 5—source data 1. [file elife-88875-fig5-data1.zip › Figure 5B Original uncropped.tif]

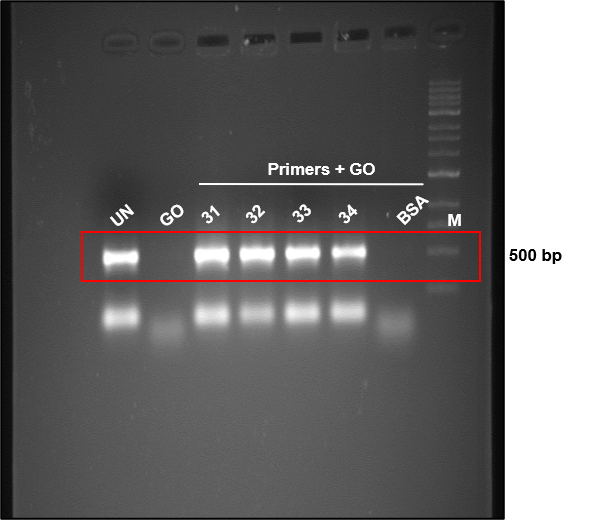

Supplement: Figure 5—source data 2. [file elife-88875-fig5-data2.zip › Figure 5C Cropped labelled.tif]

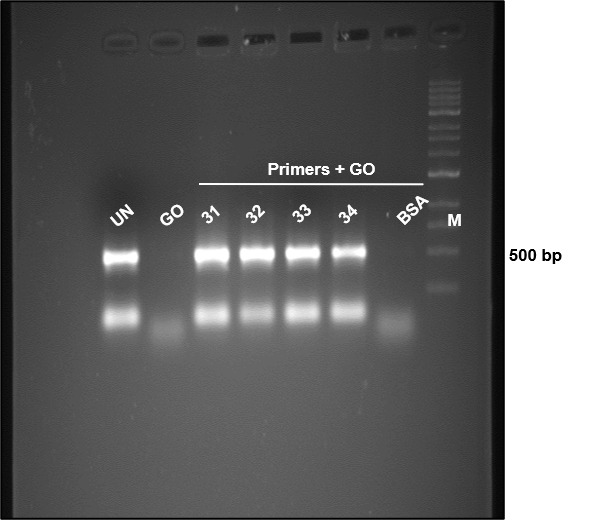

Supplement: Figure 5—source data 2. [file elife-88875-fig5-data2.zip › Figure 5C Original uncropped.tif]

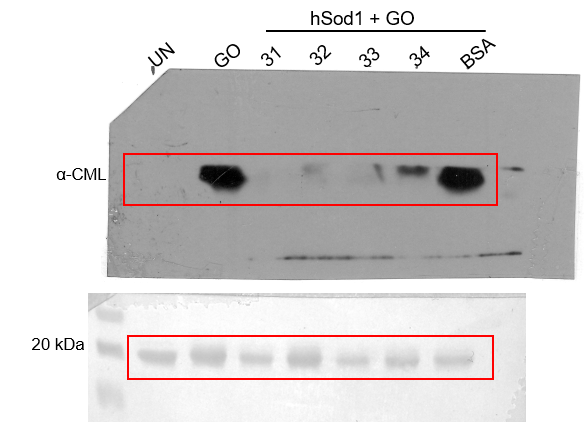

Supplement: Figure 5—source data 3. [file elife-88875-fig5-data3.zip › Figure 5D Cropped labelled.tif]

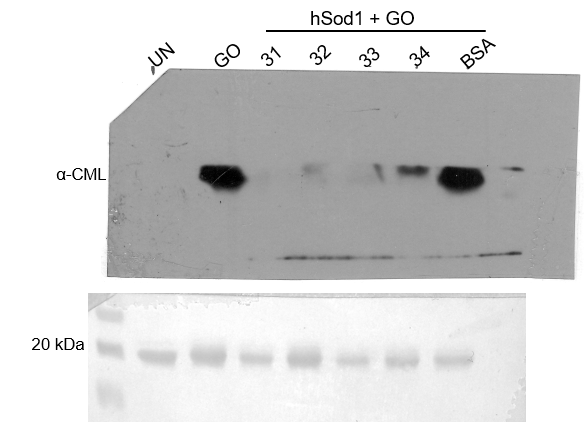

Supplement: Figure 5—source data 3. [file elife-88875-fig5-data3.zip › Figure 5D Original uncropped.tif]

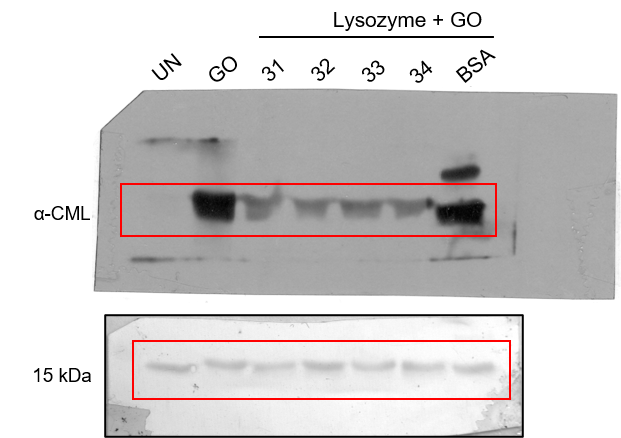

Supplement: Figure 5—source data 4. [file elife-88875-fig5-data4.zip › Figure 5E Cropped labelled.tif]

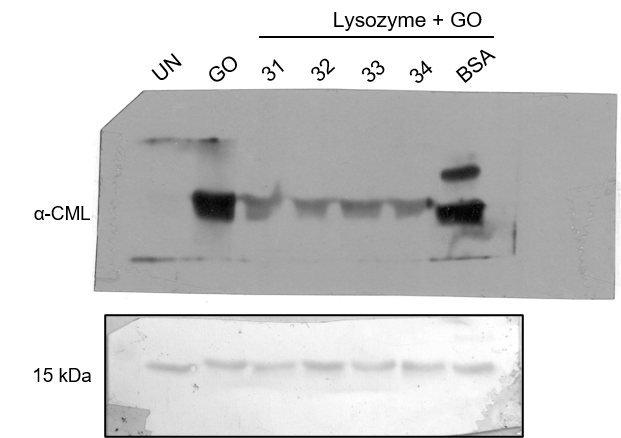

Supplement: Figure 5—source data 4. [file elife-88875-fig5-data4.zip › Figure 5E Original uncropped.tif]

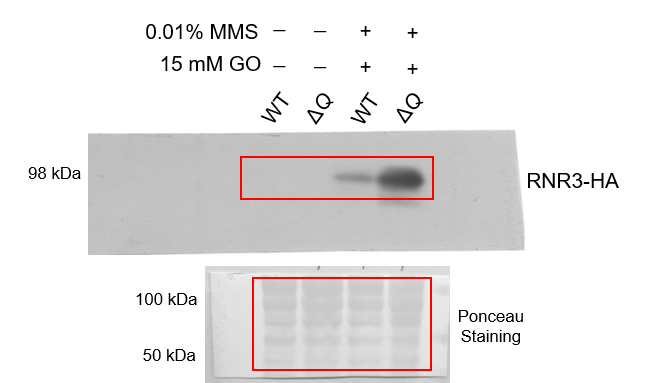

Supplement: Figure 5—source data 6. [file elife-88875-fig5-data6.zip › Figure 5H Cropped labelled.tif]

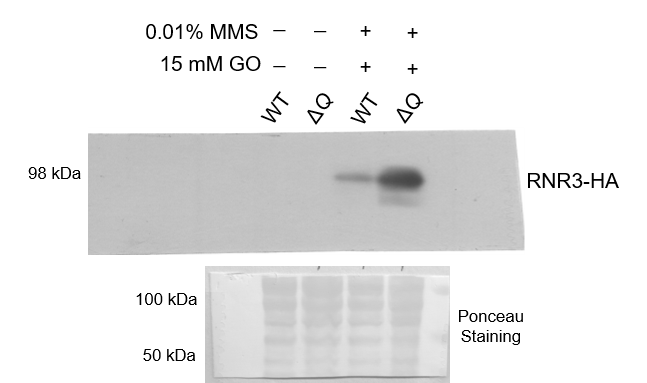

Supplement: Figure 5—source data 6. [file elife-88875-fig5-data6.zip › Figure 5H Original uncropped.tif]

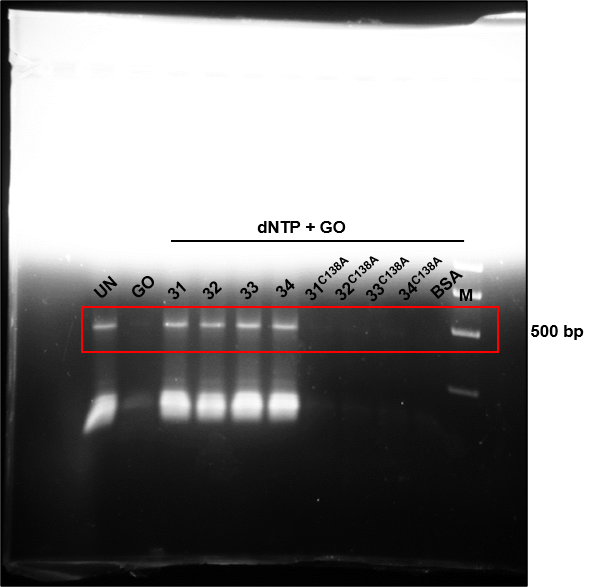

Supplement: Figure 5—figure supplement 1—source data 1. [file elife-88875-fig5-figsupp1-data1.zip › Figure S5A Cropped labelled.tif]

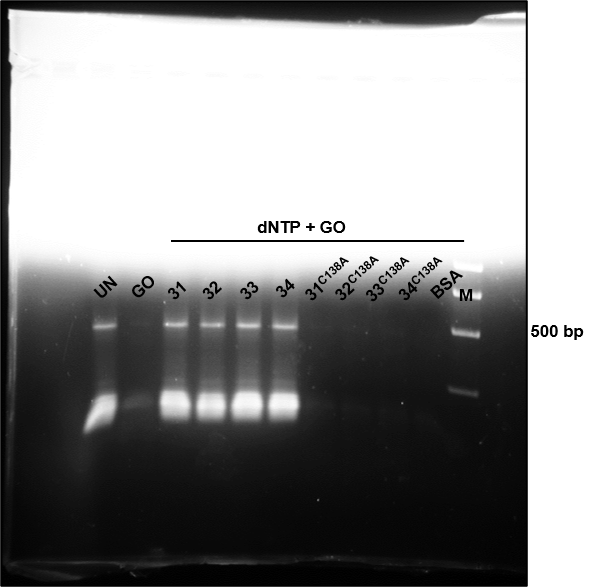

Supplement: Figure 5—figure supplement 1—source data 1. [file elife-88875-fig5-figsupp1-data1.zip › Figure S5A Original uncropped.tif]

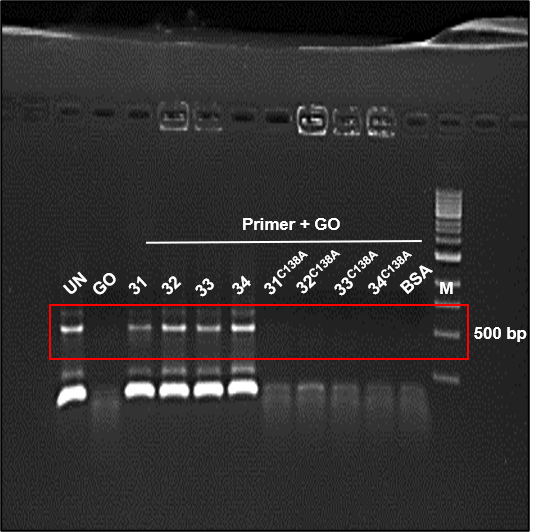

Supplement: Figure 5—figure supplement 1—source data 2. [file elife-88875-fig5-figsupp1-data2.zip › Figure S5B Cropped labelled.tif]

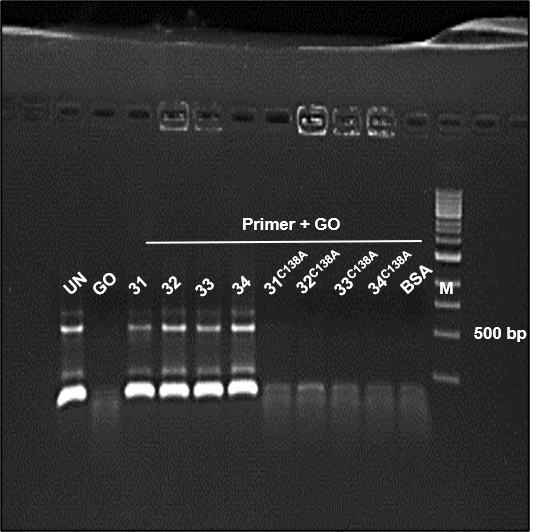

Supplement: Figure 5—figure supplement 1—source data 2. [file elife-88875-fig5-figsupp1-data2.zip › Figure S5B Original uncropped.tif]

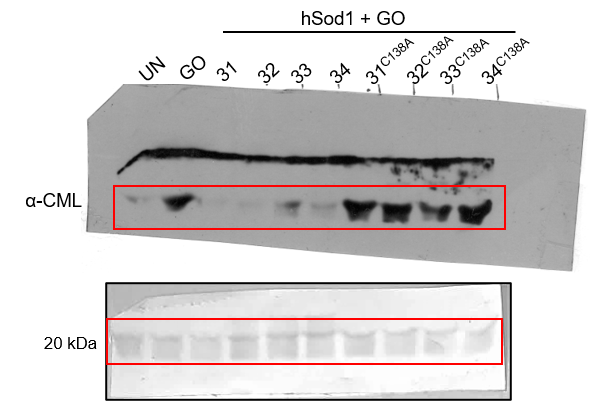

Supplement: Figure 5—figure supplement 1—source data 3. [file elife-88875-fig5-figsupp1-data3.zip › Figure S5C Cropped labelled.tif]

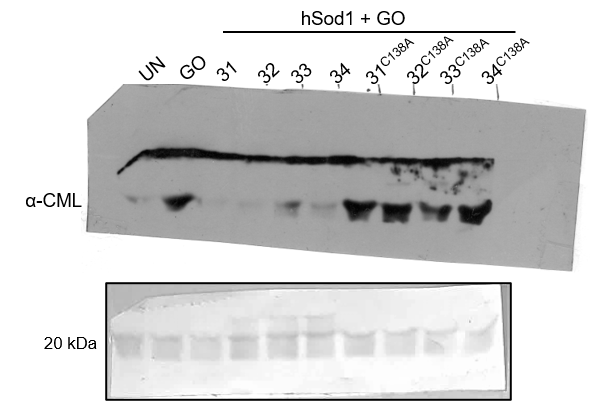

Supplement: Figure 5—figure supplement 1—source data 3. [file elife-88875-fig5-figsupp1-data3.zip › Figure S5C Original uncropped.tif]

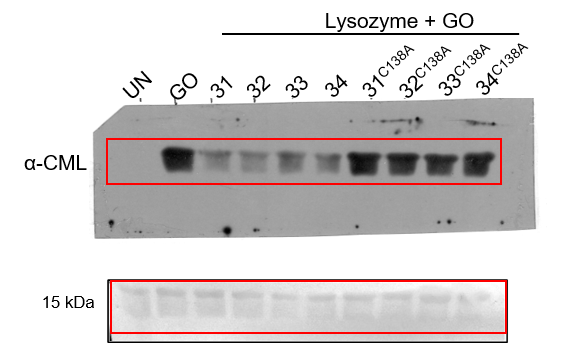

Supplement: Figure 5—figure supplement 1—source data 4. [file elife-88875-fig5-figsupp1-data4.zip › Figure S5D Cropped labelled.tif]

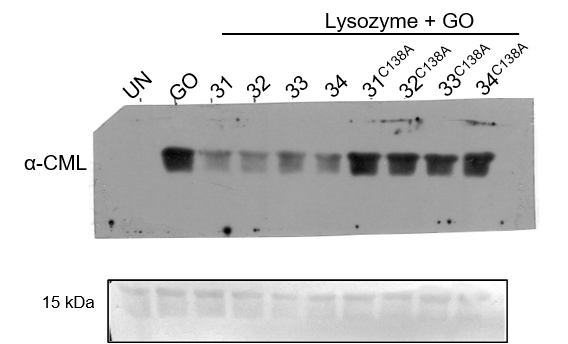

Supplement: Figure 5—figure supplement 1—source data 4. [file elife-88875-fig5-figsupp1-data4.zip › Figure S5D Original uncropped.tif]

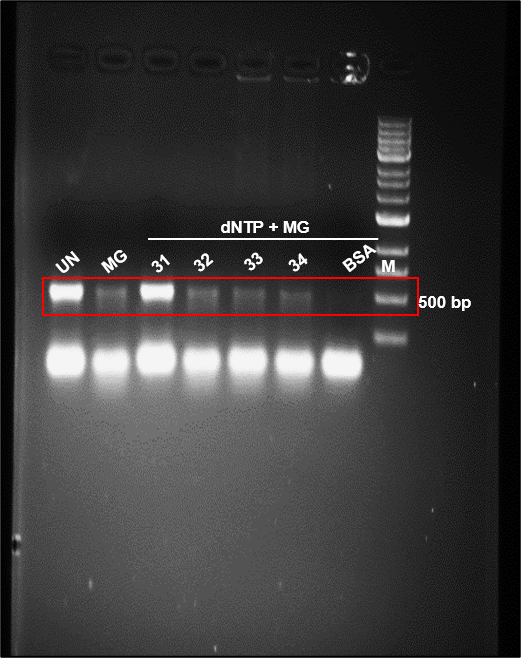

Supplement: Figure 6—source data 1. [file elife-88875-fig6-data1.zip › Figure 6B Cropped labelled.tif]

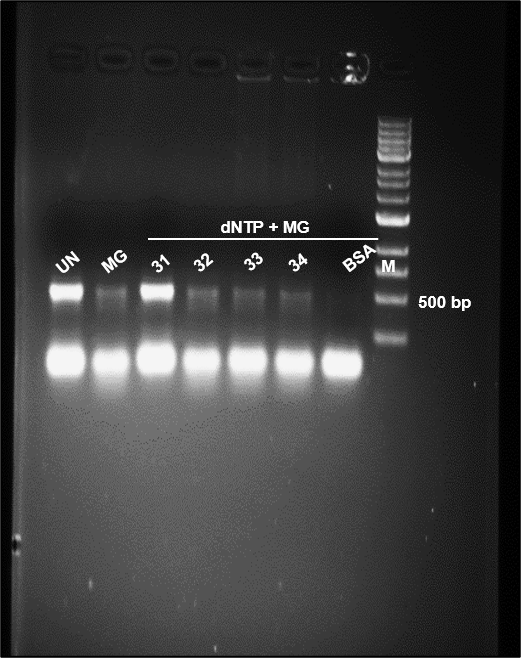

Supplement: Figure 6—source data 1. [file elife-88875-fig6-data1.zip › Figure 6B Original uncropped.tif]

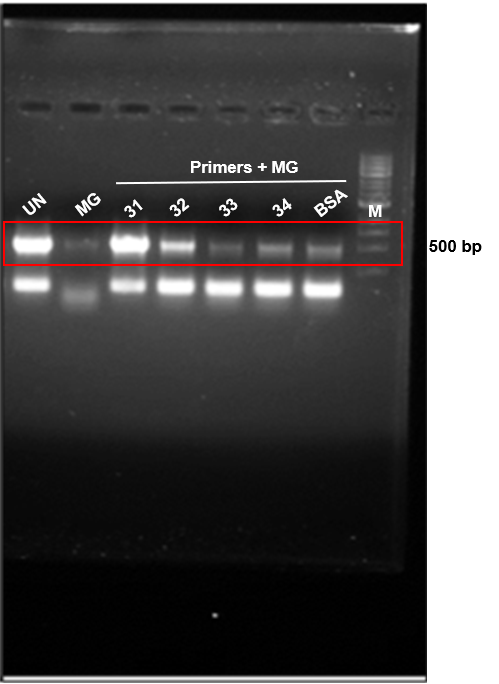

Supplement: Figure 6—source data 2. [file elife-88875-fig6-data2.zip › Figure 6C Cropped labelled.tif]

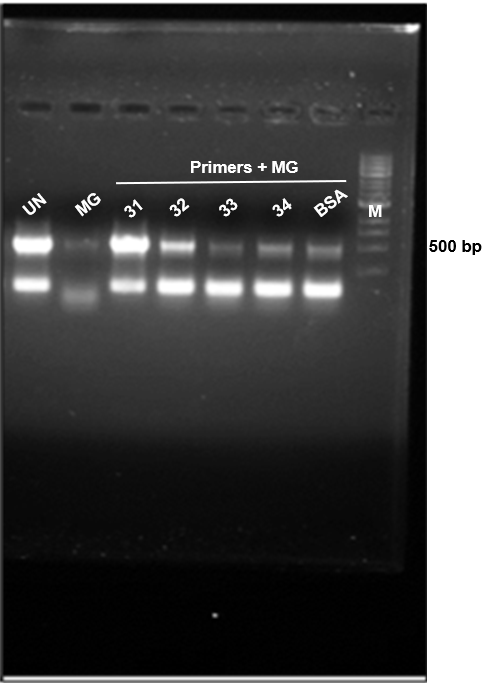

Supplement: Figure 6—source data 2. [file elife-88875-fig6-data2.zip › Figure 6C Original uncropped.tif]

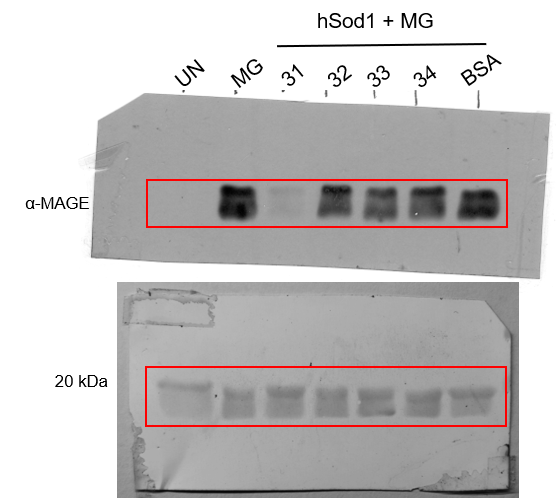

Supplement: Figure 6—source data 3. [file elife-88875-fig6-data3.zip › Figure 6D Cropped labelled.tif]

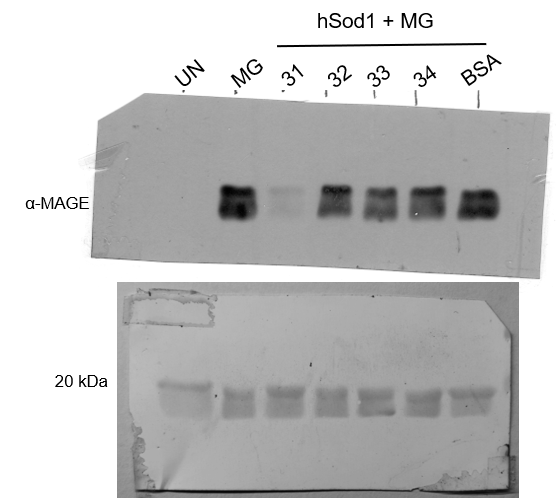

Supplement: Figure 6—source data 3. [file elife-88875-fig6-data3.zip › Figure 6D Original uncropped.tif]

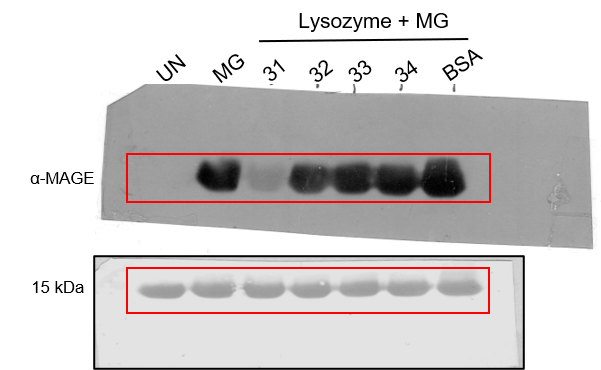

Supplement: Figure 6—source data 4. [file elife-88875-fig6-data4.zip › Figure 6E Cropped labelled.tif]

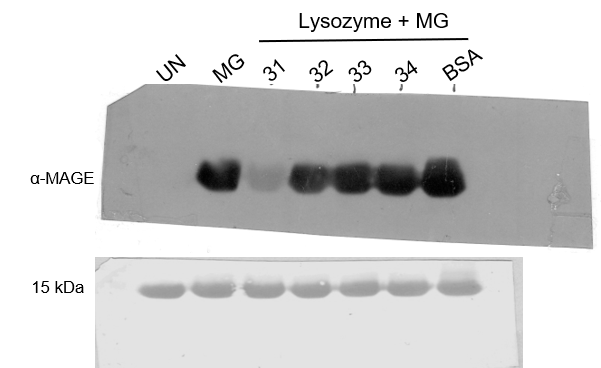

Supplement: Figure 6—source data 4. [file elife-88875-fig6-data4.zip › Figure 6E Original uncropped.tif]

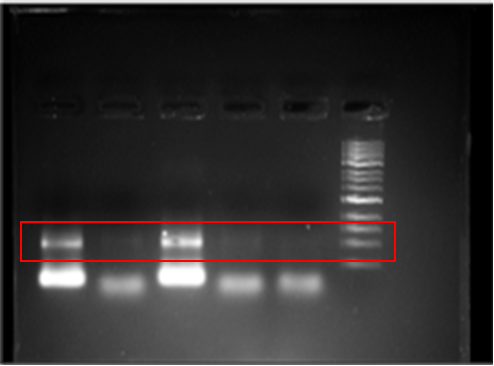

Supplement: Figure 6—figure supplement 1—source data 1. [file elife-88875-fig6-figsupp1-data1.zip › Figure S6A Cropped labelled.tif]

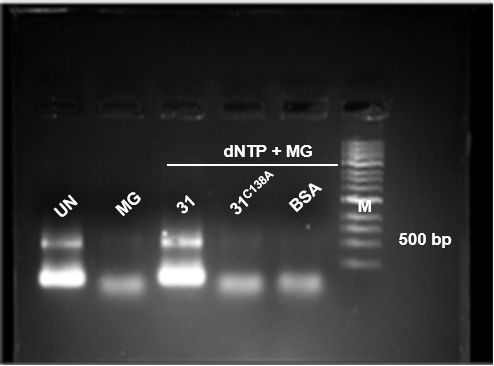

Supplement: Figure 6—figure supplement 1—source data 1. [file elife-88875-fig6-figsupp1-data1.zip › Figure S6A Original uncropped.tif]

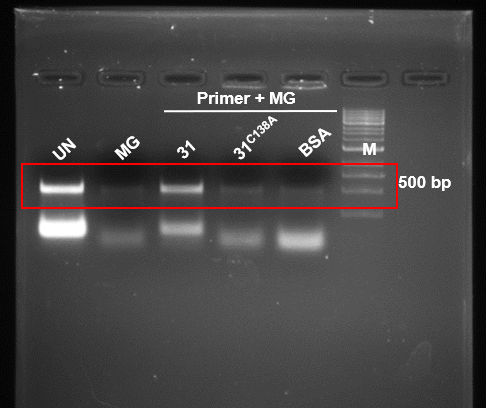

Supplement: Figure 6—figure supplement 1—source data 2. [file elife-88875-fig6-figsupp1-data2.zip › Figure S6B Cropped labelled.tif]

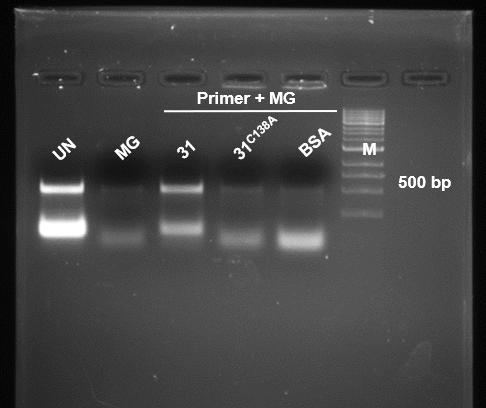

Supplement: Figure 6—figure supplement 1—source data 2. [file elife-88875-fig6-figsupp1-data2.zip › Figure S6B Original uncropped.tif]

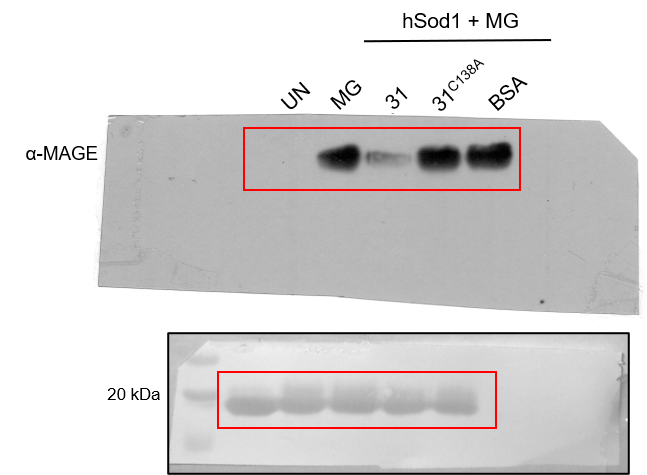

Supplement: Figure 6—figure supplement 1—source data 3. [file elife-88875-fig6-figsupp1-data3.zip › Figure S6C Cropped labelled.tif]

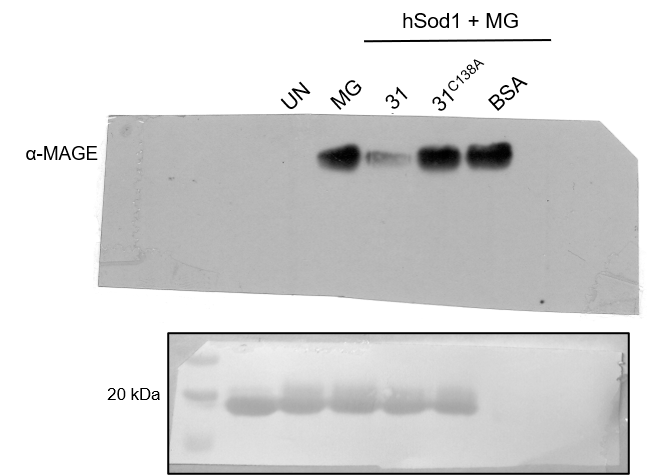

Supplement: Figure 6—figure supplement 1—source data 3. [file elife-88875-fig6-figsupp1-data3.zip › Figure S6C Original uncropped.tif]

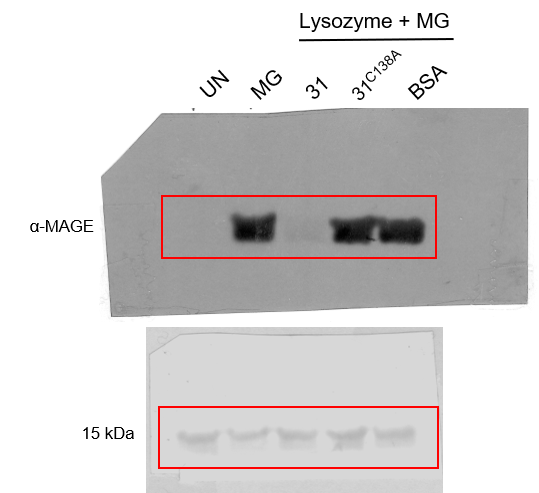

Supplement: Figure 6—figure supplement 1—source data 4. [file elife-88875-fig6-figsupp1-data4.zip › Figure S6D Cropped labelled.tif]

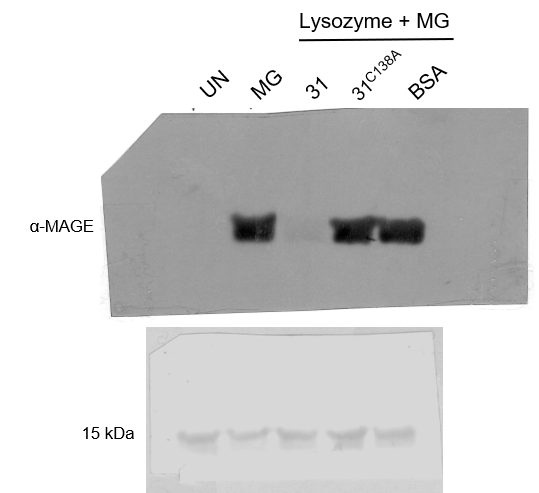

Supplement: Figure 6—figure supplement 1—source data 4. [file elife-88875-fig6-figsupp1-data4.zip › Figure S6D Original uncropped.tif]

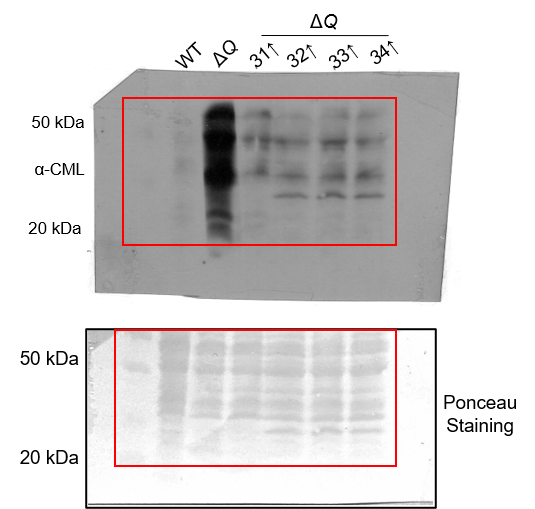

Supplement: Figure 7—source data 1. [file elife-88875-fig7-data1.zip › Figure 7F Cropped labelled.tif]

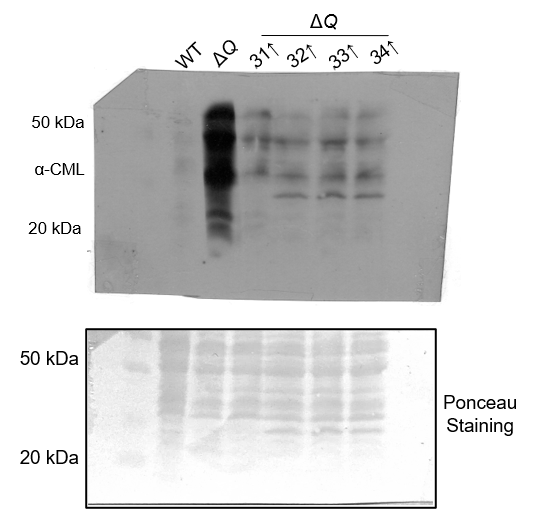

Supplement: Figure 7—source data 1. [file elife-88875-fig7-data1.zip › Figure 7F Original uncropped.tif]

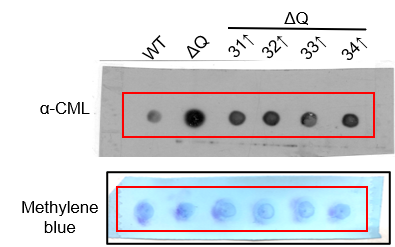

Supplement: Figure 7—source data 2. [file elife-88875-fig7-data2.zip › Figure 7G cropped labelled.tif]

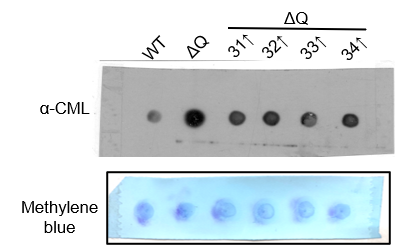

Supplement: Figure 7—source data 2. [file elife-88875-fig7-data2.zip › Figure 7G original uncropped.tif]

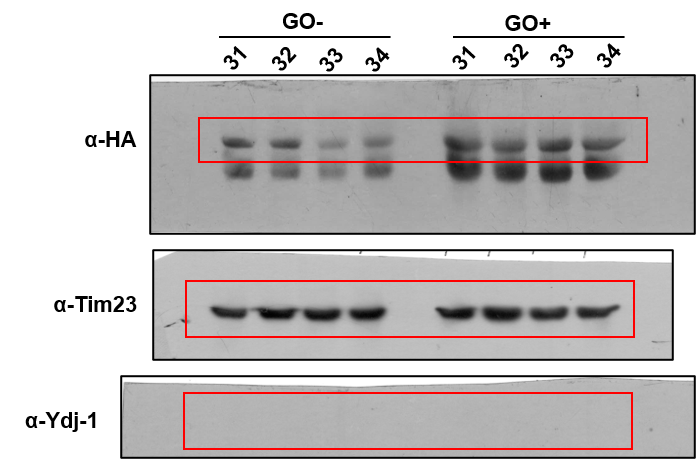

Supplement: Figure 7—figure supplement 1—source data 1. [file elife-88875-fig7-figsupp1-data1.zip › Figure S7A cropped labelled.tif]

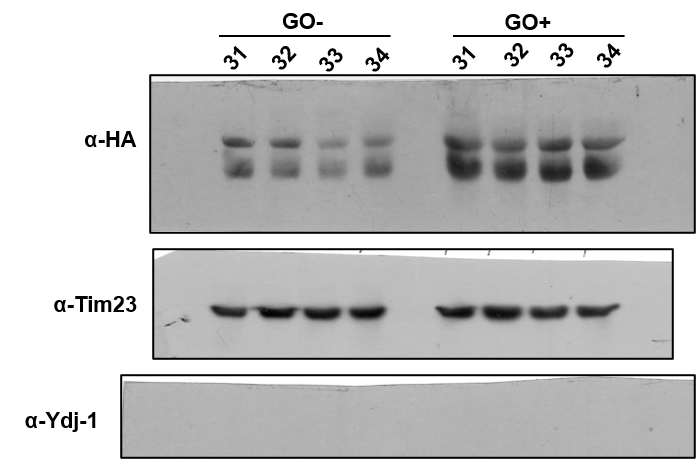

Supplement: Figure 7—figure supplement 1—source data 1. [file elife-88875-fig7-figsupp1-data1.zip › Figure S7A original uncropped.tif]

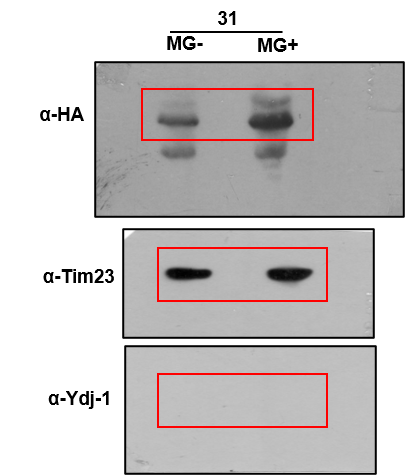

Supplement: Figure 7—figure supplement 1—source data 2. [file elife-88875-fig7-figsupp1-data2.zip › Figure S7B cropped labelled.tif]

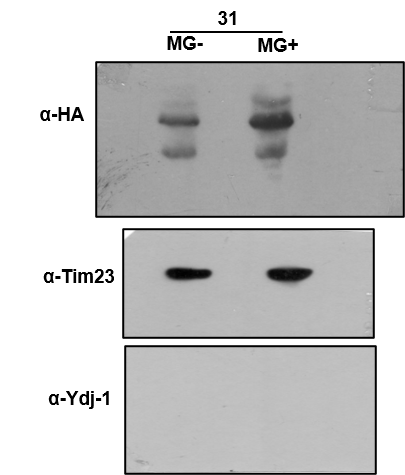

Supplement: Figure 7—figure supplement 1—source data 2. [file elife-88875-fig7-figsupp1-data2.zip › Figure S7B original uncropped.png]

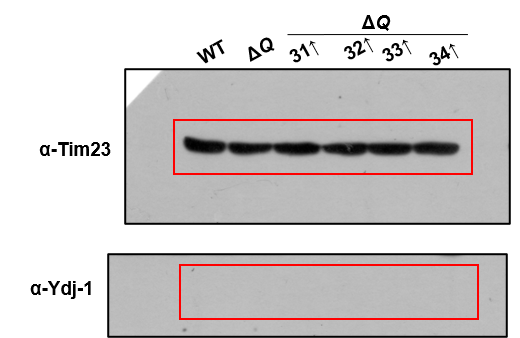

Supplement: Figure 7—figure supplement 1—source data 3. [file elife-88875-fig7-figsupp1-data3.zip › Figure S7F cropped labelled.tif]

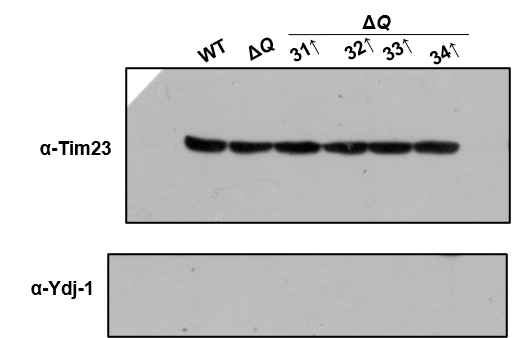

Supplement: Figure 7—figure supplement 1—source data 3. [file elife-88875-fig7-figsupp1-data3.zip › Figure S7F original uncropped.tif]
