# Supplementary material for: Saccharomyces cerevisiae DJ-1 paralogs maintain genome integrity through glycation repair of nucleic acids and proteins
Source: eLife. 2023 Aug 7;12:e88875. doi: 10.7554/eLife.88875 (PMC10431920; doi:10.7554/eLife.88875)
Supplement: Figure 5—source data 5. [file elife-88875-fig5-data5.zip › Figure 5F .pdf]

Mutation profile of SOD1, RAD14, and COX2

Mutations (red) **Original sequence** (yellow) **Fp-Forward primer; Rp-reverse primer**

WT

SOD1

```
WTFP        --------------------------------------ACTGGGTCCGGGTGTTGGTCAA     22
SOD1        -----ATGGT-TCAAGCAGTCGCAGTGTTAAAGGGTGATGCCGGTGTCTCTGGTGTTGTC     54
WTRP        TTTTTATGGTTTCAAGCACTCGCAGTGTTAAAGGGTGATGCCGGTGTCTCTGGTGTTGTC     60
                                                      ***     ** ** *

WTFP        GTTTCAAACAGGCTTTCCAAATCCAAGCCAACCACTGTCTCTTACAAGATCGCTGGTAAC     82
SOD1        AAGTTCGAACAGGCTTCCGAATCCGAGCCAACCACTGTCTCTTACGAGATCGCTGGTAAC     114
WTRP        AAGTTCGAACAGGCTTCCGAATCCGAGCCAACCACTGTCTCTTACGAGATCGCTGGTAAC     120
               *   *   *  **** ***** ******************** **************

WTFP        AGTCCTAACGCAAAACGTGGGTTCCACATTCATGAGTTTGGAAATGCCACCAAGGGTTGT     142
SOD1        AGTCCTAACGCAGAACGTGGGTTCCACATTCATGAGTTTGGAGATGCCACCAATGGTTGT     174
WTRP        AGTCCTAACGCAGAACGTGGGTTCCACATTCATGAGTTTGGAGATGCCACCAATGGTTGT     180
            ************ ***************************** ********** ******

WTFP        GTCTCTGCTGGTCCTCACTTCAATCCTTTCAAAAAAACACATGGTGCTCCAACTGACAAA     202
SOD1        GTCTCTGCTGGTCCTCACTTCAATCCTTTCAAGAAGACACATGGTGCTCCAACTGACGAA     234
WTRP        GTCTCTGCTGGTCCTCACTTCAATCCTTTCAAGAAGACACATGGTGCTCCAACTGACGAA     240
            ******************************** ** ********************* **

WTFP        TTCAAACATGTCGGTGACATGGGTAACGTAAAAACGGACAAAAATGGTGTGGCCAAGGGC     262
SOD1        GTCAGACATGTCGGTGACATGGGTAACGTAAAGACGGACGAAAATGGTGTGGCCAAGGGC     294
WTRP        GTCAGACATGTCGGTGACATGGGTAACGTAAAGACGGACGAAAATGGTGTGGCCAAGGGC     300
             *** *************************** ****** ********************

WTFP        TCCTTCAAGGACTCTTTGATCAAGCTTATCGGTCCTACCTCCGTTGTAGGCAAAACCGTC     322
SOD1        TCCTTCAAGGACTCTTTGATCAAGCTTATCGGTCCTACCTCCGTTGTAGGCAGAAGCGTC     354
WTRP        TCCTTCAAGGACTCTTTGATCAAGCTTATCGGTCCTACCTCCGTTGTAGGCAGAAGCGTC     360
            **************************************************** ** ****

WTFP        TTTATCCACCCCGGCCAAAATGACTTAGGTAAGGGTGACACTGAAAAATCTTTGAAAACT     382
SOD1        GTTATCCACGCCGGCCAAGATGACTTAGGTAAGGGTGACACTGAAGAATCTTTGAAGACT     414
WTRP        GTTATCCACGCCGGCCAAGATGACTTAGGTAAGGGTGACACTGAAGAATCTTGAAGACTG     420
             ******** ******** ************************** ******  *

WTFP        GGTAATGCCGGTCCAAAACCACCCTGTGGTGTCATTGGTCTAACCAACAAAAAAAAA        439
SOD1        GGTAATGCCGGTCCAAAACCAGCCTGTGGTGTCATTGGTCTAACCAACTAA------        465
WTRP        GTA-ATGCCGTCCAAGA----------------------------------------        436
            *   ******  * *
```

RAD14

```
WTRADRp     ------------------------------------------------------------    0
WTRADFp     ------------------------------------------------------------    0
RAD14       ATGACTCCCGAACAAAAGGCCAAACTAGTATGTGTGAAAATGATTCTGTGTTTGTATTTT    60


WTRADRp     ------------------------------------------------------------    0
WTRADFp     ------------------------------------------------------------    0
RAD14       AACCGTGGGTTTCTTTTTACTAACAATTTAACGATGAGATGAGCTGTGCAGGAGGCTAAC    120


WTRADRp     ------------------------------------------------------------    0
WTRADFp     ------------------------------------------------------------    0
RAD14       AGGAAATTAGCAATAGAACGGTTAAGAAAAAGGGGAATACTGAGTAGCGACCAATTGAAT    180
```

```
WTRADRp      ------------------------------------------------------------    0
WTRADFp      ------------------------------------------------------------    0
RAD14        CGAATAGAAAGTAGGAATGAACCTTTAAAAACCCGGCCTCTCGCAGTTACTAGTGGCAGC    240

WTRADRp      ------------------------------------------------------------    0
WTRADFp      ------------------------------------------------------------    0
RAD14        AATCGGGATGATAATGCAGCAGCCGCAGTACATGTGCCAAATCATAATGGACAACCGTCT    300

WTRADRp      ------------------------------------------------------------    0
WTRADFp      ------------------------------------------------------------    0
RAD14        GCGCTTGCTAACACTAACACTAACACTACTTCACTTTATGGTAGCGGAGTAGTTGATGGA    360

WTRADRp      ------------GCGTCGGTACTCGACAAAAGGCCAACGGATAGAATCAGACAGAGCATA    47
WTRADFp      -------------------------------------CTGGAATTAT--CGACTAGCAT-    20
RAD14        AGTAAAAGGGATGCGTCGGTACTCGACAAAAGGCCAACGGATAGAATCAGACCTAGCATA    420
                                                  * *.:* :**   ... *****

WTRADRp      AGGAAACAAGATTACATTGAGTACGATTTTCCCCCCATGCAGAACCTGAATGGTGGTTAT    107
WTRADFp      -AGGAACAAGATTACATTGAGTACGATTTTGCCACCATGCAGAACTTGAATGGTGGTTAT    79
RAD14        AGGAAACAAGATTACATTGAGTACGATTTTGCCACCATGCAGAACTTGAATGGTGGTTAT    480
              .*.************************** **.*********** **************

WTRADRp      ATCAACCATAAGGACAAGCTTCCAAATTCTGACTCTACCGATGACCAAGAATTTGAATGT    167
WTRADFp      ATCAACCCTAAGGACAAGCTTCCAAATTCTGACTTTACCGATGACCAAGAATTTGAATCT    139
RAD14        ATCAACCCTAAGGACAAGCTTCCAAATTCTGACTTTACCGATGACCAAGAATTTGAATCT    540
             *******.************************** *********************** *

WTRADRp      GAGTTTGGATCTAAAAAGCAGAAGACACTACAGGACTGGAAAAAGGAACAACTTGAACGG    227
WTRADFp      GAGTTTGGATCTAAAAAGCAGAAGACACTACAGGACTGGAAAAAGGAACAACTTGAACGG    199
RAD14        GAGTTTGGATCTAAAAAGCAGAAGACACTACAGGACTGGAAAAAGGAACAACTTGAACGG    600
             ************************************************************

WTRADRp      AAAATGTTGTACGAAAATGCACCTCCTCCAGAGCATATTTCAAAGGCGCCGAAATGTATT    287
WTRADFp      AAAATGCTGTACGAAAATGCACCTCCTCCAGAGCATATTTCAAAGGCGCCGAAATGTATT    259
RAD14        AAAATGCTGTACGAAAATGCACCTCCTCCAGAGCATATTTCAAAGGCGCCGAAATGTATT    660
             ****** *****************************************************

WTRADRp      GAATGTCATATTAATATTGAGATGGATCCTGTGATACATGATGTGTTCAAGTTACAAGTT    347
WTRADFp      GAATGTCATATTAATATTGAGATGGATCCTGTGCTACATGATGTGTTCAAGTTACAAGTT    319
RAD14        GAATGTCATATTAATATTGAGATGGATCCTGTGCTACATGATGTGTTCAAGTTACAAGTT    720
             *********************************.**************************

WTRADRp      TGTAAACAGTGTTCTAAGGAGCATCCAGAAAAGTATGCATTACTGACGAAAACAGAATGT    407
WTRADFp      TGTAAACAGTGTTCTAAGGAGCATCCAGAAAAGTATGCACTACTGACGAAAACAGAATGT    379
RAD14        TGTAAACAGTGTTCTAAGGAGCATCCAGAAAAGTATGCACTACTGACGAAAACAGAATGT    780
             *************************************** ********************

WTRADRp      AAGGAAGATTACTTTTTACCAGACCCCGAATTGAATGATGAGGATTTCTTTCATAGACTA    467
WTRADFp      AAGGAAGATTACTTTTTAACAGACCCCGAATTGAATGATGAGGATCTCTTTCATAGACTA    439
RAD14        AAGGAAGATTACTTTTTAACAGACCCCGAATTGAATGATGAGGATCTCTTTCATAGACTA    840
             ******************.************************** **************

WTRADRp      GAAAAGCCGAACCCTCATTCGGGGACATTTGCAAGAATGCAACTATTTGTTAGATGTGAA    527
WTRADFp      GAAAAGCCGAACCCTCATTCGGGGACATTTGCAAGAATGCAACTATTTGTTAGATGTGAA    499
RAD14        GAAAAGCCGAACCCTCATTCGGGGACATTTGCAAGAATGCAACTATTTGTTAGATGTGAA    900
             ************************************************************

WTRADRp      GTGGAAGCCTTTGCGTTCAAGAAATGGGGTGGAGAAGAAGGTTTTGATGAGGAATGGCAA    587
WTRADFp      GTGGAAGCCTTTGCGTTCAAGAAATGGGGTGGAGAAGAAGGTTTAGATGAGGAATGGCAA    559
RAD14        GTGGAAGCCTTTGCGTTCAAGAAATGGGGTGGAGAAGAAGGTTTAGATGAGGAATGGCAA    960
             ********************************************:***************
```

```
WTRADRp       CGTCGTGAAGAAGGAAAGGCTCACAGAAGGGAGAAAAAAATACGAAAAGAAAATCAAGGAA    647
WTRADFp       CGTCGTGAAGAAGGAAAGGCTCACAGAAGGGAGAAAAAAATACGAAAAGAAAATCAAGGAA    619
RAD14         CGTCGTGAAGAAGGAAAGGCTCACAGAAGGGAGAAAAAAATACGAAAAGAAAATCAAGGAA    1020
              ************************************************************

WTRADRp       ATGCGACTGAAAACAAGAGCTCAAGAATATATTAATAGATTAAGAGAAAAGAAGCATGGG    707
WTRADFp       ATGCGACTGAAAACAAGAGCTCAAGAATATACTAATAGATTAAGAGAAAAGAAGCATGGG    679
RAD14         ATGCGACTGAAAACAAGAGCTCAAGAATATACTAATAGATTAAGAGAAAAGAAGCATGGG    1080
              ******************************* ****************************

WTRADRp       AAAGCCCATATTCATCATTTTAGTGATCCAGTTGATGGAGGTATTGATGAAGACGGTTAT    767
WTRADFp       AAAGCCCATATTCATCATTTTAGTGATCCAGTTGATGGAGGTATTGATGAAGACGGTTAT    739
RAD14         AAAGCCCATATTCATCATTTTAGTGATCCAGTTGATGGAGGTATTGATGAAGACGGTTAT    1140
              ************************************************************

WTRADRp       CAAAT-CAAAGAAGAAGAG--ACAGAC-GCGTTCC-------------------------    798
WTRADFp       CAAATTCAAAGAAGAAGATGTACAGACTGCGGGCTAGAAACTGAAGAATTTGAAACTTTT    799
RAD14         CAAATTCAAAGAAGAAGATGTACAGACTGCGGGCTAGAAACTGAAGAAATTGACATTTAA    1200
              ***** ************   ****** ***  *

WTRADRp       --- 798
WTRADFp       AAA 802
RAD14         --- 1200
```

COX2

```
QCOXRP       TTTTTTTTTATTGTTAGATTTATTAAGATTACAATTAACAACATTCATTATGAATGATGT    60
QCOXFP       -------------------------------------TTAAAAATTCTTATGAATGATGT    23
COX2         ----------ATGTTAGATTTATTAAGATTACAATTAACAACATTCATTATGAATGATGT    50
                                                  : **.*:* .*************

QCOXRP       ACCAACACCTTATGCATGTTATTTTCAGGATTCAGCAACACCAAATCAAGAAGGTATTTT    120
QCOXFP       ACCAACACCTTATGCATGTTATTTTCAGGATTCAGCAACACCAAATCAAGAAGGTATTTT    83
COX2         ACCAACACCTTATGCATGTTATTTTCAGGATTCAGCAACACCAAATCAAGAAGGTATTTT    110
             ************************************************************

QCOXRP       AGAATTACATGATAATATTATGTTTTATTTATTAGTTATTTTAGGTTTAGTATCTTGAAT    180
QCOXFP       AGAATTACATGATAATATTATGTTTTATTTATTAGTTATTTTAGGTTTAGTATCTTGAAT    143
COX2         AGAATTACATGATAATATTATGTTTTATTTATTAGTTATTTTAGGTTTAGTATCTTGAAT    170
             ************************************************************

QCOXRP       GTTATATACAATTGTTATAACATATTCAAAAAATCCTATTGCATATAAATATATTAAACA    240
QCOXFP       GTTATATACAATTGTTATAACATATTCAAAAAATCCTATTGCATATAAATATATTAAACA    203
COX2         GTTATATACAATTGTTATAACATATTCAAAAAATCCTATTGCATATAAATATATTAAACA    230
             ************************************************************

QCOXRP       TGGACAAACTATTGAAGTTATTTGAACAATTTTTCCAGCTGTAATTTTATTAATTATTGC    300
QCOXFP       TGGACAAACTATTGAAGTTATTTGAACAATTTTTCCAGCTGTAATTTTATTAATTATTGC    263
COX2         TGGACAAACTATTGAAGTTATTTGAACAATTTTTCCAGCTGTAATTTTATTAATTATTGC    290
             ************************************************************

QCOXRP       TTTTCCTTCATTTATTTTATTATATTTATGTGATGAAGTTATTTCACCAGCTATAACTAT    360
QCOXFP       TTTTCCTTCATTTATTTTATTATATTTATGTGATGAAGTTATTTCACCAGCTATAACTAT    323
COX2         TTTTCCTTCATTTATTTTATTATATTTATGTGATGAAGTTATTTCACCAGCTATAACTAT    350
             ************************************************************

QCOXRP       TAAAGCTATTGGATATCAATGATATTGAAAATATGAATATTCAGATTTTATTAATGATAG    420
QCOXFP       TAAAGCTATTGGATATCAATGATATTGAAAATATGAATATTCAGATTTTATTAATGATAG    383
COX2         TAAAGCTATTGGATATCAATGATATTGAAAATATGAATATTCAGATTTTATTAATGATAG    410
             ************************************************************

QCOXRP       TGGTGAAACTGTTGAATTTGAATCATATGTTATTCCTGATGAATTATTAGAAGAAGGTCA    480
QCOXFP       TGGTGAAACTGTTGAATTTGAATCATATGTTATTCCTGATGAATTATTAGAAGAAGGTCA    443
COX2         TGGTGAAACTGTTGAATTTGAATCATATGTTATTCCTGATGAATTATTAGAAGAAGGTCA    470
```

```
              ************************************************************

QCOXRP        ATTAAGATTATTAGATACTGATACTTCTATAGTTGTACCTGTAGATACACATATTAGATT    540
QCOXFP        ATTAAGATTATTAGATACTGATACTTCTATAGTTGTACCTGTAGATACACATATTAGATT    503
COX2          ATTAAGATTATTAGATACTGATACTTCTATAGTTGTACCTGTAGATACACATATTAGATT    530
              ************************************************************

QCOXRP        CGTTGTAACAGCTGCTGATGTTATTCATGATTTTGCTATTCCAAGTTTAGGTATTAAAGT    600
QCOXFP        CGTTGTAACAGCTGCTGATGTTATTCATGATTTTGCTATTCCAAGTTTAGGTATTAAAGT    563
COX2          CGTTGTAACAGCTGCTGATGTTATTCATGATTTTGCTATTCCAAGTTTAGGTATTAAAGT    590
              ************************************************************

QCOXRP        TGATGCTACTCCTGGTAGATTAAATCAAGTTTCTGCTTTAATTCAAAGAGAAGGTGTCTT    660
QCOXFP        TGATGCTACTCCTGGTAGATTAAATCAAGTTTCTGCTTTAATTCAAAGAGAAGGTGTCTT    623
COX2          TGATGCTACTCCTGGTAGATTAAATCAAGTTTCTGCTTTAATTCAAAGAGAAGGTGTCTT    650
              ************************************************************

QCOXRP        CTATGGAGCATGTTCTGAGTTGTGTGGGACCAGGTCATGCAAATATGCCCAATTAAGATC    720
QCOXFP        CTATGGAGCATGTTCTGAGTTGTGTGGGA-CAGGTCATGCAAATATGCC-AATTAAGATC    681
COX2          CTATGGAGCATGTTCTGAGTTGTGTGGGA-CAGGTCATGCAAATATGCC-AATTAAGATC    708
              ***************************** ******************* **********

QCOXRP        GAAGCAGTATCATC--CTTTTTTTT------------------------------ 743
QCOXFP        GAAGCAGTATCATTACCTAAATTTTTGGAATGATTAAATGAAACATTAAAAAAAC 736
COX2          GAAGCAGTATCATTACCTAAATTTTTGGAATGATTAAATGAAAAATAA------- 756
              *************   **:::****
```

ΔQ

RAD14

```
QRAD14Fp      ---------------------AAAGGCCAATGGGTGAAATGAATTCTGTGTTTGTATTTT    39
WTRAD14       ATGACTCCCGAACAAAAGGCCAAACTAGTATGTGTGAAAATGATTCTGTGTTTGTATTTT    60
QRAD14Rp      -----------------------------------------GGGGGTACTTTTTTTTTTT    19
                                                            *   *** * ****

QRAD14Fp      AACCGTGGGT--TTCTTTTTACTAACAATTTAACGATGAGATGA---GCTGTGCAGGAGG    94
WTRAD14       AACCGTGGGT--TTCTTTTTACTAACAATTTAACGATGAGATGA---GCTGTGCAGGAGG    115
QRAD14Rp      AAAAATATATTTTTCTTCTTTTT-CCTGTTTGGGCAGAAGGCCCTTTCCTCATTTTGGGG    78
              **   *   *  ***** **  *  *  ***    *  **        **      * **

QRAD14Fp      CTAACAGGAAATTAGCAATAGAACGGTTAAGAAAAAGGGGAATACTGAGTAGCG----AC    150
WTRAD14       CTAACAGGAAATTAGCAATAGAACGGTTAAGAAAAAGGGGAATACTGAGTAGC---GACC    172
QRAD14Rp      GGGGGGGGCAAGTTCCCTCCAAAC---------CTCAAAGCATCCTTTTTTCCCCCCCCC    129
                    ** ** *  *     ***               * ** **   *  *      *

QRAD14Fp      CAATTGAATCGAATAGAAAGTAGGAATGAACCTTTAAAAACCCGGCCTCTCGCAGTTACT    210
WTRAD14       AAT-TGAATCGAATAGAAAGTAGGAATGAACCTTTAAAAACCCGGCCTCTCGCAGTTACT    231
QRAD14Rp      CCTATTTTTGTAGAGCAAAAGAGGAGGGCTGCTTTTCAC---------------------    168
                  *   *  *    ***  ****  *   ****  *

QRAD14Fp      AGTGGCAGCAATCGGGATGATAATGCAGCAGCCGCAGTACATGTGCCAAATCATAATGGA    270
WTRAD14       AGTGGCAGCAATCGGGATGATAATGCAGCAGCCGCAGTACATGTGCCAAATCATAATGGA    291
QRAD14Rp      ------------------------------------------TTCTAAACTCAAAA---A    183
                                                         *   ** *** **   *

QRAD14Fp      CAACCGTCTGCGCTTGCTAACACTAACACTAACACTACTTCACTTTATGGTAGCGGAGTA    330
WTRAD14       CAACCGTCTGCGCTTGCTAACACTAACACTAACACTACTTCACTTTATGGTAGCGGAGTA    351
QRAD14Rp      AAAAGCTCTCCTCTTTTTAAAAAAAACC------GCGGCGGGGGGGGGGGGGGGGAATTA    237
               **   *** * ***  *** *  * *       *  *          **  * * * **

QRAD14Fp      GTTGATGGAAGTAAAAGGGATGCGTCGGTACTCCACAAAAGGCCAACGGATAGAATCAGA    390
WTRAD14       GTTGATGGAAGTAAAAGGGATGCGTCGGTACTCGACAAAAGGCCAACGGATAGAATCAGA    411
QRAD14Rp      GTGGGTGGGTGAGGAAGAGCGCCCCTCAGACCCCCCCA-CGGGGGGGTGTTTGACAAAAA    296
              ** * ***  *   ***  *   *      ** *  * *  **      * * **   * *
```

```
QRAD14Fp      CCTAGCATATGGAAACACGATTACATTGAGTACGATTTTGTTCTCTTG------------   438
WTRAD14       CCTAGCATAAGGAAACAAGATTA--------------CATTGAGTACGATTTTGCCACCA   457
QRAD14Rp      AATATAATATCTCACCCAATTTTGTTTTTGTCACGCAGGTTGGGCACCCCTCTTCGG---   353
                **  ***    * *    **                  *

QRAD14Fp      -CGAGAACTTGAATAGGAGGAGTATATCACCCCCCTTAGGAGCGAGCCGTCTCCCATTCT   497
WTRAD14       TGCAGAACTTGAATGGTGGTTA--TATCAACC---CTAAGGACAAGCTTCCAAATTC---   509
QRAD14Rp      GGACGCCCTTGGAGAGGTGTTTTACAACAACG---GTAAAGATCATCCACATCTGTTTGC   410
                  *  **** *  *  *      * ** *     **      * *

QRAD14Fp      TGACTATTACCGATGGACCACCCAATTTGTAATCTCGCAGTTTGTGAGCTTTTAAAGCGC   557
WTRAD14       ----TGACTTTACCGATGACCAAGAATTTGA------ATCTGAGTTTGGA-TCTAAAA--   556
QRAD14Rp      CGCCGGGTGCTCCCGCAGAAATTAATATTAA------TTATGAGTTCTGTTTGGGGCCCG   464
                            *         *  *  *         *  **      *

QRAD14Fp      CCCGACACACACTGCGGCTGTGAGAGAAGAGGGGAATCTTGCTACAGAGAGATATCTGTC   617
WTRAD14       AGCAGAAGACACTACAGGACTGGAAAAA-----GGAACAACTTGAACGGAAAATGCTGTA   611
QRAD14Rp      CTGAAGACATTTTCAGGAGAGGAAAAAG-----GAATCTACTCGAAAGTAAACTGCTGTA   519
                    * *   *   *    *  * *      * * *       *   * *   ****

QRAD14Fp      TCTAAAGATGTGTCCTCTCTCCCCAGACACATATTGTCCCAGCGGGCCTCATAAAGTGTC   677
WTRAD14       CGAAAAT----GCACCTCCTCCAGAG---CATATT-TCAAAGGCGCCGAAATGTATTGA-   662
QRAD14Rp      CGAAAAC----GCTCCTCCTCCAGAG---CATATT-TCAAGGGCGCCGAAATGTATTGA-   570
                 ***     *  *   ****  **   ****** **   *  * *   **  * **

QRAD14Fp      CGAGACGGGACATAATAAATCTTGGGGGATCGCCCCCGCGAGCACACATATG--TGGGGG   735
WTRAD14       ----ATGTCATATT---AATATT--GAGATGGATCCTGTGCTACATGATGTGTTCAAGTT   713
QRAD14Rp      ----ATGTCATATT---ATTATT--GAGATGGATCGTGTGCTACATGAGGTGTTCAAGTT   621
                  * *  * **    * * **  * *** *  *  * *       *  **     *

QRAD14Fp      ACCCGTGCCCCCGTGGGGGGGAAGAGCGCCGCCCCCAAGGGCACCTCTCCAGAGGA----   791
WTRAD14       ACAAGTTTGTAAACAGTGTTCTAAGGAGCATCCAGAAAAGTATGCACTACTGACGAAAAC   773
QRAD14Rp      ACAAGTTTGTAAACACTGTTCTAAGGAGCATCCAGAAAAATATGCACTACTGACGAAAAC   681
              **  **           *    *  * **  **   **      * ** * ** **

QRAD14Fp      ----GGGGTGGGCCACAACTTCGGCAAACAAAAAAAAGAAAAATGTGGTGGGCTTTATTT   847
WTRAD14       AGAATGTAAGGAAGATTACTTTTTAACAGA---CCCCGAATTGAATGATGAGGATCTCTT   830
QRAD14Rp      AGAATGTAAGGAAGATTACTTTTTAACAGA---CCCCGAATTGAATGATGAGGATCTCTT   738
                   *   **   *  ****    * * *       ***     ** ** *  *   **

QRAD14Fp      TTATTTTCTTTTTGTTACAAACACCCCCCCCCCCCGGGGGGGGGGTTTGGGGGGGGGGCG   907
WTRAD14       TCA-------TAGACTAGAAAAGCCGAA-CCCTCATTCGGGGACATTTGCA-AGAATGCA   881
QRAD14Rp      TCA-------TAGACTAGAAAAGCCGAA-CCCTCATTCGGGGACATTTGCA-AGAATGCA   789
              * *       *    ** ***  **    *** *    ****   ****    *   **

QRAD14Fp      AACCCTC-----------------------CCCCCAC-------CAAACCCACCTAAATT   937
WTRAD14       ACTATTTGTTAGATGTGAAGTGGAAGCCTTTGCGTTCAAGAAATGGGGTGGAGAAGAAGG   941
QRAD14Rp      ACTATTTGTTAGATGTGAAGTGGAAGCCTTTGCGTTCAAGAAATGGGGTGGAGAAGAAGG   849
              *    *                          *   *              *    **

QRAD14Fp      TTTTCCCCCCCCCC--CCCCCC-----------CTTCCCTCCCCGCGCGCGCCCTACCTT   984
WTRAD14       TTTAGATGAGGAATGGCAACGTCGTGAAGAAGGAAAGGCTCACAGAA-GGGAGAAAAAAT   1000
QRAD14Rp      TTTAGATGAGGAATGGCAACGTCGTGAAGAAGGAAAGGCTCACAGAA-GGGAGAAAAAAT   908
              ***             *  *                  *** * *   * *    *   *

QRAD14Fp      TTAAAAAGAAAAAAAAAGGGAACAGCGCCTATTTTGTGTTTTTGTTTGTTTTGTTTTTATT   1044
WTRAD14       ACGAAAAGAAAATCA-AGGAAATGCGACTGAAAACAAGAGCTCAA------GAATATACT   1053
QRAD14Rp      ACGAAAAGAAAATCA-AGGAAATGCGACTGAAAACAAGAGCTCAA------GAATATACT   961
                 *********  *  ****  *** **            *         *  * ** *

QRAD14Fp      GTGGGTTTAAAAAAAACCCACCACCCCCCTCCCCTCTTTTTTGCCTCTTTTTTCCCCAAA   1104
WTRAD14       AATAGATTAAGAGAAAAGAAGCATGGGAAAGCCCATA-TTCATCATTTAGTGATCCAGT   1112
QRAD14Rp      AATAGATTAAGAGAAAAGAAGCATGGGAAAGCCCATA-TTCATCATTTAGTGATCCAGT   1020
                  * **** * ***   * **        ***    **   * * **  *   ***

QRAD14Fp      AAATATAAAGAGGGGGGGGGGTGGGGGGGGAAAAAAAACAAGGGAGGGG-GGGCGTGG   1163
```

```
WTRAD14         TGATGGAGGTAT------TGATGAAGACGGTTATCAAATTCAAAGAAGAAGATGTACAGA   1166
QRAD14Rp        TGATGGAGGTAT------TGATGAAGACGGTTATTCAAATTCAAAGAAGAAGATGACAGA   1074
                  **  *   *        * **  *  **  *   **    *   *           *

QRAD14Fp        TGCGGGGGGGCGGGGGAGGGGGGGGAAAATTTTTGCTCCCCCCCCCCTCTCCCCTCGGT   1223
WTRAD14         CTGCGGGCTAGAAACTGAAGAAATTGACATTTAA--------------------------   1200
QRAD14Rp        CGCGCCCCCCCTTTT---------------------------------------------   1089
```

SOD1

```
Qsod1Rp         ----TAAAAAAAGGCAAGGGCTTGGAACCGGGCATTACCGGGTCTT-----------CCA   45
QSODFp          ---------------TAACGCTAGAATGTAGCGGTATTCTGGTGTTGTCGAGTTCTCACA   45
WTSOD1          ATGGTTCAAGCAGTCGCAGTGTTAAAGGGTGATGCCGGTGTCTCTGGTGTTGTCAAGTTC   60
                                     *   *    *           * *

qsod1Rp         AAGGATTCTTCAGTGTCACCCATTACCTAAGTCATCTTGGCCGGCGTGGATAACGACGCT   105
QSODFp          ACAGGAATATCCGAATCAAAGCCAACCACTGTCTCTTACGAAATCGCTGGTAACAGTCCT   105
WTSOD1          GAACAGGCTTCCGAATCCGAGCCAACCACTGTCTCTTACGAGATCGCTGGTAACAGTCCT   120
                         ** *  **       ***   ***   *  *    **  * ****    **

qsod1Rp         TCTGCCTACAACGGAGGTAGGACCGATAAGCTTGATCAAAGAGTCCTTTGAAGGAGCCCT   165
QSODFp          AACGCAAAA------CGTGGGTTCCA----------CATTCATGAGTTTGGAAATGCCAC   149
WTSOD1          AACGCAGAA------CGTGGGTTCCA----------CATTCATGAGTTTGGAGATGCCAC   164
                   **  *        ** **  * *          **   *    **** *   ***

qsod1Rp         TGGCCACACCATTTTCGTCCGTCTTTACGTTACCCATGTCACCGACATGTCTGACTTCGT   225
QSODFp          CAATGGTTGTGTCTCTGCTGGTCCTCACTTCAATCCTTTCAAAAAAACACATGGGGCTCC   209
WTSOD1          CAATGGTTGTGTCTCTGCTGGTCCTCACTTCAATCCTTTCAAGAAGACACATGGTGCTCC   224
                           * *  *   *** * ** * *  * * ***   * *     **

qsod1Rp         CAGTTGGAGCACCATGTGTCTTCTTGAAAGGATTGAAGTGAGGACCAGCAGAGACACAAC   285
QSODFp          AACTGACAAAGTCAAACATGTCGGTGACATGGGTAACGTAAAGACGGACAAAAATGGTGT   269
WTSOD1          AACTGACGAAGTCAGACATGTCGGTGACATGGGTAACGTAAAGACGGACGAAAATGGTGT   284
                 * *        **    * *   *** * *  * * ** * ***   *  * *

qsod1Rp         C-----------ATT--GGTGGCATCTCCAAACTCATGAATGTGGAACCCACGTTCTGCG   332
QSODFp          GGCCAAAGGCTCCTTCAAGGGCCCTTTGATAAATCTTATCGGTCCTACCCCCGTTGTAAG   329
WTSOD1          GGCCAAGGGCTCCTTCAAGGACTCTTTGATCAAGCTTATCGGTCCTACCTCCGTTGTAGG   344
                             **   *     * *    *  * *    **   ***  **** *  *

qsod1Rp         TTAGGACTGTTACCAGCGATCTCGTAAGAAACAGTGGTTGGCTCGGATTCCGAAACCTGG   392
QSODFp          CCAAAACCGTCTTTACCGACCCCGGCAAAGACACTTTATGTATGGTAGAACGAA--CAAG   387
WTSOD1          CAGAAGCGTCGTTATCCACGCCGGCCAAGATGACTTAGGTAAGGGTGACACTGA--AGAA   402
                      *         *   *  *  *     * *         *     *  *

qsod1Rp         TCTAACTTGACGACACCTGGAGGCTCCGGGATCAGCCTGTTGACGACAAAGAATGGATTG   452
QSODFp          CCTTAACTT-ACCGACACGCA-GACCCCCGCCCCCCCTGTGGTGCTTTT---TTTGCTTG   442
WTSOD1          TCTTTGAAGACTGGTAATGCC-GGTCCAAGACCAGCCTGTGGTGTCATT---GG--TCTA   456
                 **               *   *  **  *  *  ***** *                *

qsod1Rp         AAGAAAAAA    461
QSODFp          ACCAATGAA    451
WTSOD1          ACCAACTAA    465
                *  **  **
```

COX2

```
QCOXFp          -----------------------------------TAATATCCCGGCATTCCCTGATCTT   25
QCOXRp          TTTTTTTTTTTTTTTATTGTTAGATTTATTAAGATTACAATTAACAACATTCATTATGAA   60
COX2            ----------------ATGTTAGATTTATTAAGATTACAATTAACAACATTCATTATGAA   44
                                                   **  **        * * * **

QCOXFp          TGAGATACTTGCGTGTCCTGCTA---TACCTCAGCTGTCATGCTGGGCCGTGGAATAGGC   82
QCOXRp          TGATGTACCAACACATTATGCATGTTATTTTCAGGATTCAGCAACACCAAATCAAGAAGG   120
COX2            TGATGTACCAACACCTTATGCATGTTATTTTCAGGATTCAGCAACACCAAATCAAGAAGG   104
                ***  ***   *   *  ***         ****   ***     *     ** * *

QCOXFp          GCCCTTTTTAAAACAGGTTTAGGA--TGTTT-----------TTATCTAAGGGAAGTCCC   129
```

```
QCOXRp      TATCTTAGAATTACATGATAATATTATGTTTATTTATTAGTTATTTTAGGTTTAGTATC     180
COX2        TATTTTAGAATTACATGATAATATTATGTTTATTTATTAGTTATTTTAGGTTTAGTATC     164
                **   *  *** * * *     *****           *  * ** *   ***  *

QCOXFp      TGGATTGCCCCGGCAACATGGATTATCTCAACCATGTCTTACCCCCCCTCGTGTTCCAAC    189
QCOXRp      TTGAATGTTATATA------CAATTGTTATAACATATTCAAAAAATCCTATTGCATATAA    234
COX2        TTGAATGTTATATA------CAATTGTTATAACATATTCAAAAAATCCTATTGCATATAA    218
            * ** **              * *   *  * *** *   *     ***  **     *

QCOXFp      GTGTCAACACCGCGTGTCGACCCGTTGAAACATGAGTGAACCATCTATTA----------    239
QCOXRp      ATATATT--AAACATGGACAAACTATTGAAGTTATTTGAACAATTTTTCCAGCTGTAATT    292
COX2        ATATATT--AAACATGGACAAACTATTGAAGTTATTTGAACAATTTTTCCAGCTGTAATT    276
             * *        * **   *  *  *  **  *   ***** ** * *

QCOXFp      CCAGTAGAGAACGTCCCCTCGTGACTTCTCACAAATAATAGATGTGTTATCCTCGTTCCT    299
QCOXRp      TTATTAATTATTGCTTTTCCTTCATTTATTTTATTATATTTATGTGATGAAGTTATTTCA    352
COX2        TTATTAATTATTGCTTTTCCTTCATTTATTTTATTATATTTATGTGATGAAGTTATTTCA    336
              * **   *  *      * * * ** *   *    **  ***** *    *  ** *

QCOXFp      TCCTGAGAAATTTTTGTTAATATCCGATGAAAAATATTTTATTAT----CATAATCGGCC    355
QCOXRp      CCAGCTATAACTATTAAAGCTATTGGATATCAATGATATTGAAAATATGAATATTCAGAT    412
COX2        CCAGCTATAACTATTAAAGCTATTGGATATCAATGATATTGAAAATATGAATATTCAGAT    396
             *      ** * **     ***  ***   **  ** **   *      *** ** *

QCOXFp      TATGTTTATAATAATAATACCTGCGGACAGAGCTCCTCCCCTTAGCTTCCTGATTGCTAT    415
QCOXRp      TTTATTAATGATAGTGGTGAAACTGTTGAATTTGAATCATATGTTATTCCTGATGAATTA    472
COX2        TTTATTAATGATAGTGGTGAAACTGTTGAATTTGAATCATATGTTATTCCTGATGAATTA    456
            * * ** ** *** *  *      *   *       **   *    ********   *

QCOXFp      GCTGAGAGAATCGAGTGTAGGTTATTTCAACCCTGAAGACATGCTTCAAAAATCTGACAC    475
QCOXRp      TTAGAAGAAGGTCAATTAAGATTATTAGATACTGATACTTCTATAGTTGTACCTGTAGAT    532
COX2        TTAGAAGAAGGTCAATTAAGATTATTAGATACTGATACTTCTATAGTTGTACCTGTAGAT    516
               **   *    * *  ** *****  *  *    *    *        *     * *

QCOXFp      ACCGATGACCGATAATTTGAATCAGATGCTGGAGCTATAAATGAATATACTCTACCAGAT    535
QCOXRp      ACACATATTAGATTCGTTGTAACAGCTGCTGATGTTATTCATGATTTTGCTATTCCAAGT    592
COX2        ACACATATTAGATTCGTTGTAACAGCTGCTGATGTTATTCATGATTTTGCTATTCCAAGT    576
            **  **    ***   *** * *** *****  * ***  **** * * ** * ***  *

QCOXFp      GG----------------------------------------------------------    537
QCOXRp      TTAGGTATTAAAGTTGATGCTACTCCTGGTAGATTAAATCAAGTTTCTGCTTTAATTCAA    652
COX2        TTAGGTATTAAAGTTGATGCTACTCCTGGTAGATTAAATCAAGTTTCTGCTTTAATTCAA    636


QCOXFp      ---------------------------------------GAAAAAGGTACAACTTGAACG    558
QCOXRp      AGAGAAGGTGTCTTCTATGGAGCATGTTCTGAGTTGTGTGGGACAGGTCATGCAAATATG    712
COX2        AGAGAAGGTGTCTTCTATGGAGCATGTTCTGAGTTGTGTGGGACAGGTCATGCAAATATG    696
                                                   *  * ****    *    * *

QCOXFp      GCCAA-----------TGCTGTATATAACGCTCCTCC-----------------------    584
QCOXRp      CCCAATTAAGATCGAAGCCAGTATCATA--------------------------------    740
COX2        CCAATTAAGATCGAAGCAGTATCATTACCTAAATTTTTGGAATGATTAAATGAACAATAA    756
             * *                 *
```

Hsp31

RAD14

```
31RADRp      ------------------------------------------------------------    0
RAD14        ATGACTCCCGAACAAAAGGCCAAACTAGTATGTGTGAAAATGATTCTGTGTTTGTATTTT    60
31RADFp      --------------------------------------------------------TTTT    4


31RADRp      ------------------------------------------------------------    0
RAD14        AACCGTGGGTTTCTTTTTACTAACAATTTAACGATGAGATGAGCTGTGCAGGAGGCTAAC    120
31RADFp      AACCGTGGGTTTCTTTTTACTAACAATTTAACGATGAGATGAGCTGTGCAGGAGGCTAAC    64
```

```
31RADRp      ------------------------------------------------------------    0
RAD14        AGGAAATTAGCAATAGAACGGTTAAGAAAAAGGGGAATACTGAGTAGCGACCAATTGAAT    180
31RADFp      AGGAAATTAGCAATAGAACGGTTAAGAAAAAGGGGAATACTGAGTAGCGACCAATTGAAT    124

31RADRp      ------------------------------------------------------------    0
RAD14        CGAATAGAAAGTAGGAATGAACCTTTAAAAACCCGGCCTCTCGCAGTTACTAGTGGCAGC    240
31RADFp      CGAATAGAAAGTAGGAATGAACCTTTAAAAACCCGGCCTCTCGCAGTTACTAGTGGCAGC    184

31RADRp      ------------------------------------------------------------    0
RAD14        AATCGGGATGATAATGCAGCAGCCGCAGTACATGTGCCAAATCATAATGGACAACCGTCT    300
31RADFp      AATCGGGATGATAATGCAGCAGCCGCAGTACATGTGCCAAATCATAATGGACAACCGTCT    244

31RADRp      ----------------ACACTAACACTACTTCACTTTATGGTAGCGGAGTAGTTGATGGA    44
RAD14        GCGCTTGCTAACACTAACACTAACACTACTTCACTTTATGGTAGCGGAGTAGTTGATGGA    360
31RADFp      GCGCTTGCTAACACTAACACTAACACTACTTCACTTTATGGTAGCGGAGTAGTTGATGGA    304
                             ********************************************

31RADRp      AGTAAAAGGGATGCGTCGGTACTCGACAAAAGGCCAACGGATAGAATCAGACCTAGCATA    104
RAD14        AGTAAAAGGGATGCGTCGGTACTCGACAAAAGGCCAACGGATAGAATCAGACCTAGCATA    420
31RADFp      AGTAAAAGGGATGCGTCGGTACTCGACAAAAGGCCAACGGATAGAATCAGACCTAGCATA    364
             ************************************************************

31RADRp      AGGAAACAAGATTACATTGAGTACGATTTTGCCACCATGCAGAACTTGAATGGTGGTTAT    164
RAD14        AGGAAACAAGATTACATTGAGTACGATTTTGCCACCATGCAGAACTTGAATGGTGGTTAT    480
31RADFp      AGGAAACAAGATTACATTGAGTACGATTTTGCCACCATGCAGAACTTGAATGGTGGTTAT    424
             ************************************************************

31RADRp      ATCAACCCTAAGGACAAGCTTCCAAATTCTGACTTTACCGATGACCAAGAATTTGAATCT    224
RAD14        ATCAACCCTAAGGACAAGCTTCCAAATTCTGACTTTACCGATGACCAAGAATTTGAATCT    540
31RADFp      ATCAACCCTAAGGACAAGCTTCCAAATTCTGACTTTACCGATGACCAAGAATTTGAATCT    484
             ************************************************************

31RADRp      GAGTTTGGATCTAAAAAGCAGAAGACACTACAGGACTGGAAAAAGGAACAACTTGAACGG    284
RAD14        GAGTTTGGATCTAAAAAGCAGAAGACACTACAGGACTGGAAAAAGGAACAACTTGAACGG    600
31RADFp      GAGTTTGGATCTAAAAAGCAGAAGACACTACAGGACTGGAAAAAGGAACAACTTGAACGG    544
             ************************************************************

31RADRp      AAAATGCTGTACGAAAATGCACCTCCTCCAGAGCATATTTCAAAGGCGCCGAAATGTATT    344
RAD14        AAAATGCTGTACGAAAATGCACCTCCTCCAGAGCATATTTCAAAGGCGCCGAAATGTATT    660
31RADFp      AAAATGCTGTACGAAAATGCACCTCCTCCAGAGCATATTTCAAAGGCGCCGAAATGTATT    604
             ************************************************************

31RADRp      GAATGTCATATTAATATTGAGATGGATCCTGTGCTACATGATGTGTTCAAGTTACAAGTT    404
RAD14        GAATGTCATATTAATATTGAGATGGATCCTGTGCTACATGATGTGTTCAAGTTACAAGTT    720
31RADFp      GAATGTCATATTAATATTGAGATGGATCCTGTGCTACATGATGTGTTCAAGTTACAAGTT    664
             ************************************************************

31RADRp      TGTAAACAGTGTTCTAAGGAGCATCCAGAAAAGTATGCACTACTGACGAAAACAGAATGT    464
RAD14        TGTAAACAGTGTTCTAAGGAGCATCCAGAAAAGTATGCACTACTGACGAAAACAGAATGT    780
31RADFp      TGTAAACAGTGTTCTAAGGAGCATCCAGAAAAGTATGCACTACTGACGAAAACAGAATGT    724
             ************************************************************

31RADRp      AAGGAAGATTACTTTTTAACAGACCCCGAATTGAATGATGAGGATCTCTTTCATAGACTA    524
RAD14        AAGGAAGATTACTTTTTAACAGACCCCGAATTGAATGATGAGGATCTCTTTCATAGACTA    840
31RADFp      AAGGAAGATTACTTTTTAACAGACCCCGAATTGAATGATGAGGATCTCTTTCATAGACTA    784
             ************************************************************

31RADRp      GAAAAGCCGAACCCTCATTCGGGGACATTTGCAAGAATGCAACTATTTGTTAGATGTGAA    584
RAD14        GAAAAGCCGAACCCTCATTCGGGGACATTTGCAAGAATGCAACTATTTGTTAGATGTGAA    900
31RADFp      GAAAAGCCGAACCCTCATTCGGGGACATTTGCAAGAATGCAACTATTTGTTAGATGTGAA    844
             ************************************************************

31RADRp      GTGGAAGCCTTTGCGTTCAAGAAATGGGGTGGAGAAGAAGGTTTAGATGAGGAATGGCAA    644
```

```
RAD14        GTGGAAGCCTTTGCGTTCAAGAAATGGGGTGGGGAAGAAGGTTTAGATGAGGAATGGCAA   960
31RADFp      GTGGA-------------------------------------------------------   849
             *****

31RADRp      CGTCGTGAAGAAGGAAAGGCTCACAGAAGGGAGAAAAAATACGAAAAGAAAATCAAGGAA   704
RAD14        CGTCGTGAAGAAGGAAAGGCTCACAGAAGGGAGAAAAAATACGAAAAGAAAATCAAGGAA   1020
31RADFp      ------------------------------------------------------------   849

31RADRp      ATGCGACTGAAAACAAGAGCTCAAGAATATACTAATAGATTAAGAGAAAAGAAGCATGGG   764
RAD14        ATGCGACTGAAAACAAGAGCTCAAGAATATACTAATAGATTAAGGGAAAAGAAGCATGGG   1080
31RADFp      ------------------------------------------------------------   849

31RADRp      AAAGCCCATATTCATCATTTTAGTGATCCAGTTGATGGAGGTATTGATGAAGACGGTTAT   824
RAD14        AAAGCCCATATTCATCATTTTAGTGATCCAGTTGATGGAGGTATTGATGAAGACGGTTAT   1140
31RADFp      ------------------------------------------------------------   849

31RADRp      CAAATCAA----------------------------------------------------   832
RAD14        CAAATTCAAAGAAGAAGATGTACAGACTGCGGGCTAGAAACTGAAGAAATTGACATTTAA   1200
31RADFp      ------------------------------------------------------------   849
```

SOD1

```
31SOD1Rp      TCAAGGTTCAAAAGAGTCGCAGTGTTAAAGGGTGATGCCGGTGTCTCTGGTGTTGTCAAG   60
SOD1          ---ATGGTTCAAGCAGTCGCAGTGTTAAAGGGTGATGCCGGTGTCTCTGGTGTTGTCAAG   57
31SOD1Fp      -------------------------------GTGAGCCGGGTGC--CTCTGGTGTTGTGG   27
                                             ****  * ****   **   **  *   *

31SOD1Rp      TTCGAACAGGCTTCCGAATCCGAGCCAACCACTGTCTCTTACGAGATCGCTGGTAACAGT   120
SOD1          TTCGAACAGGCTTCCGAATCCGAGCCAACCACTGTCTCTTACGAGATCGCTGGTAACAGT   117
31SOD1Fp      GTTCTAGAGGTTTCCAAAACAGAGCCAACCACTGTCTCTTACAAAATCGCTGGTAACAGT   87
               *   * *** **** ** * ********************* * ***************

31SOD1Rp      CCTAACGCAGAACGTGGGTTCCACATTCATGAGTTTGGAGATGCCACCAATGGTTGTGTC   180
SOD1          CCTAACGCAGAACGTGGGTTCCACATTCATGAGTTTGGAGATGCCACCAATGGTTGTGTC   177
31SOD1Fp      CCTAACGCAAAACGGGGGTTCCACATTCATGATTTTGGAAATGCCACCAAGGGTTGTGTC   147
              ********* **** ***************** ****** ********** *********

31SOD1Rp      TCTGCTGGTCCTCACTTCAATCCTTTCAAGAAGACACATGGTGCTCCAACTGACGAAGTC   240
SOD1          TCTGCTGGTCCTCACTTCAATCCTTTCAAGAAGACACATGGTGCTCCAACTGACGAAGTC   237
31SOD1Fp      TCTGCTGGTCCTCACTTCAATCCTTTCAAAAAAACACATGGGGCTCCAACTGACAAATTC   207
              ***************************** ** ******** ************ ** **

31SOD1Rp      AGACATGTCGGTGACATGGGTAACGTAAAGACGGACGAAAATGGTGTGGCCAAGGGCTCC   300
SOD1          AGACATGTCGGTGACATGGGTAACGTAAAGACGGACGAAAATGGTGTGGCCAAGGGCTCC   297
31SOD1Fp      AAACTTGTCGGTGACATGGGTAACGTAAAGACGGACAAAAATGGTGTGGCCAAGGGCTCC   267
              * ** ******************************* ***********************

31SOD1Rp      TTCAAGGACTCTTTGATCAAGCTTATCGGTCCTACCTCCGTTGTAGGCAGAAGCGTCGTT   360
SOD1          TTCAAGGACTCTTTGATCAAGCTTATCGGTCCTACCTCCGTTGTAGGCAGAAGCGTCGTT   357
31SOD1Fp      TTCAAGGACTCTTTGATCAAGCTTATCGGTCCTACCTCCGTTGTAGGCAAAACCGTCTTT   327
              ************************************************* ** **** **

31SOD1Rp      ATCTCACGCCGGCCAAGATGACTTAGGTAATAGGTGATCACAGCAAGTACATCAGCAAGA   420
SOD1          ATCCACGCCGGCCA-AGATGACTTAGGTAAGGGTGACACTGAAGAATCTTTGAAGACTGG   416
31SOD1Fp      ATCCACCCCGGCCA-AAATGACTTAGGTAAGGGTGACACTGAAAAATCTTTGAAAACTGG   386
              ***     * * *  * *************  *     *  *  **       *    *

31SOD1Rp      CTATGATAACGTAGAAGAATC------------------------------   441
SOD1          TAATGCCGGTCCAAGACCAGCCTGTGGTGTCATTGGTCTAACCAACTAA--   465
31SOD1Fp      TAATGCCGGTCCAAAACCACCCTGTGGTGTCATTGGTCTAACCAACTAAAA   437
                ***       *  *  * *
```

COX2

```
31COXRp      TTTTGTTTTTTTTTTATTTTAGATTTATTAAGATTACAATTAACAACATTCATTATGAAT   60
```

```
31COXFp      ------------------------------------------------------------    0
COX          ---------------ATGTTAGATTTATTAAGATTACAATTAACAACATTCATTATGAAT    45

31COXRp      GATGTACCAACACCTTATGCATGTT-ATTTTCAGGATTCAGCAACACCAAATCAAGAAGG    119
31COXFp      ---TTACCAACACCTTATGCATGTTTATTTTCAGGATTCAGCAACACCAAATCAAGAAGG    57
COX          GATGTACCAACACCTTATGCATGTT-ATTTTCAGGATTCAGCAACACCAAATCAAGAAGG    104
                 ********************* **********************************

31COXRp      TATTTTAGAATTACATGATAATATTATGTTTTATTTATTAGTTATCTTAGGTTTAGTATC    179
31COXFp      TATTTTAGAATTACATGATAATATTATGTTTTATTTATTAGTTATTTTAGGTTTAGTATC    117
COX          TATTTTAGAATTACATGATAATATTATGTTTTATTTATTAGTTATTTTAGGTTTAGTATC    164
             ********************************************* **************

31COXRp      TTGAATGTTATATACAATTGTTATAACATATTCAAAAAATCCTATTGCATATAAATATAT    239
31COXFp      TTGAATGTTATATACAATTGTTATAACATATTCAAAAAATCCTATTGCATATAAATATAT    177
COX          TTGAATGTTATATACAATTGTTATAACATATTCAAAAAATCCTATTGCATATAAATATAT    224
             ************************************************************

31COXRp      TAAACATGGACAAACTATTGAAGTTATTTGAACAATTTTTCCAGCTGTAATTTTATTAAT    299
31COXFp      TAAACATGGACAAACTATTGAAGTTATTTGAACAATTTTTCCAGCTGTAATTTTATTAAT    237
COX          TAAACATGGACAAACTATTGAAGTTATTTGAACAATTTTTCCAGCTGTAATTTTATTAAT    284
             ************************************************************

31COXRp      TATTGCTTTTCCTTCATTTATTTTATTATATTTATGTGATGAAGTTATTTCACCAGCTAT    359
31COXFp      TATTGCTTTTCCTTCATTTATTTTATTATATTTATGTGATGAAGTTATTTCACCAGCTAT    297
COX          TATTGCTTTTCCTTCATTTATTTTATTATATTTATGTGATGAAGTTATTTCACCAGCTAT    344
             ************************************************************

31COXRp      AACTATTAAAGCTATTGGATATCAATGATATTGAAAATATGAATATTCAGATTTTATTAA    419
31COXFp      AACTATTAAAGCTATTGGATATCAATGATATTGAAAATATGAATATTCACATTTTATTAA    357
COX          AACTATTAAAGCTATTGGATATCAATGATATTGAAAATATGAATATTCAGATTTTATTAA    404
             ************************************************* **********

31COXRp      TGATAGTGGTGAAACTGTTGAATTTGAATCATATGTTATTCCTGATGAATTATTAGAAGA    479
31COXFp      TGATAGTGGTGAAACTGTTGAATTTGAATCATATGTTATTCCTGATGAATTATTAGAAGA    417
COX          TGATAGTGGTGAAACTGTTGAATTTGAATCATATGTTATTCCTGATGAATTATTAGAAGA    464
             ************************************************************

31COXRp      AGGTCAATTAAGATTATTAGATACTGATACTTCTATAGTTGTACCTGTAGATACACATAT    539
31COXFp      AGGTCAATTAAGATTATTACATACTGATACTTCGATAGTTGTACCTGTAGATACACATAT    477
COX          AGGTCAATTAAGATTATTAGATACTGATACTTCTATAGTTGTACCTGTAGATACACATAT    524
             ******************* ************* **************************

31COXRp      CAGATTCGTTGTAACAGCTGCTGATGTTATTCATGATTTTGCTATTCCAAGTTTAGGTAT    599
31COXFp      TATATTCGTTGTAACAGCTGCTGATGTTATTCATGATTTTGCTATTCCTAGTTTAGGTAT    537
COX          TAGATTCGTTGTAACAGCTGCTGATGTTATTCATGATTTTGCTATTCCAAGTTTAGGTAT    584
              * *********************************************:***********

31COXRp      TAAAGTTGATGCTACTCCTGGTAGATTAAATCAAGTTTCTGCTTTAATTCAAAGAGAAGG    659
31COXFp      TACAGTAGACGCTACTCCAGGTAGATTAAATCAAGTTTCTGCTTTAATTCAGAGATTCTG    597
COX          TAAAGTTGATGCTACTCCTGGTAGATTAAATCAAGTTTCTGCTTTAATTCAAAGAGAAGG    644
             **.***:** ********:********************************.*** :. *

31COXRp      TGTCTTCTATGGAGCATGTTCTGAGTTGTGTGGGACAGGTCATGCAAATATGCCCAATTA    719
31COXFp      TGTCTTCTACGTGACATGTTGTGACTTATGCCGGACAAGTCATGCAAATATGCGTTGTAA    657
COX          TGTCTTCTATGGAGCATGTTCTGAGTTGTGTGGGACAGGTCATGCAAATATGCCAATTAA    704
             ********* * ..****** *** **.**  *****.***************  : *:*

31COXRp      AGATCGA-AGCAGTTCCAT--CCTT-----------------------------------    741
31COXFp      -CATCAATACCAGTATCATTACCTAAATTGTTGGAATGATAATTGAAAACAATAAAAAAA    716
COX          -GATCGA-AGCAGTATCATTACCTAAATTTTTGGAATGATTAAATG-AACAATAA-----    756
               ***.* * ****: ***  ***:

31COXRp      -----        741
31COXFp      AAAAA        721
COX          -----        756
```

Hsp32

RAD14

```
32RADFp     ----------------------------------------------------------TT   2
RAD14       ATGACTCCCGAACAAAAGGCCAAACTAGTATGTGTGAAAATGATTCTGTGTTTGTATTTT   60
32RADRp     ------------------------------------------------------------   0


32RADFp     TACCGTGGGTTTCTTTTTACTAACAATTTAACGATGAGATGAGCTGTGCAGGAGGCTAAC   62
RAD14       AACCGTGGGTTTCTTTTTACTAACAATTTAACGATGAGATGAGCTGTGCAGGAGGCTAAC   120
32RADRp     ------------------------------------------------------------   0


32RADFp     AGGAAATTAGCAATAGAACGGTTAAGAAAAAGGGGAATACTGAGTAGCGACCAATTGAAT   122
RAD14       AGGAAATTAGCAATAGAACGGTTAAGAAAAAGGGGAATACTGAGTAGCGACCAATTGAAT   180
32RADRp     ------------------------------------------------------------   0


32RADFp     CGAATAGAAAGTAGGAATGAACCTTTAAAAACCCGGCCTCTCGCAGTTACTAGTGGCAGC   182
RAD14       CGAATAGAAAGTAGGAATGAACCTTTAAAAACCCGGCCTCTCGCAGTTACTAGTGGCAGC   240
32RADRp     ------------------------------------------------------------   0


32RADFp     AATCGGGATGATAATGCAGCAGCCGCAGTACATGTGCCAAATCATAATGGACAACCGTCT   242
RAD14       AATCGGGATGATAATGCAGCAGCCGCAGTACATGTGCCAAATCATAATGGACAACCGTCT   300
32RADRp     --TCGGGATGATAATGCAGCAGCCGCAGTACATGTGCCAAATCATAATGGACAACCGTCT   58
              **********************************************************

32RADFp     GCGCTTGCTAACACTAACACTAACACTACTTCACTTTATGGTAGCGGAGTAGTTGATGGA   302
RAD14       GCGCTTGCTAACACTAACACTAACACTACTTCACTTTATGGTAGCGGAGTAGTTGATGGA   360
32RADRp     GCGCTTGCTAACACTAACACTAACACTACTTCACTTTATGGTAGCGGAGTAGTTGATGGA   118
            ************************************************************

32RADFp     AGTAAAAGGGATGCGTCGGTACTCGACAAAAGGCCAACGGATAGAATCAGACCTAGCATA   362
RAD14       AGTAAAAGGGATGCGTCGGTACTCGACAAAAGGCCAACGGATAGAATCAGACCTAGCATA   420
32RADRp     AGTAAAAGGGATGCGTCGGTACTCGACAAAAGGCCAACGGATAGAATCAGACCTAGCATA   178
            ************************************************************

32RADFp     AGGAAACAAGATTACATTGAGTACGATTTTGCCACCATGCAGAACTTGAATGGTGGTTAT   422
RAD14       AGGAAACAAGATTACATTGAGTACGATTTTGCCACCATGCAGAACTTGAATGGTGGTTAT   480
32RADRp     AGGAAACAAGATTACATTGAGTACGATTTTGCCACCATGCAGAACTTGAATGGTGGTTAT   238
            ************************************************************

32RADFp     ATCAACCCTAAGGACAAGCTTCCAAATTCTGACTTTACCGATGACCAAGAATTTGAATCT   482
RAD14       ATCAACCCTAAGGACAAGCTTCCAAATTCTGACTTTACCGATGACCAAGAATTTGAATCT   540
32RADRp     ATCAACCCTAAGGACAAGCTTCCAAATTCTGACTTTACCGATGACCAAGAATTTGAATCT   298
            ************************************************************

32RADFp     GAGTTTGGATCTAAAAAGCAGAAGACACTACAGGACTGGAAAAAGGAACAACTTGAACGG   542
RAD14       GAGTTTGGATCTAAAAAGCAGAAGACACTACAGGACTGGAAAAAGGAACAACTTGAACGG   600
32RADRp     GAGTTTGGATCTAAAAAGCAGAAGACACTACAGGACTGGAAAAAGGAACAACTTGAACGG   358
            ************************************************************

32RADFp     AAAATGCTGTACGAAAATGCACCTCCTCCAGAGCATATTTCAAAGGCGCCGAAATGTATT   602
RAD14       AAAATGCTGTACGAAAATGCACCTCCTCCAGAGCATATTTCAAAGGCGCCGAAATGTATT   660
32RADRp     AAAATGCTGTACGAAAATGCACCTCCTCCAGAGCATATTTCAAAGGCGCCGAAATGTATT   418
            ************************************************************
32RADFp     GAATGTCATATTAATATTGAGATGGATCCTGTGCTACATGATGTGTTCAAGTTACAAGTT   662
RAD14       GAATGTCATATTAATATTGAGATGGATCCTGTGCTACATGATGTGTTCAAGTTACAAGTT   720
32RADRp     GAATGTCATATTAATATTGAGATGGATCCTGTGCTACATGATGTGTTCAAGTTACAAGTT   478
            ************************************************************

32RADFp     TGTAAACAGTGTTCTAAGGAGCATCCAGAAAAGTATGCACTACTGACGAATACAGAATGT   722
RAD14       TGTAAACAGTGTTCTAAGGAGCATCCAGAAAAGTATGCACTACTGACGAAAACAGAATGT   780
```

```
32RADRp       TGTAAACAGTGTTCTAAGGAGCATCCAGAAAAGTATGCACTACTGACGAAAACAGAATGT   538
              ************************************************** *********

32RADFp       AAGGAAGATTACTTTTTAACAGACCCCGAATTGAATGATGAGGATCTCTTTCATAGACTA   782
RAD14         AAGGAAGATTACTTTTTAACAGACCCCGAATTGAATGATGAGGATCTCTTTCATAGACTA   840
32RADRp       AAGGAAGATTACTTTTTAACAGACCCCGAATTGAATGATGAGGATCTCTTTCATAGACTA   598
              ************************************************************

32RADFp       GAAAAGCCGAACCCTCATTCGGGGACATTTGCAAGAATGCAACTATTTGTTAGATGTGAA   842
RAD14         GAAAAGCCGAACCCTCATTCGGGGACATTTGCAAGAATGCAACTATTTGTTAGATGTGAA   900
32RADRp       GAAAAGCCGAACCCTCATTCGGGGACATTTGCAAGAATGCAACTATTTGTTAGATGTGAA   658
              ************************************************************

32RADFp       GTGGAAGCCTTTGCGTTC------------------------------------------   860
RAD14         GTGGAAGCCTTTGCGTTCAAGAAATGGGGTGGGGAAGAAGGTTTAGATGAGGAATGGCAA   960
32RADRp       GTGGAAGCCTTTGCGTTCAAGAAATGGGGTGGAGAAGAAGGTTTAGATGAGGAATGGCAA   718
              ******************

32RADFp       ------------------------------------------------------------   860
RAD14         CGTCGTGAAGAAGGAAAGGCTCACAGAAGGGAGAAAAAATACGAAAAGAAAATCAAGGAA   1020
32RADRp       CGTCGTGAAGAAGGAAAGGCTCACAGAAGGGAGAAAAAATACGAAAAGAAAATCAAGGAA   778

32RADFp       ------------------------------------------------------------   860
RAD14         ATGCGACTGAAAACAAGAGCTCAAGAATATACTAATAGATTAAGGGAAAAGAAGCATGGG   1080
32RADRp       ATGCGACTGAAAACAAGAGCTCAAGAATATACTAATAGATTAAGAGAAAAGAAGCATGGG   838

32RADFp       ------------------------------------------------------------   860
RAD14         AAAGCCCATATTCATCATTTTAGTGATCCAGTTGATGGAGGTATTGATGAAGACGGTTAT   1140
32RADRp       AAAGCCCATATTCATCATTTTAGTGATCCAGTTGATGGAGGTATTGATGAAGACGGT---   895

32RADFp       ------------------------------------------------------------   860
RAD14         CAAATTCAAAGAAGAAGATGTACAGACTGCGGGCTAGAAACTGAAGAAATTGACATTTAA   1200
32RADRp       ------------------------------------------------------------   895
```

SOD1

```
32SOD1Fp      ----------------------------------ACTTAGCTGGTGACTCTGGTGTGTGG   26
SOD1          ---ATGGTTCAAGCAGTCGCAGTGTTAAAGGGTGATGCCGGTGTCTCTGGTGTTGTCAAG   57
32SOD1Rp      TTAATGTTCAAAGGTGTCGCAGTGTTAAAGGGTGATGCCGGTGTCTCTGGTGTTGTCAAG   60
                                                *    * **       ** ***   *

32SOD1Fp      GTTCTAGAGGTTTCCAAAACAGAGCCAACCACTGTCTCTTACAAAATCGCTGGTAACAGT   86
SOD1          TTCGAACAGGCTTCCGAATCCGAGCCAACCACTGTCTCTTACGAGATCGCTGGTAACAGT   117
32SOD1Rp      TTCGAACAGGCTTCCGAATCCGAGCCAACCACTGTCTCTTACGAGATCGCTGGTAACAGT   120
               *   * *** **** ** * ********************* * ***************

32SOD1Fp      CCTAACGCAAAACGGGGGTTCCACATTCATGATTTTGGAAATGCCACCAAGGGTTGTGTC   146
SOD1          CCTAACGCAGAACGTGGGTTCCACATTCATGAGTTTGGAGATGCCACCAATGGTTGTGTC   177
32SOD1Rp      CCTAACGCAGAACGTGGGTTCCACATTCATGAGTTTGGAGATGCCACCAATGGTTGTGTC   180
              ********* **** ***************** ****** ********** *********

32SOD1Fp      TCTGCTGGTCCTCACTTCAATCCTTTCAAAAAAACACATGGGGCTCCAACTGACAAATTC   206
SOD1          TCTGCTGGTCCTCACTTCAATCCTTTCAAGAAGACACATGGTGCTCCAACTGACGAAGTC   237
32SOD1Rp      TCTGCTGGTCCTCACTTCAATCCTTTCAAGAAGACACATGGTGCTCCAACTGACGAAGTC   240
              ***************************** ** ******** ************ ** **

32SOD1Fp      AAACTTGTCGGTGACATGGGTAACGTAAAAACGGACAAAAATGGTGTGGCCAAGGGCTCC   266
SOD1          AGACATGTCGGTGACATGGGTAACGTAAAGACGGACGAAAATGGTGTGGCCAAGGGCTCC   297
32SOD1Rp      AGACATGTCGGTGACATGGGTAACGTAAAGACGGACGAAAATGGTGTGGCCAAGGGCTCC   300
              * ** ************************ ****** ***********************

32SOD1Fp      TTCAAGGACTCTTTGATCAAGCTTATCGGTCCTACCTCCGTTGTAGGCAAAACCGTCTTT   326
SOD1          TTCAAGGACTCTTTGATCAAGCTTATCGGTCCTACCTCCGTTGTAGGCAGAAGCGTCGTT   357
```

```
32SOD1Rp      TTCAAGGACTCTTTGATCAAGCTTATCGGTCCTACCTCCGTTGTAGGCAGAAGCGTCGTT   360
              ************************************************* ** **** **

32SOD1Fp      ATCCCCCCCGGCCAAAATGACTTAGGTAAGGGTGACACTGAAAAATCTTTGAAAACTGGT   386
SOD1          ATCCACGCCGGCCAAGATGACTTAGGTAAGGGTGACACTGAAGAATCTTTGAAGACTGGT   417
32SOD1Rp      ATCCACGCCGGCCAAGATGACTTAGGTAAGGGTGACACTCAGTAATCAGGAAGACCGGTA   420
              **** * ******** *********************** *  ****    *   * *

32SOD1Fp      AATGCCGGTCCAAAACCACCCTGTGGTGTCATTGGTCTAACCAACTAAAA   436
SOD1          AATGCCGGTCCAAGACCAGCCTGTGGTGTCATTGGTCTAACCAACTAA--   465
32SOD1Rp      ATG-CCGTCCATGATCTGT-------------------------------   438
              *   ***  *     *
```

COX2

```
32COXFp       ------------------------------------------------AAATTCTTATGA   12
COX           -----------------ATGTTAGATTTATTAAGATTACAATTAACAACATTCATTATGA   43
32COXRp       TTTTGTTTTTTTTTTTAATGTTAGATTTATTAAGATTACAATTAACAACATTCATTATGA   60
                                                              .*:* .******

32COXFp       ATGATGTACCAACACCTTATGCATGTTATTTTCAGGATTCAGCAACACCAAATCAAGAAG   72
COX           ATGATGTACCAACACCTTATGCATGTTATTTTCAGGATTCAGCAACACCAAATCAAGAAG   103
32COXRp       ATGATGTACCAACACCTTATGCATGTTATTTTCAGGATTCAGCAACACCAAATCAAGAAG   120
              ************************************************************

32COXFp       GTATTTTAGAATTACATGATAATATTATGTTTTATTTATTAGTTATTTTAGGTTTAGTAT   132
COX           GTATTTTAGAATTACATGATAATATTATGTTTTATTTATTAGTTATTTTAGGTTTAGTAT   163
32COXRp       GTATTTTAGAATTACATGATAATATTATGTTTTATTTATTAGTTATCTTAGGTTTAGTAT   180
              ********************************************** *************

32COXFp       CTTGAATGTTATATACAATTGTTATAACATATTCAAAAAATCCTATTGCATATAAATATA   192
COX           CTTGAATGTTATATACAATTGTTATAACATATTCAAAAAATCCTATTGCATATAAATATA   223
32COXRp       CTTGAATGTTATATACAATTGTTATAACATATTCAAAAAATCCTATTGCATATAAATATA   240
              ************************************************************

32COXFp       TTAAACATGGACAAACTATTGAAGTTATTTGAACAATTTTTCCAGCTGTAATTTTATTAA   252
COX           TTAAACATGGACAAACTATTGAAGTTATTTGAACAATTTTTCCAGCTGTAATTTTATTAA   283
32COXRp       TTAAACATGGACAAACTATTGAAGTTATTTGAACAATTTTTCCAGCTGTAATTTTATTAA   300
              ************************************************************

32COXFp       TTATTGCTTTTCCTTCATTTATTTTATTATATTTATGTGATGAAGTTATTTCACCAGCTA   312
COX           TTATTGCTTTTCCTTCATTTATTTTATTATATTTATGTGATGAAGTTATTTCACCAGCTA   343
32COXRp       TTATTGCTTTTCCTTCATTTATTTTATTATATTTATGTGATGAAGTTATTTCACCAGCTA   360
              ************************************************************

32COXFp       TAACTATTAAAGCTATTGGATATCAATGATATTGAAAATATGAATATTCACATTTTATTA   372
COX           TAACTATTAAAGCTATTGGATATCAATGATATTGAAAATATGAATATTCAGATTTTATTA   403
32COXRp       TAACTATTAAAGCTATTGGATATCAATGATATTGAAAATATGAATATTCAGATTTTATTA   420
              ************************************************** *********

32COXFp       ATGATAGTGGTGAAACTGTTGAATTTGAATCATATGTTATTCCTGATGAATTATTAGAAG   432
COX           ATGATAGTGGTGAAACTGTTGAATTTGAATCATATGTTATTCCTGATGAATTATTAGAAG   463
32COXRp       ATGATAGTGGTGAAACTGTTGAATTTGAATCATATGTTATTCCTGATGAATTATTAGAAG   480
              ************************************************************

32COXFp       AACGTCAATTAAGATTATTACATACGGATACTTCGATAGTTGTACCTGTATATACTCTTA   492
COX           AAGGTCAATTAAGATTATTAGATACTGATACTTCTATAGTTGTACCTGTAGATACACATA   523
32COXRp       AAGGTCAATTAAGATTATTAGATACTGATACTTCTATAGTTGTACCTGTAGATACACATA   540
              ** ***************** **** ******** *************** ****:*:**

32COXFp       TTGTATTCGTTGTAACAGCTGCTGAGGTTATTCGAGAATTTGCTATTCCTTGTTTATGTA   552
COX           TTAGATTCGTTGTAACAGCTGCTGATGTTATTCATGATTTTGCTATTCCAAGTTTAGGTA   583
32COXRp       TTAGATTCGTTGTAACAGCTGCTGATGTTATTCATGATTTTGCTATTCCAAGTTTAGGTA   600
              **. ********************* *******.:**:***********::***** ***

32COXFp       TTTCAGTAGACGCTACTCCAGGTAAATTAAATCTAGTTTCTGCTTTTATTCTGAGATTCT   612
COX           TTAAAGTTGATGCTACTCCTGGTAGATTAAATCAAGTTTCTGCTTTAATTCAAAGAGAAG   643
```

```
32COXRp         TTAAAGTTGATGCTACTCCTGGTAGATTAAATCAAGTTTCTGCTTTAATTCAAAGAGAAG    660
                **:.***:** ********:****.********:************:****:.*** :.

32COXFp         GTGTCACCTTCGTGACTTGTTGTTACTGAAGCCTGACAAGAACATGCATATATGCGTTGG    672
COX             GTGTCTTCTATGGAGCATGTTCTGAGTTGTGTGGGA-CAGGTCATGCAAATATGCCAA-T    701
32COXRp         GTGTCTTCTATGGAGCATGTTCTGAGTTGTGTGGGA-CAGGTCATGCAAATATGCCCAAT    719
                *****: **: *  ..*:**** * * *  .:*    ** .**.:******:******  :

32COXFp         TAACATCATTCACAGTATCATGTTCCTAAATTGTTGTCTTGATTAATCGAACCATAAAAA    732
COX             TAAGATCGA-AGCAGTATCATTA-CCTAAATTTTGGAATGATTAAATGAACAATAA---    756
32COXRp         TAAGATCGAATGCAGTTTCCATA-CAATT-------------------------------    747
                *** ***.:  .****:**.: : *.:::

32COXFp         AAAAAAAAAAA  743
COX             -----------  756
32COXRp         -----------  747
```

HSP33

RAD14

```
33RADRp         ------------------------------------------------------------    0
RAD14           ATGACTCCCGAACAAAAGGCCAAACTAGTATGTGTGAAAATGATTCTGTGTTTGTATTTT    60
33RADFp         ------------------------------------------------------------    0


33RADRp         ------------------------------------------------------------    0
RAD14           AACCGTGGGTTTCTTTTTACTAACAATTTAACGATGAGATGAGCTGTGCAGGAGGCTAAC    120
33RADFp         --CCGTGGGTTTCTTTTTACTAACAATTTAACGATGAGATGAGCTGTGCAGGAGGCTAAC    58


33RADRp         ------------------------------------------------------------    0
RAD14           AGGAAATTAGCAATAGAACGGTTAAGAAAAAGGGGAATACTGAGTAGCGACCAATTGAAT    180
33RADFp         AGGAAATTAGCAATAGAACGGTTAAGAAAAAGGGGAATACTGAGTAGCGACCAATTGAAT    118


33RADRp         ------------------------------------------------------------    0
RAD14           CGAATAGAAAGTAGGAATGAACCTTTAAAAACCCGGCCTCTCGCAGTTACTAGTGGCAGC    240
33RADFp         CGAATAGAAAGTAGGAATGAACCTTTAAAAACCCGGCCTCTCGCAGTTACTAGTGGCAGC    178


33RADRp         ------------------------------------------------------------    0
RAD14           AATCGGGATGATAATGCAGCAGCCGCAGTACATGTGCCAAATCATAATGGACAACCGTCT    300
33RADFp         AATCGGGATGATAATGCAGCAGCCGCAGTACATGTGCCAAATCATAATGGACAACCGTCT    238


33RADRp         --------------------------TACTTCACTTTATGGTAGCGGAGT-AGTGATGGA    33
RAD14           GCGCTTGCTAACACTAACACTAACACTACTTCACTTTATGGTAGCGGAGTAGTTGATGGA    360
33RADFp         GCGCTTGCTAACACTAACACTAACACTACTTCACTTTATGGTAGCGGAGTAGTTGATGGA    298
                                          ************************   *******

33RADRp         AGTAAAAGGGATGTGTCGGTACTCGACAAAAGGCCAACGGATAGAATCAGACCTAGCATA    93
RAD14           AGTAAAAGGGATGCGTCGGTACTCGACAAAAGGCCAACGGATAGAATCAGACCTAGCATA    420
33RADFp         AGTAAAAGGGATGCGTCGGTACTCGACAAAAGGCCAACGGATAGAATCAGACCTAGCATA    358
                ************* **********************************************

33RADRp         AGGAAACAAGATTACATTGAGTACGATTTTGCCACCATGCAGAACTTGAATGGTGGTTAT    153
RAD14           AGGAAACAAGATTACATTGAGTACGATTTTGCCACCATGCAGAACTTGAATGGTGGTTAT    480
33RADFp         AGGAAACAAGATTACATTGAGTACGATTTTGCCACCATGCAGAACTTGAATGGTGGTTAT    418
                ************************************************************

33RADRp         ATCAACCCTAAGGACAAGCTTCCAAATTCTGACTTTACCGATGACCAAGAATTTGAATCT    213
RAD14           ATCAACCCTAAGGACAAGCTTCCAAATTCTGACTTTACCGATGACCAAGAATTTGAATCT    540
33RADFp         ATCAACCCTAAGGACAAGCTTCCAAATTCTGACTTTACCGATGACCAAGAATTTGAATCT    478
                ************************************************************

33RADRp         GAGTTTGGATCTAAAAAGCAGAAGACACTACAGGACTGGAAAAAGGAACAACTTGAACGG    273
```

```
RAD14          GAGTTTGGATCTAAAAAGCAGAAGACACTACAGGACTGGAAAAAGGAACAACTTGAACGG   600
33RADFp        GAGTTTGGATCTAAAAAGCAGAAGACACTACAGGACTGGAAAAAGGAACAACTTGAACGG   538
               ************************************************************

33RADRp        AAAATGCTGTACGAAAATGCACCTCCTCCAGAGCATATTTCAAAGGCGCCGAAATGTATT   333
RAD14          AAAATGCTGTACGAAAATGCACCTCCTCCAGAGCATATTTCAAAGGCGCCGAAATGTATT   660
33RADFp        AAAATGCTGTACGAAAATGCACCTCCTCCAGAGCATATTTCAAAGGCGCCGAAATGTATT   598
               ************************************************************

33RADRp        GAATGTCATATTAATATTGAGATGGATCCTGTGCTACATGATGTGTTCAAGTTACAAGTT   393
RAD14          GAATGTCATATTAATATTGAGATGGATCCTGTGCTACATGATGTGTTCAAGTTACAAGTT   720
33RADFp        GAATGTCATATTAATATTGAGATGGATCCTGTGCTACATGATGTGTTCAAGTTACAAGTT   658
               ************************************************************

33RADRp        TGTAAACAGTGTTCTAAGGAGCATCCAGAAAAGTATGCACTACTGACGAAAACAGAATGT   453
RAD14          TGTAAACAGTGTTCTAAGGAGCATCCAGAAAAGTATGCACTACTGACGAAAACAGAATGT   780
33RADFp        TGTAAACAGTGTTCTAAGGAGCATCCAGAAAAGTATGCACTACTGACGAAAACAGAATGT   718
               ************************************************************

33RADRp        AAGGAAGATTACTTTTTAACAGACCCCGAATTGAATGATGAGGATCTCTTTCATAGACTA   513
RAD14          AAGGAAGATTACTTTTTAACAGACCCCGAATTGAATGATGAGGATCTCTTTCATAGACTA   840
33RADFp        AAGGAAGATTACTTTTTAACAGACCCCGAATTGAATGATGAGGATCTCTTTCATAGACTA   778
               ************************************************************

33RADRp        GAAAAGCCGAACCCTCATTCGGGGACATTTGCAAGAATGCAACTATTTGTTAGATGTGAA   573
RAD14          GAAAAGCCGAACCCTCATTCGGGGACATTTGCAAGAATGCAACTATTTGTTAGATGTGAA   900
33RADFp        GAAAAGCCGAACCCTCATTCGGGGACATTTGCAAGAATGCAACTATTTGTTAGATGTGAA   838
               ************************************************************

33RADRp        GTGGAAGCCTTTGCGTTCAAGAAATGGGGTGGAGAAGAAGGTTTAGATGAGGAATGGCAA   633
RAD14          GTGGAAGCCTTTGCGTTCAAGAAATGGGGTGGGGAAGAAGGTTTAGATGAGGAATGGCAA   960
33RADFp        ------------------------------------------------------------   838

33RADRp        CGTCGTGAAGAAGGAAAGGCTCACAGAAGGGAGAAAAAATACGAAAAGAAAATCAAGGAA   693
RAD14          CGTCGTGAAGAAGGAAAGGCTCACAGAAGGGAGAAAAAATACGAAAAGAAAATCAAGGAA   1020
33RADFp        ------------------------------------------------------------   838

33RADRp        ATGCGACTGAAAACAAGAGCTCAAGAATATACTAATAGATTAAGAGAAAAGAAGCATGGG   753
RAD14          ATGCGACTGAAAACAAGAGCTCAAGAATATACTAATAGATTAAGGGAAAAGAAGCATGGG   1080
33RADFp        ------------------------------------------------------------   838

33RADRp        AAAGCCCATATTCATCATTTTAGTGATCCAGTTGATGGAGGTATTGATGAAGACGGTTAT   813
RAD14          AAAGCCCATATTCATCATTTTAGTGATCCAGTTGATGGAGGTATTGATGAAGACGGTTAT   1140
33RADfp        ------------------------------------------------------------   838

33RADRp        CA----------------------------------------------------------   815
RAD14          CAAATTCAAAGAAGAAGATGTACAGACTGCGGGCTAGAAACTGAAGAAATTGACATTTAA   1200
33RADFp        ------------------------------------------------------------   838
```

SOD1

```
33SOD1Fp       ---------------------------------ACGTCCCAGGTGCCTCTGGTGTGTGGG   27
SOD1           ---ATGGTTCAAGCAGTCGCAGTGTTAAAGGGTGATGCCGGTGTCTCTGGTGTTGTCAAG   57
33SOD1Rp       TTAAGGTTTAAAACAGTCGCAGTGTTAAAGGGTGATGCCGGTGTCTCTGGTGTTGTCAAG   60
                                                     **   **  **   **      *

33SOD1Fp       TCCTTGAGCAAATCCAAAAAAGAGCCAACCACTGTCTCTTACGAGATCGCTGGTAACAGT   87
SOD1           TTCGAACAGGCTTCCGAATCCGAGCCAACCACTGTCTCTTACGAGATCGCTGGTAACAGT   117
33SOD1Rp       TTCGAACAGGCTTCCGAATCCGAGCCAACCACTGTCTCTTACGAGATCGCTGGTAACAGT   120
               * *         *** **   ***************************************

33SOD1Fp       CCTAACGCAAAACGGGGGTTCCACATTCATGATTTTGGAAATGCCACCAAGGGTTGTGTC   147
SOD1           CCTAACGCAGAACGTGGGTTCCACATTCATGAGTTTGGAGATGCCACCAATGGTTGTGTC   177
```

```
33SOD1Rp       CCTAACGCAGAACGTGGGTTCCACATTCATGAGTTTGGAGATGCCACCAATGGTTGTGTC   180
               ********* **** ***************** ****** ********** *********

33SOD1Fp       TCTGCTGGTCCTCACTTCAATCCTTTCAAGAAAACACATGGGGCTCCAACTGACAAATTC   207
SOD1           TCTGCTGGTCCTCACTTCAATCCTTTCAAGAAGACACATGGTGCTCCAACTGACGAAGTC   237
33SOD1Rp       TCTGCTGGTCCTCACTTCAATCCTTTCAAGAAGACACATGGTGCTCCAACTGACGAAGTC   240
               ******************************** ******** ************ ** **

33SOD1Fp       AAACATGTCGGTGACATGGGTAACGTAAAGACGGACAAAAATGGTGTGGCCAAGGGCTCC   267
SOD1           AGACATGTCGGTGACATGGGTAACGTAAAGACGGACGAAAATGGTGTGGCCAAGGGCTCC   297
33SOD1Rp       AGACATGTCGGTGACATGGGTAACGTAAAGACGGACGAAAATGGTGTGGCCAAGGGCTCC   300
               * ********************************** ***********************

33SOD1Fp       TTCAAGGACTCTTTGATCAAGCTTATCGGTCCTACCTCCGTTGTAGGCAAAAGCGTCTTT   327
SOD1           TTCAAGGACTCTTTGATCAAGCTTATCGGTCCTACCTCCGTTGTAGGCAGAAGCGTCGTT   357
33SOD1Rp       TTCAAGGACTCTTTGATCAAGCTTATCGGTCCTACCTCCGTTGTAGGCAGAAGCGTCGTT   360
               ************************************************* ******* **

33SOD1Fp       ATCCCCCCCGGCCAAAATGACTTAGGTAAGGGTGACACTGAAAAATCTTTGAAAACTGGT   387
SOD1           ATCCACGCCGGCCAAGATGACTTAGGTAAGGGTGACACTGAAGAATCTTTGAAGACTGGT   417
33SOD1Rp       ATCCACGCCGGCCAAGATGACTTAGGTAATAGTGTGTATCTACAGCAGTACATCAGGAAG   420
               **** * ******** *************  ***    *  * *    *  *  *

33SOD1Fp       AATGCCGGTCCAAGACCAGCCTGTGGTGTCTATGGTCAAACAAAAAAAAAA   438
SOD1           AATGCCGGTCCAAGACCAGCCTGTGGTGTCATTGGTCTAACCAACTAA---   465
33SOD1Rp       AACGGTGATAAAGTGTGATGACTGC--------------------------   445
               ** *  * *  *     *
```

COX2

```
33CoxFp      ----------------------------------------------------------AC   2
Cox2         --------ATGTTAGATTTATTAAGATTACAATTAACAACATTCATTATGAATGATGTAC   52
33CoxRp      TTTTTATTATGTTAGATTTATTAAGATTACAATTAACAACATTCATTATGAATGATGTAC   60
                                                                       **

33CoxFp      AACACCTTATGCATGTTTATTTTCAGGATTCAGCAACACCAAATCAAGAAGGTATTTTAG   62
Cox2         CAACACCTTATGCATGTTATTTTCAGGATTCAGCAACACCAAATCAAGAAGGTATTTTAG   112
33CoxRp      CAACACCTTATGCATGTTATTTTCAGGATTCAGCAACACCAAATCAAGAAGGTATTTTAG   120
              *   * *        ********************************************

33CoxFp      AATTACATGATAATATTATGTTTTATTTATTAGTTATTTTAGGTTTAGTATCTTGAATGT   122
Cox2         AATTACATGATAATATTATGTTTTATTTATTAGTTATTTTAGGTTTAGTATCTTGAATGT   172
33CoxRp      AATTACATGATAATATTATGTTTTATTTATTAGTTATTTTAGGTTTAGTATCTTGAATGT   180
             ************************************************************

33CoxFp      TATATACAATTGTTATAACATATTCAAAAAATCCTATTGCATATAAATATATTAAACATG   182
Cox2         TATATACAATTGTTATAACATATTCAAAAAATCCTATTGCATATAAATATATTAAACATG   232
33CoxRp      TATATACAATTGTTATAACATATTCAAAAAATCCTATTGCATATAAATATATTAAACATG   240
             ************************************************************

33CoxFp      GACAAACTATTGAAGTTATTTGAACAATTTTTCCAGCTGTAATTTTATTAATTATTGCTT   242
Cox2         GACAAACTATTGAAGTTATTTGAACAATTTTTCCAGCTGTAATTTTATTAATTATTGCTT   292
33CoxRp      GACAAACTATTGAAGTTATTTGAACAATTTTTCCAGCTGTAATTTTATTAATTATTGCTT   300
             ************************************************************

33CoxFp      TTCCTTCATTTATTTTATTATATTTATGTGATGAAGTTATTTCACCAGCTATAACTATTA   302
Cox2         TTCCTTCATTTATTTTATTATATTTATGTGATGAAGTTATTTCACCAGCTATAACTATTA   352
33CoxRp      TTCCTTCATTTATTTTATTATATTTATGTGATGAAGTTATTTCACCAGCTATAACTATTA   360
             ************************************************************

33CoxFp      AAGCTATTGGATATCAATGATATTGAAAATATGAATATTCAGATTTTATTAATGATAGTG   362
Cox2         AAGCTATTGGATATCAATGATATTGAAAATATGAATATTCAGATTTTATTAATGATAGTG   412
33CoxRp      AAGCTATTGGATATCAATGATATTGAAAATATGAATATTCAGATTTTATTAATGATAGTG   420
             ************************************************************

33CoxFp      GTGAAACTGTTGAATTTGAATCATATGTTATTCCTGATGAATTATTAGAAGAAGGTCAAT   422
Cox2         GTGAAACTGTTGAATTTGAATCATATGTTATTCCTGATGAATTATTAGAAGAAGGTCAAT   472
```

```
33CoxRp       GTGAAACTGTTGAATTTGAATCATATGTTATTCCTGATGAATTATTAGAAGAAGGTCAAT    480
              ************************************************************

33CoxFp       TAAGATTATTAGATACTGATACTTCTATAGTTGTACCTGTAGATACACATATTAGATTCG    482
Cox2          TAAGATTATTAGATACTGATACTTCTATAGTTGTACCTGTAGATACACATATTAGATTCG    532
33CoxRp       TAAGATTATTAGATACTGATACTTCTATAGTTGTACCTGTAGATACACATATTAGATTCG    540
              ************************************************************

33CoxFp       TTGTAACAGCTGCTGATGTTATTCATGATTTTGCTATTCCAAGTTTAGGTATTAAAGTTG    542
Cox2          TTGTAACAGCTGCTGATGTTATTCATGATTTTGCTATTCCAAGTTTAGGTATTAAAGTTG    592
33CoxRp       TTGTAACAGCTGCTGATGTTATTCATGATTTTGCTATTCCAAGTTTAGGTATTAAAGTTG    600
              ************************************************************

33CoxFp       ATGCTACTCCTGGTAGATTAAATCAAGTTTCTGCTTTAATTCAGAGAGAAGGTGTCTTCT    602
Cox2          ATGCTACTCCTGGTAGATTAAATCAAGTTTCTGCTTTAATTCAAAGAGAAGGTGTCTTCT    652
33CoxRp       ATGCTACTCCTGGTAGATTAAATCAAGTTTCTGCTTTAATTCAAAGAGAAGGTGTCTTCT    660
              ****************************************** *****************

33CoxFp       ATGGAGCATGTTCTGAGTTGTGTGGGACAGGTCATGCAAATATGCCAATTAAGATCGAAG    662
Cox2          ATGGAGCATGTTCTGAGTTGTGTGGGACAGGTCATGCAAATATGCCAATTAAGATCGAAG    712
33CoxRp       ATGGAGCATGTTCTGAGTTGTGTGGGACAGGTCATGCAAAATATGCCCAATTAAGATA--    718
              ****************************************     *       *   *

33CoxFp       CAGTATCATTACCTAAATTGTTGGAATGATTAATGAACATAAAAAAAAAA    712
Cox2          CAGTATCATTACCTAAATTTTTGGAATGATTAAATGAACAATAA------    756
33CoxRp       --------------------------------------------------    718
```

HSP34

RAD14

```
34RADRp       ------------------------------------------------------------    0
RAD14         ATGACTCCCGAACAAAAGGCCAAACTAGTATGTGTGAAAATGATTCTGTGTTTGTATTTT    60
34RADFp       --------------------------------------------------------TTTT    4


34RADRp       ------------------------------------------------------------    0
RAD14         AACCGTGGGTTTCTTTTTACTAACAATTTAACGATGAGATGAGCTGTGCAGGAGGCTAAC    120
34RADFp       AACCGTGGGTTTCTTTTTACTAACAATTTAACGATGAGATGAGCTGTGCAGGAGGCTAAC    64


34RADRp       ------------------------------------------------------------    0
RAD14         AGGAAATTAGCAATAGAACGGTTAAGAAAAAGGGGAATACTGAGTAGCGACCAATTGAAT    180
34RADFp       AGGAAATTAGCAATAGAACGGTTAAGAAAAAGGGGAATACTGAGTAGCGACCAATTGAAT    124


34RADRp       ------------------------------------------------------------    0
RAD14         CGAATAGAAAGTAGGAATGAACCTTTAAAAACCCGGCCTCTCGCAGTTACTAGTGGCAGC    240
34RADFp       CGAATAGAAAGTAGGAATGAACCTTTAAAAACCCGGCCTCTCGCAGTTACTAGTGGCAGC    184


34RADRp       ------------------------------------------------------------    0
RAD14         AATCGGGATGATAATGCAGCAGCCGCAGTACATGTGCCAAATCATAATGGACAACCGTCT    300
34RADFp       AATCGGGATGATAATGCAGCAGCCGCAGTACATGTGCCAAATCATAATGGACAACCGTCT    244


34RADRp       -----------------------------------TTATGGTAGCGGAGTAGTTGATGGA    25
RAD14         GCGCTTGCTAACACTAACACTAACACTACTTCACTTTATGGTAGCGGAGTAGTTGATGGA    360
34RADFp       GCGCTTGCTAACACTAACACTAACACTACTTCACTTTATGGTAGCGGAGTAGTTGATGGA    304
                                                 *************************

34RADRp       AGTAAAAGGGATGCGTCGGTACTCGACAAAAGGCCAACGGATAGAATCAGACCTAGCATA    85
RAD14         AGTAAAAGGGATGCGTCGGTACTCGACAAAAGGCCAACGGATAGAATCAGACCTAGCATA    420
34RADFp       AGTAAAAGGGATGCGTCGGTACTCGACAAAAGGCCAACGGATAGAATCAGACCTAGCATA    364
              ************************************************************

34RADRp       AGGAAACAAGATTACATTGAGTACGATTTTGCCACCATGCAGAACTTGAATGGTGGTTAT    145
```

```
RAD14        AGGAAACAAGATTACATTGAGTACGATTTTGCCACCATGCAGAACTTGAATGGTGGTTAT   480
34RADFp      AGGAAACAAGATTACATTGAGTACGATTTTGCCACCATGCAGAACTTGAATGGTGGTTAT   424
             ************************************************************

34RADRp      ATCAACCCTAAGGACAAGCTTCCAAATTCTGACTTTACCGATGACCAAGAATTTGAATCT   205
RAD14        ATCAACCCTAAGGACAAGCTTCCAAATTCTGACTTTACCGATGACCAAGAATTTGAATCT   540
34RADFp      ATCAACCCTAAGGACAAGCTTCCAAATTCTGACTTTACCGATGACCAAGAATTTGAATCT   484
             ************************************************************

34RADRp      GAGTTTGGATCTAAAAAGCAGAAGACACTACAGGACTGGAAAAAGGAACAACTTGAACGG   265
RAD14        GAGTTTGGATCTAAAAAGCAGAAGACACTACAGGACTGGAAAAAGGAACAACTTGAACGG   600
34RADFp      GAGTTTGGATCTAAAAAGCAGAAGACACTACAGGACTGGAAAAAGGAACAACTTGAACGG   544
             ************************************************************

34RADRp      AAAATGCTGTACGAAAATGCACCTCCTCCAGAGCATATTTCAAAGGCGCCGAAATGTATT   325
RAD14        AAAATGCTGTACGAAAATGCACCTCCTCCAGAGCATATTTCAAAGGCGCCGAAATGTATT   660
34RADFp      AAAATGCTGTACGAAAATGCACCTCCTCCAGAGCATATTTCAAAGGCGCCGAAATGTATT   604
             ************************************************************

34RADRp      GAATGTCATATTAATATTGAGATGGATCCTGTGCTACATGATGTGTTCAAGTTACAAGTT   385
RAD14        GAATGTCATATTAATATTGAGATGGATCCTGTGCTACATGATGTGTTCAAGTTACAAGTT   720
34RADFp      GAATGTCATATTAATATTGAGATGGATCCTGTGCTACATGATGTGTTCAAGTTACAAGTT   664
             ************************************************************

34RADRp      TGTAAACAGTGTTCTAAGGAGCATCCAGAAAAGTATGCACTACTGACGAAAACAGAATGT   445
RAD14        TGTAAACAGTGTTCTAAGGAGCATCCAGAAAAGTATGCACTACTGACGAAAACAGAATGT   780
34RADFp      TGTAAACAGTGTTCTAAGGAGCATCCAGAAAAGTATGCACTACTGACGAAAACAGAATGT   724
             ************************************************************

34RADRp      AAGGAAGATTACTTTTTAACAGACCCCGAATTGAATGATGAGGATCTCTTTCATAGACTA   505
RAD14        AAGGAAGATTACTTTTTAACAGACCCCGAATTGAATGATGAGGATCTCTTTCATAGACTA   840
34RADFp      AAGGAAGATTACTTTTTAACAGACCCCGAATTGAATGATGAGGATCTCTTTCATAGACTA   784
             ************************************************************

34RADRp      GAAAA-GCCGAACCCTCATTCGGGGACATTTGCAAGAATGCAACTATTTGTTAGATGTGA   564
RAD14        GAAAA-GCCGAACCCTCATTCGGGGACATTTGCAAGAATGCAACTATTTGTTAGATGTGA   899
34RADFp      GAAAAAGCCGAACCCTCATTCGGGGACATTTGCAAGAATGCAACTATTTGTTAGATGTGA   844
             ***** ******************************************************

34RADRp      AGTGGAAGCCTTTGCGTTCAAGAAATGGGGTGGAGAAGAAGGTTTAGATGAGGAATGGCA   624
RAD14        AGTGGAAGCCTTTGCGTTCAAGAAATGGGGTGGGGAAGAAGGTTTAGATGAGGAATGGCA   959
34RADFp      AGTG--------------------------------------------------------   848
             ****

34RADRp      ACGTCGTGAAGAAGGAAAGGCTCACAGAAGGGAGAAAAAATACGAAAAGAAAATCAAGGA   684
RAD14        ACGTCGTGAAGAAGGAAAGGCTCACAGAAGGGAGAAAAAATACGAAAAGAAAATCAAGGA   1019
34RADFp      ------------------------------------------------------------   848

34RADRp      AATGCGACTGAAAACAAGAGCTCAAGAATATACTAATAGATTAAGAGAAAAGAAGCATGG   744
RAD14        AATGCGACTGAAAACAAGAGCTCAAGAATATACTAATAGATTAAGGGAAAAGAAGCATGG   1079
34RADFp      ------------------------------------------------------------   848

34RADRp      GAAAGCCCATATTCATCATTTTAGTGATCCAGTTGATGGAGGTATTGATGAAGACGGTTA   804
RAD14        GAAAGCCCATATTCATCATTTTAGTGATCCAGTTGATGGAGGTATTGATGAAGACGGTTA   1139
34RADFp      ------------------------------------------------------------   848

34RADRp      TCAAATCAAAGAAGAA--------------------------------------------   820
RAD14        TCAAATTCAAAGAAGAAGATGTACAGACTGCGGGCTAGAAACTGAAGAAATTGACATTTA   1199
34RADFp      ------------------------------------------------------------   848

34RADRp      -   820
RAD14        A   1200
34RADFp      -   848
```

SOD1

```
34SOD1Fp        ------------------------------ACTGGAGCCGGGTGACTCTGGTGTGTGGGT   30
SOD1            --ATGGTTCAAGCAGTCGCAGTGTTAAAGGGTGATGCCGGTGTCTCTGGTGTTGTCAAGT   58
34SOD1Rp        TTAAGGTTCCAAGAGTTGCAGTGTTAAAGGGTGATGCCGGTGTCTCTGGTGTTGTCAAGT   60
                                                     * * **  **   **      **

34SOD1Fp        TCTAGAGGTAATCCAAAACAAAGCCAACCACTGTCTCTTACAAAATCGCTGGTAACAGTC   90
SOD1            TCGAACAGGCTTCCGAATCCGAGCCAACCACTGTCTCTTACGAGATCGCTGGTAACAGTC   118
34SOD1Rp        TCGAACAGGCTTCCGAATCCGAGCCAACCACTGTCTCTTACGAGATCGCTGGTAACAGTC   120
                ** *   *   *** ** *  ******************** * ****************

34SOD1Fp        CTAACGCAAAACGGGGGTTCCACATTCATGATTTTGGAAATGCCACCAAGGGTTGTGTCT   150
SOD1            CTAACGCAGAACGTGGGTTCCACATTCATGAGTTTGGAGATGCCACCAATGGTTGTGTCT   178
34SOD1Rp        CTAACGCAGAACGTGGGTTCCACATTCATGAGTTTGGAGATGCCACCAATGGTTGTGTCT   180
                ******** **** ***************** ****** ********** **********

34SOD1Fp        CTGCTGGTCCTCACTTCAATCCTTTCAAAAAAACACATGGGGCTCCAACTGACAAATTCA   210
SOD1            CTGCTGGTCCTCACTTCAATCCTTTCAAGAAGACACATGGTGCTCCAACTGACGAAGTCA   238
34SOD1Rp        CTGCTGGTCCTCACTTCAATCCTTTCAAGAAGACACATGGTGCTCCAACTGACGAAGTCA   240
                **************************** ** ******** ************ ** ***

34SOD1Fp        AACATGTCGGTGACATGGGTAACGTAAAGACGGACAAAAATGGTGTGGCCAAGGGCTCCT   270
SOD1            GACATGTCGGTGACATGGGTAACGTAAAGACGGACGAAAATGGTGTGGCCAAGGGCTCCT   298
34SOD1Rp        GACATGTCGGTGACATGGGTAACGTAAAGACGGACGAAAATGGTGTGGCCAAGGGCTCCT   300
                 ********************************** ************************

34SOD1Fp        TCAAGGACTCTTTGATCAAGCTTATCGGTCCTACCTCCGTTGTAGGCAAAACCGTCTTTA   330
SOD1            TCAAGGACTCTTTGATCAAGCTTATCGGTCCTACCTCCGTTGTAGGCAGAAGCGTCGTTA   358
34SOD1Rp        TCAAGGACTCTTTGATCAAGCTTATCGGTCCTACCTCCGTTGTAGGCAGAAGCGTCGTTA   360
                ************************************************ ** **** ***

34SOD1Fp        TCCACCCCGGCCAAAATGACTTAGGTAAGGGTGACACTGAAAAATCTTTGA--AAACTGG   388
SOD1            TCCACGCCGGCCAAGATGACTTAGGTAAGGGTGACACTGAAGAATCTTTGA--AGACTGG   416
34SOD1Rp        TCCACGCCGGCCAAGATGACTTAGGTAATAGGTGATCACAGCAGTAATCAGGAAGACTGG   420
                ***** ******** *************  *     *  *  * *  *     * *****

34SOD1Fp        TAATGCCGGTCCAAAACCAGCCTGTGGTGTCATTGGTCTAAAACAACTAAAA  440
SOD1            TAATGCCGGTCCAAGACCAGCCTGTGGTGTCATTGGTCTAACCAACTAA---  465
34SOD1Rp        TGATAACGTCCATGACACTC--------------------------------  4
```

COX2

```
COX             -----------------ATGTTAGATTTATTAAGATTACAATTAACAACATTCATTATGA   43
34COXFp         ------------------------------------------------AAATTCTTATGA   12
34COXRp         TTTTTTTTTTTTTTTTTAAGGTTAGATTTATTAAGATTACAATTAACAACATTCATTATGA   60
                                                                .*:* .******

COX             ATGATGTACCAACACCTTATGCATGTT-ATTTTCAGGATTCAGCAACACCAAATCAAGA-   101
34COXFp         ATGATGTACCAACACCTTATGCATGTTTATTTTCAGGATTCAGCAACACCAAATCAAGGA   72
34COXRp         ATGATGTACCAACACCTTATGCATGTT-ATTTTCAGGATTCAGCAACACCAAATCAAGA-   118
                *************************** ******************************.

COX             AGGTATTTTAGAATTACATGATAATATTATGTTTTATTTATTAGTTATTTTAGGTTTAG-   160
34COXFp         AGGTATTTTACAATTACATGATAATATTATGTTTTATTTATTATTTATTTTAGGTTTAGT   132
34COXRp         AGGTATTTTAGAATTACATGATAATATTATGTTTTATTTATTAGTTATCTTAGGTTTAG-   177
                ********** ******************************** **** ********** 

COX             TATCTTGAATGTTATATACAATTGTTATAACATATTCAAAAAATCCTATTGCATATAAAT   220
34COXFp         GATCTTGAATGTTATATACAATTGTTATAACATATTCAAAAAATCCTATTGCATATAAAT   192
34COXRp         TATCTTGAATGTTATATACAATTGTTATAACATATTCAAAAAATCCTATTGCATATAAAT   237
                 ***********************************************************

COX             ATATTAAACATGGACAAACTATTGAAGTTATTTG---AACAATTTTTCCAGCTGTAATTT   277
```

```
34COXFp      ATATTAAACATGGACAAACTATTGAAAGTTATTTGCAACAATTTTTTCCAGCTGTAATAT   252
34COXRp      ATATTAAACATGGACAAACTATTGAAGTTA-TTTGA-A-CAATTTTTCCAGCTGTAATTT   294
             **************************. *: **    * .*:****************:*

COX          -TATTAATTATTGCTTTTCCTTCATTTATTTTATTATATTTATGTGATGAAGTTATTTCA   336
34COXFp      TTATTAATTATTGCTTTTCCTTCATTTATTTTATTATATTTATGTGATGAAGTTATTTCA   312
34COXRp      -TATTAATTATCGCTTTTCCTTCATTTATTCTATTATATTTATGTGATGAAGTTATTTCA   353
              ********** ****************** *****************************


COX          CCAGCTATAACTATTAAAGCTATTGGATATCAATGATATTGAAAATATGAATATTCAGAT   396
34COXFp      CCAGCTATAACTATTAAAGCTATTGCATATCATTGATATTGACAATATGAATATTCTGAT   372
34COXRp      CCAGCTATAACTATTAAAGCTATTGGATATCAATGATATTGAAAATATGAATATTCAGAT   413
             ************************ *******:*********.*************:***

COX          TTTATTAATGATAGTGGTGAAACTGTTGAATTTGAATCATATGTTATTCCTGATGAATTA   456
34COXFp      TTTATTATTGATAGTGGTGAAACTGATGAATTTGAATCATATGTTATTCCTGATGAATTA   432
34COXRp      TTTATTAATGATAGTGGTGAAACTGTTGAATTTGAATCATATGTTATTCCTGATGAATTA   473
             *******:*****************:**********************************

COX          TTAGAAGAAGGTCAATTAAGATTATTAGATACTGATACTTCTATAGTTGTACCTGTAGAT   516
34COXFp      TTGGAAGAGCGTCAATTAAGATTATTACATCCGGATACTTCGATCATTGTACCTGAATAT   492
34COXRp      TTAGAAGAAGGTCAATTAAGATTATTAGATACTGATACTTCTATAGTTGTACCTGTAGAT   533
             **.*****. ***************** **.* ******** **..*********:* **

COX          ACACATATT-AGATTCGTTGTAACAGCTGCTGATGTTATTCATGATTTTGCTATTCCAAG   575
34COXFp      ACTCTCCTTGTATTACATTGTTACAGCTGCTGAGATTATTTGAGAATTTGCTATCCCTTG   552
34COXRp      ACACATATC-AGATTCGTTGTAACAGCTGCTGATGTTATTCATGATTTTGCTATTCCAAG   592
             **:*: .*  :.:*:*.****:*********** .***** .:**:******** **::*

COX          TTTAGGTATTAAAGTTGATGCTACTCCTGGTAGATTAAATCAAGTTTCTGCTTTAATTCA   635
34COXFp      TTTATGTATTTCAGTAGACGCTACTCCAGATAAATTAAATCTAATTCCTACTATAATTCT   612
34COXRp      TTTAGGTATTAAAGTTGATGCTACTCCTGGTAGATTAAATCAAGTTTCTGCTTTAATTCA   652
             **** *****:.***:** ********:*.**.********:*.** **.**:******:

COX          AAGAGAAGGTGTCTTCTA--TGGAGCATGTTCTGAGTTGTGTGGGAC-AGGTCATGCAAA   692
34COXFp      GTGATTCTGAGACACTCTTCTTGAATTTGTTGTTGCTTAAGCCGGACAAGAACATGCATA   672
34COXRp      AAGAGAAGGTGTCTTCTAT--GGAGCATGTTCTGAGTTGTGTGGGAC-AGGTCATGCAAA   709
             .:** :. *:*:*:   :    **. :**** * . **.:*  **** **.:******:*

COX          TATGCCAATT-AAGATCGAAG-CAGTATCA-TTACCTAAATTTTTGG-AATGATTAAAT-   747
34COXFp      TAGGTGTTGTTA-CATCAATCACAATAACATGTACGTTAAATTGTAGTCTTGATAAAATC   731
34COXRp      TATGCCCAATTAAGATCGAA-------GCCAGTTCCATCATT------------------   744
             ** *   : * *  ***.*:        *.  *:* ::.*:*

COX          -GAACAATAA--------------      756
34COXFp      TAACCCTTAAAAAAAAAAAAAAAA      754
34COXRp      ------------------------      744
```
