# Supplementary material for: Saccharomyces cerevisiae DJ-1 paralogs maintain genome integrity through glycation repair of nucleic acids and proteins
Source: eLife. 2023 Aug 7;12:e88875. doi: 10.7554/eLife.88875 (PMC10431920; doi:10.7554/eLife.88875)
Supplement: Figure 6—source data 5. [file elife-88875-fig6-data5.zip › Figure 6F.pdf]

Mutation profile of SOD1, RAD14, and COX2

Mutations   Original sequence   **Fp-Forward primer; Rp-reverse primer**

WT

SOD1

```
WTFp      -----------------------TTTAAAAGGGGGGGGCCGGGGTTCTCTGGTGTTGTCA      37
SOD1      -----ATGGTTCAAGCAGTCGCAGTGTTAAAGGGTGATGCCGGTGTCTCTGGTGTTGTCA      55
WTRp      TTTATATGGTTCAAGCAGTCGCAGTGTTAAAGGGTGATGCCGGTGTCTCTGGTGTTGTCA      60
                                  *   ** *** *   * **  ***************

WTFp      AGTTTCGACAGGCTTCCGAATCCGAGCCAACCACTGTTCTCTTACAAAATCGCTGGTAAC      97
SOD1      AGTTCGAACAGGCTTCCGAATCCGAGCCAACCACT-GTCTCTTACGAGATCGCTGGTAAC      114
WTRp      AGTTCGAACAGGCTTCCGAATCCGAGCCAACCACT-GTCTCTTACGAGATCGCTGGTAAC      119
          ****   ****************************  ******** * ************

WTFp      AGTCCTAACGCAAAACGTGGGTTCCACATTCATGAGTTTGGAAATGCCACCAAGGGTTGT      157
SOD1      AGTCCTAACGCAGAACGTGGGTTCCACATTCATGAGTTTGGAGATGCCACCAATGGTTGT      174
WTRp      AGTCCTAACGCAGAACGTGGGTTCCACATTCATGAGTTTGGAGATGCCACCAATGGTTGT      179
          ************ ***************************** ********** ******

WTFp      GTCTCTGCTGGTCCTCACTTCAATCCTTTCAAAAAAACACATGGTGCTCCAACTGACAAA      217
SOD1      GTCTCTGCTGGTCCTCACTTCAATCCTTTCAAGAAGACACATGGTGCTCCAACTGACGAA      234
WTRp      GTCTCTGCTGGTCCTCACTTCAATCCTTTCAAGAAGACACATGGTGCTCCAACTGACGAA      239
          ******************************** ** ********************* **

WTFp      TTCAAACATGTCGGTGACATGGGTAACGTAAAAACGGACAAAAATGGTGTGGCCAAGGGC      277
SOD1      GTCAGACATGTCGGTGACATGGGTAACGTAAAGACGGACGAAAATGGTGTGGCCAAGGGC      294
WTRp      GTCAGACATGTCGGTGACATGGGTAACGTAAAGACGGACGAAAATGGTGTGGCCAAGGGC      299
          *** *************************** ****** ********************

WTFp      TCCTTCAAGGACTCTTTGATCAAGCTTATCGGTCCTACCTCCGTTGTAGGCAAAAGCGTC      337
SOD1      TCCTTCAAGGACTCTTTGATCAAGCTTATCGGTCCTACCTCCGTTGTAGGCAGAAGCGTC      354
WTRp      TCCTTCAAGGACTCTTTGATCAAGCTTATCGGTCCTACCTCCGTTGTAGGCAGAAGCGTC      359
          **************************************************** *******

WTFp      TTTATCCACGCCGGCCAAAATGACTTAGGTAAGGGTGACACTGAAAAATCTTTGAAGACT      397
SOD1      GTTATCCACGCCGGCCAAGATGACTTAGGTAAGGGTGACACTGAAGAATCTTTGAAGACT      414
WTRp      GTTATCCACGCCGGCCAAGATGACTTAGGTAAGGGTGACACTGAAGAATCTTGAAGACTG      419
          ***************** ************************** ******  *

WTFp      GGTAATGCCGGTCCAAAACCAGCCTGTGGTGTCATTGGTCTAACACAACTAAAAAA 453
SOD1      GGTAATGCCGGTCCAAGACCAGCCTGTGGTGTCATTGGTCTAACCAACTAA----- 465
WTRp      GTAATGCCCGTTCCCAAGGCCCGCCCCCCAA------------------------- 450
          *  *   *** *** *   *   *
```

RAD14

```
WTRp      ------------------------------------------------------------      0
WTFp      ------------------------------------------------------------      0
RAD       ATGACTCCCGAACAAAAGGCCAAACTAGTATGTGTGAAAATGATTCTGTGTTTGTATTTT      60


WTRp      ------------------------------------------------------------      0
WTFp      ------------------------------------------------------------      0
RAD       AACCGTGGGTTTCTTTTTACTAACAATTTAACGATGAGATGAGCTGTGCAGGAGGCTAAC      120


WTRp      ------------------------------------------------------------      0
WTFp      ------------------------------------------------------------      0
RAD       AGGAAATTAGCAATAGAACGGTTAAGAAAAAGGGGAATACTGAGTAGCGACCAATTGAAT      180
```

```
WTRp          ------------------------------------------------------------      0
WTFp          ------------------------------------------------------------      0
RAD           CGAATAGAAAGTAGGAATGAACCTTTAAAAACCCGGCCTCTCGCAGTTACTAGTGGCAGC      240

WTRp          ------------------------------------------------------------      0
WTFp          ------------------------------------------------------------      0
RAD           AATCGGGATGATAATGCAGCAGCCGCAGTACATGTGCCAAATCATAATGGACAACCGTCT      300

WTRp          ------------------------------------------------------------      0
WTFp          ------------------------------------------------------------      0
RAD           GCGCTTGCTAACACTAACACTAACACTACTTCACTTTATGGTAGCGGAGTAGTTGATGGA      360

WTRp          -----------TATGACTTTTCGATGTACAAGGTCATCGGATAGAATCAGGCAGTGCGTA      49
WTFp          -----------------------------------------------------------A      1
RAD           AGTAAAAGGGATATGTCGGTACTCGACAAAAGGCCAACGGATAGAATCAGACCTAGCATA      420
                                                                         *

WTRp          CATAATCAAGATTACATTGAGTACGATTTTCATCACTACCTGCATCAGAATGGTCGTTAT      109
WTFp          ATGAAACAAGATTACATTGAGTACGATTTTGCCACCATGCAGAACTTGAATGGTGGTTAT      61
RAD           AGGAAACAAGATTACATTGAGTACGATTTTGCCACCATGCAGAACTTGAATGGTGGTTAT      480
                 ** ************************     *   * * *   ******* *****

WTRp          ATCGACGAATAGGACAAGCTTCCAAATTCTTTTCTTTTAATATGACCGTGTATTTGAATG      169
WTFp          ATCAACCCTAAGGACAAGCTTCCAAATTCTG-ACTTTACCGATGACCAAGAATTTGAATC      120
RAD           ATCAACCCTAAGGACAAGCTTCCAAATTCTG-ACTTTACCGATGACCAAGAATTTGAATC      539
              *** **    ********************   ****    ******  * ********

WTRp          TGAGTGTGGATAGTAGCAGCAGAAGACACTACAGGACTGGAAAAAGGACCAACTTGAACG      229
WTFp          TGAGTTTGGATCTAAAAAGCAGAAGACACTACAGGACTGGAAAAAGGAACAACTTGAACG      180
RAD           TGAGTTTGGATCTAAAAAGCAGAAGACACTACAGGACTGGAAAAAGGAACAACTTGAACG      599
              ***** *****   *  ******************************* ***********

WTRp          GAAAATTTTGTCAGAAAATGCACCTTCTTCATAGCATCTTTCAAATGCGCCGCAATTTAT      289
WTFp          GAAAATGCTGTACGAAAATGCACCTCCTCCAGAGCATATTTCAAAGGCGCCGAAATGTAT      240
RAD           GAAAATGCTGTACGAAAATGCACCTCCTCCAGAGCATATTTCAAAGGCGCCGAAATGTAT      659
              ******  ***  ************ ** ** ***** ******* ****** *** ***

WTRp          GGAAGCTAAGGTTTATATTGAGATGGATCCAAATATACGCGATGTGTTCAAGTTCCAAGA      349
WTFP          TGAATGTCATATTAATATTGAGATGGATCCTGTGCTACATGATGTGTTCAAGTTACAAGT      300
RAD           TGAATGTCATATTAATATTGAGATGGATCCTGTGCTACATGATGTGTTCAAGTTACAAGT      719
               ***  * *  ** ****************     ***  ************** ****

WTRp          TTGTAAACTGGGTTTTAAGGAGCATCCAAAAAAATATTCATCATTGAAGAAAACATAAGG      409
WTFp          TTGTAAACAGTGTTCTAAGGAGCATCCAGAAAAGTATGCACTACTGACGAAAACAGAATG      360
RAD           TTGTAAACAGTGTTCTAAGGAGCATCCAGAAAAGTATGCACTACTGACGAAAACAGAATG      779
              ******** * *** ************* **** *** **  * *** ******* ** *

WTRp          TAAAGAAGAATATTCTTTACCAGACCCTGAAGTCAATGATGCGGATTTTTTTCATAGATC      469
WTFp          TAAGGAAGATTACTTTTTAACAGACCCCGAATTGAATGATGAGGATCTCTTTCATAGACT      420
RAD           TAAGGAAGATTACTTTTTAACAGACCCCGAATTGAATGATGAGGATCTCTTTCATAGACT      839
              *** ***** ** * **** ******* *** * ******* **** * *********

WTRp          AGAAAAGCCGAACCTTCATTCGGGGACATTTGCAAGAATGCAAGCACAGG--AAGATCGA      527
WTFp          AGAAAAGCCGAACCCTCATTCGGGGACATTTGCAAGAATGCAACTATTTGTTAGATGTGA      480
RAD           AGAAAAGCCGAACCCTCATTCGGGGACATTTGCAAGAATGCAACTATTTGTTAGATGTGA      899
              ************** ****************************  *   *  *     **

WTRp          AGTGGAAGCCTTTGCGTTCAAGAAATGGGGTGGAGAAGAAGGTTTTGATGAGGAAGGGCA      587
WTFp          AGTGGAAGCCTTTGCGTTCAAGAAATGGGGTGGAGAAGAAGGTTTAGATGAGGAATGGCA      540
RAD           AGTGGAAGCCTTTGCGTTCAAGAAATGGGGTGGGGAAGAAGGTTTAGATGAGGAATGGCA      959
              ********************************* *********** ********* ****

WTRp          ACGTCGTGAAGAAGGAAAGGCTCACAGAAGGGAGAAAAAATACGAAAAGAAAATCAAGGA      647
WTFp          ACGTCGTGAAGAAGGAAAGGCTCACAGAAGGGAGAAAAAATACGAAAAGAAAATCAAGGA      600
```

```
RAD        ACGTCGTGAAGAAGGAAAGGCTCACAGAAGGGAGAAAAAATACGAAAAGAAAATCAAGGA      1019
           ************************************************************

WTRp       AATGCGATTGAAAACAAGAGCTCAAGAATATATTAATAGATTAAGAGAAAAGAAGCATGG      707
WTFp       AATGCGACTGAAAACAAGAGCTCAAGAATATACTAATAGATTAAGAGAAAAGAAGCATGG      660
RAD        AATGCGACTGAAAACAAGAGCTCAAGAATATACTAATAGATTAAGGGAAAAGAAGCATGG      1079
           ******* ************************ ************ **************

WTRp       GAAAGCCCATATTCATCATTTTAGTGATCCAGTTGATGGAGGTATTGATGAAGACGGTTA      767
WTFp       GAAAGCCCATATTCATCATTTTAGTGATCCAGTTGATGGAGGTATTGATGAAGACGGTTA      720
RAD        GAAAGCCCATATTCATCATTTTAGTGATCCAGTTGATGGAGGTATTGATGAAGACGGTTA      1139
           ************************************************************

WTRp       TCAAATTCAAAGTATG--------------------------------------------      783
WTFp       TCAAATTCAAAGAAGAAGATGTACAGACTGCGGGCTAGAAACTGAAGAAATTGACATTTA      780
RAD        TCAAATTCAAAGAAGAAGATGTACAGACTGCGGGCTAGAAACTGAAGAAATTGACATTTA      1199
           ************ *

WTRp       -------       783
WTFp       AAATGTC       787
RAD        A------       1200
```

COX2

```
WTCoxFp      -----------------------------------------------------------T   1
Cox2         ---------ATGTTAGATTTATTAAGATTACAATTAACAACATTCATTATGAATGATGTA   51
WTCoxRp      TTTTTTTTTATGTTAGATTTATTAAGATTACAATTAACAACATTCATTATGAATGATGTA   60


WTCoxFp      CAACACCTTATGCATGTTTATTTTCAGGATTCAGCAACACCAAATCAAGAAGGTATTTTA   61
Cox2         CCAACACCTTATGCATGTTATTTTCAGGATTCAGCAACACCAAATCAAGAAGGTATTTTA   111
WTCoxRp      CCAACACCTTATGCATGTTATTTTCAGGATTCAGCAACACCAAATCAAGAAGGTATTTTA   120
             * *   * *        *******************************************

WTCoxFp      GAATTACATGATAATATTATGTTTTATTTATTAGTTATTTTAGGTTTAGTATCTTGAATG   121
Cox2         GAATTACATGATAATATTATGTTTTATTTATTAGTTATTTTAGGTTTAGTATCTTGAATG   171
WTCoxRp      GAATTACATGATAATATTATGTTTTATTTATTAGTTATTTTAGGTTTAGTATCTTGAATG   180
             ************************************************************

WTCoxFp      TTATATACAATTGTTATAACATATTCAAAAAATCCTATTGCATATAAATATATTAAACAT   181
Cox2         TTATATACAATTGTTATAACATATTCAAAAAATCCTATTGCATATAAATATATTAAACAT   231
WTCoxRp      TTATATACAATTGTTATAACATATTCAAAAAATCCTATTGCATATAAATATATTAAACAT   240
             ************************************************************

WTCoxFp      GGACAAACTATTGAAGTTATTTGAACAATTTTTCCAGCTGTAATTTTATTAATTATTGCT   241
Cox2         GGACAAACTATTGAAGTTATTTGAACAATTTTTCCAGCTGTAATTTTATTAATTATTGCT   291
WTCoxRp      GGACAAACTATTGAAGTTATTTGAACAATTTTTCCAGCTGTAATTTTATTAATTATTGCT   300
             ************************************************************

WTCoxFp      TTTCCTTCATTTATTTTATTATATTTATGTGATGAAGTTATTTCACCAGCTATAACTATT   301
Cox2         TTTCCTTCATTTATTTTATTATATTTATGTGATGAAGTTATTTCACCAGCTATAACTATT   351
WTCoxRp      TTTCCTTCATTTATTTTATTATATTTATGTGATGAAGTTATTTCACCAGCTATAACTATT   360
             ************************************************************

WTCoxFp      AAAGCTATTGGATATCAATGATATTGAAAATATGAATATTCAGATTTTATTAATGATAGT   361
Cox2         AAAGCTATTGGATATCAATGATATTGAAAATATGAATATTCAGATTTTATTAATGATAGT   411
WTCoxRp      AAAGCTATTGGATATCAATGATATTGAAAATATGAATATTCAGATTTTATTAATGATAGT   420
             ************************************************************

WTCoxFp      GGTGAAACTGTTGAATTTGAATCATATGTTATTCCTGATGAATTATTAGAAGAAGGTCAA   421
Cox2         GGTGAAACTGTTGAATTTGAATCATATGTTATTCCTGATGAATTATTAGAAGAAGGTCAA   471
WTCoxRp      GGTGAAACTGTTGAATTTGAATCATATGTTATTCCTGATGAATTATTAGAAGAAGGTCAA   480
             ************************************************************

WTCoxFp      TTAAGATTATTAGATACTGATACTTCTATAGTTGTACCTGTAGATACACATATTAGATTC   481
Cox2         TTAAGATTATTAGATACTGATACTTCTATAGTTGTACCTGTAGATACACATATTAGATTC   531
WTCoxRp      TTAAGATTATTAGATACTGATACTTCTATAGTTGTACCTGTAGATACACATATTAGATTC   540
             ************************************************************
```

```
WTCoxFp       GTTGTAACAGCTGCTGATGTTATTCATGATTTTGCTATTCCAAGTTTAGGTATTAAAGTT    541
Cox2          GTTGTAACAGCTGCTGATGTTATTCATGATTTTGCTATTCCAAGTTTAGGTATTAAAGTT    591
WTCoxRp       GTTGTAACAGCTGCTGATGTTATTCATGATTTTGCTATTCCAAGTTTAGGTATTAAAGTT    600
              ************************************************************

WTCoxFp       GATGCTACTCCTGGTAGATTAAATCAAGTTTCTGCTTTAATTCAGAGAGAAGGTGTCTTC    601
Cox2          GATGCTACTCCTGGTAGATTAAATCAAGTTTCTGCTTTAATTCAAAGAGAAGGTGTCTTC    651
WTCoxRp       GATGCTACTCCTGGTAGATTAAATCAAGTTTCTGCTTTAATTCAAAGAGAAGGTGTCTTC    660
              ******************************************** ***************

WTCoxFp       TATGGAGCATGTTCTGAGTTGTGTGGACAGGTCATGCAAATATGCCAATTAAGATCGAA    661
Cox2          TATGGAGCATGTTCTGAGTTGTGTGGACAGGTCATGCAAATATGCCAATTAAGATCGAA    711
WTCoxRp       TATGGAGCATGTTCTGAGTTGTGTGGACAGGTCATGCAAAATATGCCCAATTAAGATCG    720
              *****************************************     *       *

WTCoxFp       GCAGTATCATTACCTAAATTGTTGGAATGATTAAATGAACAAAAAAAAAAA    712
Cox2          GCAGTATCATTACCTAAATTTTTGGAATGATTAAATGAACAATAA------    756
WTCoxRp       A--------------------------------------------------    721
```

ΔQ

RAD14

```
QRADRp        TTTTTTTTTTTAAACACCCCCCCCCCGCTTTTAAAGAATCCCCCCGAGATTTAAAGGGCC    60
QRADFp        ------------------------------------------------------------    0
RAD14         -------------------------------------ATGACTCC-CGAAC-AAAAGGCC    21

QRADRp        AAAATTTTTTTAGGTTTTGTGAAAAGGATTTTTGGTTTTTTTTTTTTTTTGAATTGGTTT    120
QRADFp        -------------------------TAATTCTGT----GTTTGTATTTTAACCGTGGGTT    31
RAD14         AAAC------TAGTATGTGTGAAAATGATTCTGT----GTTTGTATTTTAACCGTGGGTT    71
                                         *** *       *** * ****     *** **

QRADRp        TTTTTTTTTCAACAATTACCTCCATGAAAGTGGGTTCGGTCAAAATGGGTGAACATGGGA    180
QRADFp        TCTTTTTACTAACAAT-TTAACGATGAGGATGAGCTGTGCAGG----AGG-CTAACAGGA    85
RAD14         TCTTTTTACTAACAAT-TTAACGATG-AGATGAGCTGTGCAGG----AGG-CTAACAGGA    124
              * *****   ******     * ***    ** * *  *         *     *  ***

QRADRp        CTTTTCCCATGGTTTCTGTTTATGTAAAAAGGAGTAGTACTGTTGTTGGGGCTCCAGTTG    240
QRADFp        AATTAG-CAATAGAACGGTTAAGAAAAAGGGGA---ATACT---GAGTAGCGACCAA---    135
RAD14         AATTAG-CAATAGAACGGTTAAGAAAAAGGGGA---ATACT---GAGTAGCGACCAA---    174
                **   **      * *** *   ***  ***    ****   *    *   ***

QRADRp        ATCAGTAGCAGTTACCTTGGAATTTAACCTTCGAAAAACCTCGTCCTTTCGGCAGATCCC    300
QRADFp        TTGAATCGAATAGAAAGTAGGAATGAACCTTTAAAAACCCGGC--CTCTCGCAGTTA---    190
RAD14         TTGAATCGAATAGAAAGTAGGAATGAACCTTTAAAAACCCGGC--CTCTCGCAGTTA---    229
               * * * * *   *   * * * * ******  **** **     ** ***

QRADRp        TTTCCGTCATCAATGAGGGATGCCTATTCCTAGCTAGTCAGCAGTACTTTTCCACAACTC    360
QRADFp        -----CTAGTGGCAGCAATCGGGATGATAATGCAGCAGCCGCAGTACATGTGCCAAAT-C    244
RAD14         -----CTAGTGGCAGCAATCGGGATGATAATGCAGCAGCCGCAGTACATGTGCCAAAT-C    283
                    *  *    *      *  *  *  *       * ******* * * *  **  *

QRADRp        TTAATGGACGCAAAGTGTGTGCATGTTACCTCTAACAATTACTTTCATTCCTCGCAAATG    420
QRADFp        ATAATG-GACAACCGTCTGCGCTTGTTAACACTAACACTAACACTACTTCACT------T    297
RAD14         ATAATG-GACAACCGTCTGCGCTTGCTAACACTAACACTAACACTACTTCACT------T    336
               *****     *  ** ** ** ** ** * ****** * **  *  ***

QRADRp        GTAGCGGAATTAGTTGTTTGGAAGTAAATAGGGATGTGAACGATATTCGACAAAAGGACA    480
QRADFp        TATGGTAGCGGAGTAGTTGATGGAAGTAAAAGGGATGCGACGGTACTCGACAAAAGGCCA    357
RAD14         TATGGTAGCGGAGTAGTTGATGGAAGTAAAAGGGATGCGTCGGTACTCGACAAAAGGCCA    396
                 *       *** ***         * * **       ** ** *********** **

QRADRp        TCGGATTGAATCAGACCTTGCTTATCGAAACAAGATGACATTGAGTGTGATGCAGCCACC    540
```

```
QRADFp      ACGGATAGAATCAGACCTAGCATAAGGAAACAAGATTACATTGAGTACGATTTTGCCACC    417
RAD14       ACGGATAGAATCAGACCTAGCATAAGGAAACAAGATTACATTGAGTACGATTTTGCCACC    456
             ***** *********** ** **  ********** *********  ***   ******

QRADRp      ATGCAGAACTTGAATGGTGGTTATATCAACCTTAAGGACAAGCCTCCTAATTCTGCACTT    600
QRADFp      TTGCAGAACTTGAATGGTGGTTATATCATCCCTAAGGACAAGCTTCCAGATTCTGACTTT    477
RAD14       ATGCAGAACTTGAATGGTGGTTATATCAACCCTAAGGACAAGCTTCCAAATTCTGACTTT    516
             *************************** ** *********** ***  ******   **

QRADRp      ACTGCTGATCAAGAATAAGAATACGAGTTTGGCTCTTAAAAGCAGAAGACATCTACTGGA    660
QRADFp      ACCGCTGACCAAGAATTTGAATCTGATTTTGGATCTAAAAAGCAGAAGAC-ACTACAGGA    536
RAD14       ACCGATGACCAAGAATTTGAATCTGAGTTTGGATCTAAAAAGCAGAAGAC-ACTACAGGA    575
            ** * *** *******  ****  ** ***** *** *************  **** ***

QRADRp      GTGGAAAAAGGAACTACTTGAACGGAAAATGCTGTACGAAAACGCACCTCCTCCCGAGCA    720
QRADFp      CTGGAAGAAGGAACAACTTGAACGGAAAATGCTGTACGAAAATGCACCTCCTCCAGAGCA    596
RAD14       CTGGAAAAAGGAACAACTTGAACGGAAAATGCTGTACGAAAATGCACCTCCTCCAGAGCA    635
             ***** ******* *************************** *********** *****

QRADRp      TATTTCAAAGGCGCCGAAATGTATAGAATGTCATATTATTATTGTGATGGGTCATGTGAT    780
QRADFp      TATTTCAAAGGCGCCGAAATGTATTGAATGTCATATTAATATTGAGATGGATCCTGTGCT    656
RAD14       TATTTCAAAGGCGCCGAAATGTATTGAATGTCATATTAATATTGAGATGGATCCTGTGCT    695
            ************************ ************* ***** ***** ** **** *


QRADrp      ACTTGATGTGTTCAACTTACAAGTTTGTAAACAGTGCTCTAAGGAGCATCCAGAAAAGTA    840
QRADfp      ACATGATGTGTTCAAGTTACAAGTTTGTAAACAGTGTTCTAATGAGCATCCAGAAAAGTA    716
RAD14       ACATGATGTGTTCAAGTTACAAGTTTGTAAACAGTGTTCTAAGGAGCATCCAGAAAAGTA    755
            ** ************ ******************** ***** *****************

QRADRp      TGCACTACTGACGAAAACAGAATGTAAGGAAGATTACTTTTTAACAGACCCCGACTTGAA    900
QRADFp      TGCACTACTGACGAATACAGAATGTAAAGAAGATTACTTTTTAACAGACCCCGAATTGAA    776
RAD14       TGCACTACTGACGAAAACAGAATGTAAGGAAGATTACTTTTTAACAGACCCCGAATTGAA    815
            *************** *********** ************************** *****

QRADRp      TGATGAGGATGTCTTTCATAGACTAGAAAAGCCGAACCCTCATTCGGGGACATTTGCAAG    960
QRADFp      TGATGATGATGTCTTTCATAGACTAGAAAAGCCGAACCCTCATTCGGGGACATTTGCAAG    836
RAD14       TGATGAGGATCTCTTTCATAGACTAGAAAAGCCGAACCCTCATTCGGGGACATTTGCAAG    875
            ****** *** *************************************************

QRADrp      AATGCAACTATTTGTTAGATGTGAAGCGGAAGCCTTTGCGTTCAAGAAATGGGGTGGAGA    1020
QRADfp      AATGCAACTATTTGTTAGATGTGAAGTGGAAGCCTTTGCGTTCAAGAAATGGGGTGGAGA    896
RAD14       AATGCAACTATTTGTTAGATGTGAAGTGGAAGCCTTTGCGTTCAAGAAATGGGGTGGAGA    935
            ************************** *********************************

QRADRp      AGAAGGTTTAGATGAGGAATGGCAACGTCGTGAAGAAGGAAAGGCTCACAGAAGGGAGAA    1080
QRADFp      AGAAGGTTTAGATGAGGAATGGCAACGTCGTGAAGAAGGAAAGGCTCACAGAAGGGAGAA    956
RAD14       AGAAGGTTTAGATGAGGAATGGCAACGTCGTGAAGAAGGAAAGGCTCACAGAAGGGAGAA    995
            ************************************************************

QRADRp      AAAATACGAAAAGAAAATCAAGGAAATGCGACTGAAAACAAGAGCTCAAGAATATACTAA    1140
QRADFp      AAAATACGAAAAGAAAATCAAGGAAATGCGACTGAAAACAAGAGCTCAGGAATATCCTAA    1016
RAD14       AAAATACGAAAAGAAAATCAAGGAAATGCGACTGAAAACAAGAGCTCAAGAATATACTAA    1055
            ************************************************ ****** ****

QRADRp      TAGATTAA-GAGAAAAGAAGCATGGGAAAGCCCATATTCATCATTTTAGTGATCCAGTTG    1199
QRADFp      TAGATTTAAGAAAAAAGAAGCATGGGAAAGCCCATATTCACCATTTTAGTGATCCAGTTG    1076
RAD14       TAGATTAA-GAGAAAAGAAGCATGGGAAAGCCCATATTCATCATTTTAGTGATCCAGTTG    1114
            ****** * ** **************************** *******************

QRADRp      ATGGAGGTATTGATGAAGCACGGTTATCAAATTCAAAGAAGAAGATTCAGA---------    1250
QRADFp      ATGGAGGTATTGATGAAGACGGTTATCAATTCCAAAAAAAA-AATGACAGAACTGCGGGC    1135
RAD14       ATGGAGGTATTGATGAAGACGGTTATCAAATTCAAAGAAGAAGATGTACAGACTGCGGGC    1174
            ******************   * *    *     **  *

QRADRp      ------------------------------------------------------------    1250
QRADFp      TAGAAACTGAAGATTTTTATATTTAATATTTTTTTTTTTTTAAACGCGGTCAGTAGTTTT    1195
RAD14       TAGAAACTGAAGAAATTGACATTTAA----------------------------------    1200
```

```
QRADRp       ------------- 1250
QRADFp       AAAAAAATATTAC 1208
RAD14        ------------- 1200
```

COX2

```
COX        ------------------ATGTTAGATTTATTAAGATTACAATTAACAACATTCATTATG      42
QCFp       ------------------------------------------------------------      0
QCRp       TTTTTTTTTTTTTTTTTTATTTAGAATTTATTAAGATTACAATTAACAACATTCATTATG      60

COX        AATGATGTACCAACACCT-TATGCATGTTATTTTCAGGATTCAGCAACACCAAATCAAGA      101
QCFp       ---AATGTACCAACACCTTTATGCATGTTATTTTCAGGATTCAGCAACACCAAATCAAGA      57
QCRp       AATAATGTACCAACACCT-TATGCATGTTATTTTCAGGATTCAGCAACACCAAATCAAGA      119
               ************** *****************************************

COX        AGGTATTTTAGAATTACATGATAATATTATGTTTTATTTATTAGTTATTTTAGGTTTAGT      161
QCFp       AGGTATTTTAGAATTACATGATAATATTATGTTTTATTTATTAGTTATTTTAGGTTTAGT      117
QCRp       AGGTATTTTAGAATTACATGATAATATTATGTTTTATTTATTAGTTATTTTAGGTTTAGT      179
           ************************************************************

COX        ATCTTGAATGTTATATACAATTGTTATAACATATTCAAAAAATCCTATTGCATATAAATA      221
QCFp       ATCTTGAATGTTATATACAATTGTTATAACATATTCAAAAAATCCTATTGCATATAAATA      177
QCRp       ATCTTGAATGTTATATACAATTGTTATAACATATTCAAAAAATCCTATTGCATATAAATA      239
           ************************************************************

COX        TATTAAACATGGACAAACTATTGAAGTTATTTGAACAATTTTTCCAGCTGTAATTTTATT      281
QCFp       TATTAAACATGGACAAACTATTGAAGTTATTTGAACAATTTTTCCAGCTGTAATTTTATT      237
QCRp       TATTAAACATGGACAAACTATTGAAGTTATTTGAACAATTTTTCCAGCTGTAATTTTATT      299
           ************************************************************

COX        AATTATTGCTTTTCCTTCATTTATTTTATTATATTTATGTGATGAAGTTATTTCACCAGC      341
QCFp       AATTATTGCTTTTCCTTCATTTATTTTATTATATTTATGTGATGAAGTTATTTCACCAGC      297
QCRp       AATTATTGCTTTTCCTTCATTTATTTTATTATATTTATGTGATGAAGTTATTTCACCAGC      359
           ************************************************************

COX        TATAACTATTAAAGCTATTGGATATCAATGATATTGAAAATATGAATATTCAGATTTTAT      401
QCFp       TATAACTATTAAAGCTATTGGATATCAATGATATTGAAAATATGAATATTCAGATTTTAT      357
QCRp       TATAACTATTAAAGCTATTGGATATCAATGATATTGAAAATATGAATATTCAGATTTTAT      419
           ************************************************************

COX        TAATGATAGTGGTGAAACTGTTGAATTTGAATCATATGTTATTCCTGATGAATTATTAGA      461
QCFp       TAATGATAGTGGTGAAACTGTTGAATTTGAATCATATGTTATTCCTGATGAATTATTAGA      417
QCRp       TAATGATAGTGGTGAAACTGTTGAATTTGAATCATATGTTATTCCTGATGAATTATTAGA      479
           ************************************************************

COX        AGAAGGTCAATTAAGATTATTAGATACTGATACTTCTATAGTTGTACCTGTAGATACACA      521
QCFp       AGAAGGTCAATTAAGATTATTAGATACTGATACTTCTATAGTTGTACCTGTAGATACACA      477
QCRp       AGAAGGTCAATTAAGATTATTAGATACTGATACTTCTATAGTTGTACCTGTAGATACACA      539
           ************************************************************

COX        TATTAGATTCGTTGTAACAGCTGCTGATGTTATTCATGATTTTGCTATTCCAAGTTTAGG      581
QCFp       TATTAGATTCGTTGTAACAGCTGCTGATGTTATTCATGATTTTGCTATTCCAAGTTTAGG      537
QCRp       TATTAGATTCGTTGTAACAGCTGCTGATGTTATTCATGATTTTGCTATTCCAAGTTTAGG      599
           ************************************************************

COX        TATTAAAGTTGATGCTACTCCTGGTAGATTAAATCAAGTTTCTGCTTTAATTCAAAGAGA      641
QCFp       TATTAAAGTTGATGCTACTCCTGGTAGATTAAATCAAGTTTCTGCTTTAATTCAAAGAGA      597
QCRp       TATTAAAGTTGATGCTACTCCTGGTAGATTAAATCAAGTTTCTGCTTTAATTCAAAGAGA      659
           ************************************************************

COX        AGGTGTCTTCTATGGAGCATGTTCTGAGTTGTGTGGGACAGGTCATGCAAATATGCCAAT      701
```

```
QCFp      AGGTGTCTTCTATGGAGCATGTTCTGAGTTGTGTGGGACAGGTCATGCAAATATGCCAAT      657
QCRp      AGGTGTCTTCTATGGAGCATGTTCTGAGTTGTGTGGGACAGGTCATGCAAATATGCCAAT      719
          ************************************************************

COX       TAAGATCGAAGCAGTATCATTACCTAAATTTTTGGAATGATTAAATGAACAATAA-----      756
QCFp      TAAGATCGAAGCAGTATCATTACCTAAATTTTTGGAATGATTAATGACACCAAAAAAAAA      717
QCRp      TAAGATTGA---------------------------------------------------      728
          ****** **

COX       ---------       756
QCFp      AAAAAAAAA       726
QCRp      ---------       728
```

SOD1

```
QFP       ---------GGCCATGCCATCATGCAGTCAG-------------CCTGTGAGTGTCATTG      38
SOD1      --------------ATGGTTCAAGCAGTCGCAGTGTTAAAGGGTGATGCCGGTGTCTCTG      46
QRP       AAGTCGCGGATCCATGGTTTCAAGCAGTCGCAGTGTTAAAGGGTGATGCCGGTGTTTTCG      60
                             *** ******                 **   ****    *

QFP       GTCTAACCAAC-GATTACAGGATGACG----------ACGATAAGTAAGTCGACGCGTAC      87
SOD1      GTGTTGTCAAGTTCGAACAGGCTTCCGAATCCGAGCCAACCACTGTCTCTTACGAGATCG      106
QRP       GTGTTGTCAAGTTCGAACAGGCTTTCGAATCCGAGCCAACCACTGTCTTTTACGAGATCG      120
          ** *   ***       ***** *  **          *      **   *       *

QFP       GAGTCGCCGTGCATGATATGGAATCTCGTGTAATCACTGTCCAATATGGAGATGCGGCGT      147
SOD1      CTGGTAACAGTCCTAACGCAGAACGTGGGTTCCACATTCATGAGTTTGGAGATGCCACCA      166
QRP       CTGGTAACAGTCCTAACGCAGAACGTGGGTTCCACATTCATGAGTTTGGAGATGCCACCA      180
           *    *   * * *     ***  * *  *    ** *     * * *********   *

QFP       CTGGGTGTGTCCCTGCTGGTCCTCACTTCAATCCTTTCAAGAAGACACCTGGTGCTCCAA      207
SOD1      ATGGTTGTGTCTCTGCTGGTCCTCACTTCAATCCTTTCAAGAAGACACATGGTGCTCCAA      226
QRP       ATGGTTGTGTCTCTGCTGGTCCTCACTTCAATCCTTTCAAGAAGACACCTGGTGCTCCAA      240
          *** ****** ************************************ ***********

QFP       CTGACGAAGTCAGACATGTCGGTGACATGGGTAACGTAAAGACGGACGAAAATGGTGTGG      267
SOD1      CTGACGAAGTCAGACATGTCGGTGACATGGGTAACGTAAAGACGGACGAAAATGGTGTGG      286
QRP       CTGACGAAGTCAGACATGTCGGTGACATGGGTAACGTAAAGACGGACGAAAATGGTGAGG      300
          ********************************************************* **

QFP       CCAAGGGCTCCTTCAAGGACTCTTTGATCAAGCTTATCGGTCCTACCTCCGTTGTAAGCA      327
SOD1      CCAAGGGCTCCTTCAAGGACTCTTTGATCAAGCTTATCGGTCCTACCTCCGTTGTAGGCA      346
QRP       CCAAGGGGTCCTTCGTAAACTGGTGCAAGACCTGCCTGGGGTTTCATTGGTTTAACCAAG      360
          ******* ******     ***   *  *  *      * **    *   *    **

QFP       GAAGCGTCGTTATCCACGCCGGCCAAGATGACTTAGG-------TAAGGGTGACACTGAA      380
SOD1      GAAGCGTCGTTATCCACGCCGGCCAAGATGACTTAGG-------TAAGGGTGACACTGAA      399
QRP       GA--------TTACAAGGATGACGACGATAAGTAAGTCGACGCGTACGCGTGATCCAGAA      412
          **         *  * * *  * * * *** * * **        ** * ****  * ***

QFP       GAATCTTTGAAGACTGGTAATGCCGGTCCAAGACCAGCCTGTGGTGTCATTGGTCTAACC      440
SOD1      GAATCTTTGAAGACTGGTAATGCCGGTCCAAGACCAGCCTGTGGTGTCATTGGTCTAACC      459
QRP       TCA-TGCGGGTGCCGTGTAATGCCGGTCCAAGACCAGTCCTGATAT--------------      457
            *     *  * *  ******************** *


QFP       AACGATTACGAGGATGACGACGATAACTAAGTCAACGCGAAAGA     484
SOD1      AACTAA--------------------------------------     465
QRP       --------------------------------------------      457
```

HSP31

COX2

```
COX          -------------------ATG-TTAGATTTATTAAGATTACAATTAACAACATTCATTA      40
31CFp        ------------------------------------------------------------      0
31CRp        TTTTTTTTTTTTTTTTTTAATGTTTGGATTTATTAAGATTACAATTAACAACATTCATTA      60


COX          TGAATGATGTACCAACACCT-TATGCATGTTATTTTCAGGATTCAGCAACACCAAATCAA      99
31CFp        ----TAATGTACCAACACCTTTATGCATGTTATTTTCAGGATTCAGCAACACCAAATCAA      56
31CRp        TGAATGATGTACCAACACCT-TATGCATGTTATTTTCAGGATTCAGCAACACCAAATCAA      119
                 *.************** ***************************************

COX          GAAGGTATTTTAGAATTACATGATAATATTATGTTTTATTTATTAGTTATTTTAGGTTTA      159
31CFp        GAAGGTATTTTAGAATTACATGATAATATTATGTTTTATTTATTAGTTATTTTAGGTTTA      116
31CRp        GAAGGTATTTTAGAATTACATGATAATATTATGTTTTATTTATTAGTTATTTTAGGTTTA      179
             ************************************************************

COX          GTATCTTGAATGTTATATACAATTGTTATAACATATTCAAAAAATCCTATTGCATATAAA      219
31CFp        GTATCTTGAATGTTATATACAATTGTTATAACATATTCAAAAAATCCTATTGCATATAAA      176
31CRp        GTATCTTGAATGTTATATACAATTGTTATAACATATTCAAAAAATCCTATTGCATATAAA      239
             ************************************************************

COX          TATATTAAACATGGACAAACTATTGAAGTTATTTGAACAATTTTTCCAGCTGTAATTTTA      279
31CFp        TATATTAAACATGGACAAACTATTGAAGTTATTTGAACAATTTTTCCAGCTGTAATTTTA      236
31CRp        TATATTAAACATGGACAAACTATTGAAGTTATTTGAACAATTTTTCCAGCTGTAATTTTA      299
             ************************************************************

COX          TTAATTATTGCTTTTCCTTCATTTATTTTATTATATTTATGTGATGAAGTTATTTCACCA      339
31CFp        TTAATTATTGCTTTTCCTTCATTTATTTTATTATATTTATGTGATGAAGTTATTTCACCA      296
31CRp        TTAATTATTGCTTTTCCTTCATTTATTTTATTATATTTATGTGATGAAGTTATTTCACCA      359
             ************************************************************

COX          GCTATAACTATTAAAGCTATTGGATATCAATGATATTGAAAATATGAATATTCAGATTTT      399
31CFp        GCTATAACTATTAAAGCTATTGGATATCAATGATATTGAAAATATGAATATTCAGATTTT      356
31CRp        GCTATAACTATTAAAGCTATTGGATATCAATGATATTGAAAATATGAATATTCAGATTTT      419
             ************************************************************

COX          ATTAATGATAGTGGTGAAACTGTTGAATTTGAATCATATGTTATTCCTGATGAATTATTA      459
31CFp        ATTAATGATAGTGGTGAAACTGTTGAATTTGAATCATATGTTATTCCTGATGAATTATTA      416
31CRp        ATTAATGATAGTGGTGAAACTGTTGAATTTGAATCATATGTTATTCCTGATGAATTATTA      479
             ************************************************************

COX          GAAGAAGGTCAATTAAGATTATTAGATACTGATACTTCTATAGTTGTACCTGTAGATACA      519
31CFp        GAAGAAGGTCAATTAAGATTATTAGATACTGATACTTCTATAGTTGTACCTGTAGATACA      476
31CRp        GAAGAAGGTCAATTAAGATTATTAGATACTGATACTTCTATAGTTGTACCTGTAGATACA      539
             ************************************************************

COX          CATATTAGATTCGTTGTAACAGCTGCTGATGTTATTCATGATTTTGCTATTCCAAGTTTA      579
31CFp        CATATTAGATTCGTTGTAACAGCTGCTGATGTTATTCATGATTTTGCTATTCCAAGTTTA      536
31CRp        CATATTAGATTCGTTGTAACAGCTGCTGATGTTATTCATGATTTTGCTATTCCAAGTTTA      599
             ************************************************************

COX          GGTATTAAAGTTGATGCTACTCCTGGTAGATTAAATCAAGTTTCTGCTTTAATTCAAAGA      639
31CFp        GGTATTAAAGTTGATGCTACTCCTGGTAGATTAAATCAAGTTTCTGCTTTAATTCAAAGA      596
31CRp        GGTATTAAAGTTGATGCTACTCCTGGTAGATTAAATCAAGTTTCTGCTTTAATTCAAAGA      659
             ************************************************************

COX          GAAGGTGTCTTCTATGGAGCATGTTCTGAGTTGTGTGGGACAGGTCATGCAAAT-ATGC-      697
31CFp        GAAGGTGTCTTCTATGGAGCATGTTCTGAGTTGTGTGGGACAGGTCATGCAAAT-ATGCC      655
31CRp        GAAGGTGTCTTCTATGGAGCATGTTCTGAGTTGTGTGGGACAGGTCATGCAAAATATGCC      719
             *****************************************************: ****
```

```
COX           CAATTAAGATCGAAGCAGTATCATTACCTAAATTTTTGGAATGATTAAATGAAGAATAA-      756
31CFp         AA-TTAAGATCGAAGCAGTATCATTACCTAAATTTTTGGAATGATTAATTAAAAAAATAA      714
31CRp         CAATTAAGATTA------------------------------------------------      731
              .* ******* .

COX           ------------   756
31CFp         AAAAAAATAAAA   726
31CRp         ------------   731
```

RAD14

```
31Rp        GTTTAAATTCCCGAACACTAAGTCAA-CTTGTAGGTG--AAAATG-ATCTGTG-TTGTTT      55
31Fp        -------------------------------------------------ATTGTTTGTAT      11
RAD         ---ATGACTCCCGAACAAAAGGCCAAACTAGTATGTGTGAAAATGATTCTGTGTTTGTAT      57
                                                             : ** ****:*

31Rp        TTTAACCGTAG-GTTTCTTTTTACTAACAATTTAACGATGAGATGAGCTGTGCAGGAGGC      114
31Fp        TTTAACCGTGGGGTTTCTTTTTACTAACAATTTAACGATGAGATGAGCTGTGCAGGAGGC      71
RAD         TTTAACCGTGG-GTTTCTTTTTACTAACAATTTAACGATGAGATGAGCTGTGCAGGAGGC      116
            *********.* ************************************************

31Rp        TAACAGGAAATTAGCCATAGATCGGTTAAGAAAAAGGGGAATACTGAGTAGCGACCAATT      174
31Fp        TAACAGGAAATTAGCAATAGAACGGTTAAGAAAAAGGGGAATACTGAGTAGCGACCAATT      131
RAD         TAACAGGAAATTAGCAATAGAACGGTTAAGAAAAAGGGGAATACTGAGTAGCGACCAATT      176
            ***************.*****:**************************************

31Rp        GAATCGAATAGAAAGTAGGAATGAACCTTTAAAAACCCGGCCTCTCGCAGTTACTAGTGG      234
31Fp        GAATCGAATAGAAAGTAGGAATGAACCTTTAAAAACCCGGCCTCTCGCAGTTACTAGTGG      191
RAD         GAATCGAATAGAAAGTAGGAATGAACCTTTAAAAACCCGGCCTCTCGCAGTTACTAGTGG      236
            ************************************************************

31Rp        CAGCAATCGGGATGATAATGCAGCAGCCGCAGTACATGTGCCAAATCATAATGGACAACC      294
31Fp        CAGCAATCGGGATGATAATGCAGCAGCCGCAGTACATGTGCCAAATCATAATGGACAACC      251
RAD         CAGCAATCGGGATGATAATGCAGCAGCCGCAGTACATGTGCCAAATCATAATGGACAACC      296
            ************************************************************

31Rp        GTCTGCGCTTGCTAACACTAACACTAACACTACTTCACTTTATGGTAGCGGAGTAGTTGA      354
31Fp        GTCTGCGCTTGCTAACACTAACACTAACACTACTTCACTTTATGGTAGCGGAGTAGTTGA      311
RAD         GTCTGCGCTTGCTAACACTAACACTAACACTACTTCACTTTATGGTAGCGGAGTAGTTGA      356
            ************************************************************

31Rp        TGGAAGTAAAAGGGATGTGTCAGTACTCGACAAAAGGCCAACGGATAGAATCAGACCTAG      414
31Fp        TGGAAGTAAAAGGGATGCGTCGGTACTCGACAAAAGGCCAACGGATAGAATCAGACCTAG      371
RAD         TGGAAGTAAAAGGGATGCGTCGGTACTCGACAAAAGGCCAACGGATAGAATCAGACCTAG      416
            ***************** ***.**************************************

31Rp        CATAAGGAAACAAGATTACATTGAGTACGATTTTGCCACCATGCAGAACTTGAATGGTGG      474
31Fp        CATAAGGAAACAAGATTACATTGAGTACGATTTTGCCACCTTGCAGAACTTGAATGGTGG      431
RAD         CATAAGGAAACAAGATTACATTGAGTACGATTTTGCCACCATGCAGAACTTGAATGGTGG      476
            ****************************************:*******************

31Rp        TTATATCAACCCTAAGGACAAGCTTCCAAATTCTGACTTTACCGATGACCAAGAATTTGA      534
31Fp        TTATATCAACCCTAAGGACAAGCTTCCAAATTCTGACTTTACCGATGACCAAGAATTTGA      491
RAD         TTATATCAACCCTAAGGACAAGCTTCCAAATTCTGACTTTACCGATGACCAAGAATTTGA      536
            ************************************************************

31Rp        ATCTGAGTTTGGATCTAAAAAGCAGAAGACACTACAGGACTGGAAAAAGGAACAACTTGA      594
31Fp        ATCTGAGTTTGGATCTAAAAAGCAGAAGACACTACAGGACTGGAAAAAGGAACAACTTGA      551
RAD         ATCTGAGTTTGGATCTAAAAAGCAGAAGACACTACAGGACTGGAAAAAGGAACAACTTGA      596
            ************************************************************

31Rp        ACGGAAAATGCTGTACGAAAATGCACCTCCTCCAGAGCATATTTCAAAGGCGCCGAAATG      654
31Fp        ACGGAAAATGCTGTACGAAAATGCACCTCCTCCAGAGCATATTTCAAAGGCGCCGAAATG      611
RAD         ACGGAAAATGCTGTACGAAAATGCACCTCCTCCAGAGCATATTTCAAAGGCGCCGAAATG      656
            ************************************************************
```

```
31Rp      TATTGAATGTCATATTAATATTGAGATGGATCCTGTGCTACATGATGTGTTCAAGTTACA      714
31Fp      TATTGAATGTCATATTAATATTGAGATGGATCCTGTGCTACATGATGTGTTCAAGTTACA      671
RAD       TATTGAATGTCATATTAATATTGAGATGGATCCTGTGCTACATGATGTGTTCAAGTTACA      716
          ************************************************************

31Rp      AGTTTGTAAACAGTGTTCTAAGGAGCATCCAGAAAAGTATGCACTACTGACGAAAACAGA      774
31Fp      AGTTTGTAAACAGTGTTCTAAGGAGCATCCAGAAGAGTATGCACTACTGACGAATACAGA      731
RAD       AGTTTGTAAACAGTGTTCTAAGGAGCATCCAGAAAAGTATGCACTACTGACGAAAACAGA      776
          **********************************.*******************:*****

31Rp      ATGTAAGGAAGATTACTTTTTAACAGACCCCGAATTGAATGATGAGGATCTCTTTCATAG      834
31Fp      ATGTAAGGAAGATTACTTTTTAACAGACCCCGAATTGAATGATGAGGATCTCTTTCATAG      791
RAD       ATGTAAGGAAGATTACTTTTTAACAGACCCCGAATTGAATGATGAGGATCTCTTTCATAG      836
          ************************************************************

31Rp      ACTAGAAAAGCCGAACCCTCATTCGGGGACATTTGCAAGAATGCAACTATTTGTTAGATG      894
31Fp      ACTAGAAAAGCCGAACCCTCATTCGGGGACATTTGCAAGAATGCAACTATTTGTTAGATG      851
RAD       ACTAGAAAAGCCGAACCCTCATTCGGGGACATTTGCAAGAATGCAACTATTTGTTAGATG      896
          ************************************************************

31Rp      TGAAGTGGAAGCCTTTGCGTTCAAGAAATGGGGTGGAGAAGAAGGTTTAGATGAGGAATG      954
31Fp      TGAAGTGGAAGCCTTTGCGTTCAAGAAATGGGGTGGAGAAGAAGGTTTAGATGAGGAATG      911
RAD       TGAAGTGGAAGCCTTTGCGTTCAAGAAATGGGGTGGAGAAGAAGGTTTAGATGAGGAATG      956
          ************************************************************

31Rp      GCAACGTCGTGAAGAAGGAAAGGCTCACAGAAGGGAGAAAAAATACGAAAAGAAAATCAA      1014
31Fp      GCAACGTCGTGAAGAAGGAAAGGCTCACAGAAGGGAGAAA-AATACGAAAAGAAAATCAA      970
RAD       GCAACGTCGTGAAGAAGGAAAGGCTCACAGAAGGGAGAAAAAATACGAAAAGAAAATCAA      1016
          **************************************** *******************

31Rp      GGAAATGCGACTGAAAACAAGAGCTCAAGAATATACTAATAGATTAAGAGAAAAGAAGCA      1074
31Fp      GGAAATGCGACTGAAAACAAGAGCTCAGGAATATACTAATAGATTA-GAAAAAAGAAGCA      1029
RAD       GGAAATGCGACTGAAAACAAGAGCTCAAGAATATACTAATAGATTAAGAGAAAAGAAGCA      1076
          ***************************.****************** **.**********

31Rp      TGGGAAAGCCCATATTCATCATTTTAGTGATCCAGTTGATGGAGGTATTGATGAAGACGG      1134
31Fp      TGGGAAAGCCCATATTCATCATTTTAGTGATCCAGTTGATGGAGGTATTGATGAAAACGG      1089
RAD       TGGGAAAGCCCATATTCATCATTTTAGTGATCCAGTTGATGGAGGTATTGATGAAGACGG      1136
          *******************************************************.****


31Rp      TTATCAAATTCAAAGAAA------------------------------------------      1152
31Fp      TTATCAATTCA-A-GAAGAAAGATGTAAGACGGCGGGCTAAA-ACTGAAAAATTTGAATT      1146
RAD       TTATCAAATTCAAAGAAGAAAGATGTAAGACGGCGGGCTAGAAACTGAAGAAATTGAATT      1196
          *******:* . * ***.

31Rp      ----   1152
31Fp      TAAA   1150
RAD       TTAA   1200
```

SOD1

```
31FP      ----------------------------------------------------TTTGTCAG      8
SOD1      ----ATGGTTCAAGCAGTCGCAGTGTTAAAGGGTGATGCCGGTGTCTCTGGTGTTGTCAA      56
31RP      TTTTTGGTTCAAAGCAGTCGCAGTGTTAAAGGGTGATGCCGGTGTCTCTGGTGTTGTCAA      60
                                                               ******

31FP      GTTCGAACAGGCTTCCGAATCCGAGCCAACCACTGTCTCTTACGAGATCGCTGGTAACAG      68
SOD1      GTTCGAACAGGCTTCCGAATCCGAGCCAACCACTGTCTCTTACGAGATCGCTGGTAACAG      116
31RP      GTTCGAACAGGCTTCCGAATCCGAGCCAACCACTGTCTCTTACGAGATCGCTGGTAACAG      120
          ************************************************************

31FP      TCCTAACGCAAAACGTGGGTTCCACATTCATGATTTTGGAAATGCCACCAAGGGTTGTGT      128
SOD1      TCCTAACGCAGAACGTGGGTTCCACATTCATGAGTTTGGAGATGCCACCAATGGTTGTGT      176
31RP      TCCTAACGCAGAACGTGGGTTCCACATTCATGAGTTTGGAGATGCCACCAATGGTTGTGT      180
```

```
               ********** ********************* ****** ********** ********

31FP           CTCTGCTGGTCCTCACTTCAATCCTTTCAAAAAAACACATGGTGCTCCAACTGACAAAGT      188
SOD1           CTCTGCTGGTCCTCACTTCAATCCTTTCAAGAAGACACATGGTGCTCCAACTGACGAAGT      236
31RP           CTCTGCTGGTCCTCACTTCAATCCTTTCAAGAAGACACATGGTGCTCCAACTGACGAAGT      240
               ****************************** ** ********************* ****

31FP           CAAACATGTCGGTGACATGGGTAACGTAAAGACGGACAAAAATGGTGTGGCCAAGGGCTC      248
SOD1           CAGACATGTCGGTGACATGGGTAACGTAAAGACGGACGAAAATGGTGTGGCCAAGGGCTC      296
31RP           CAGACATGTCGGTGACATGGGTAACGTAAAGACGGACGAAAATGGTGTGGCCAAGGGCTC      300
               ** ********************************** **********************

31FP           CTTCAAGGACTCTTTGATCAAGCTTATCGGTCCTACCTCCGTTGTAGGCAGAACCGTCTT      308
SOD1           CTTCAAGGACTCTTTGATCAAGCTTATCGGTCCTACCTCCGTTGTAGGCAGAAGCGTCGT      356
31RP           CTTCAAGGACTCTTTGATCAAGCTTATCGGTCCTACCTCCGTTGTAGGCAGAAGCGTCGT      360
               ***************************************************** **** *

31FP           TATCCACGCCGGCCAAAATGACTTAGGTAAGGGTGACACTGAAGAATCTTTGAAGACTGG      368
SOD1           TATCCACGCCGGCCAAGATGACTTAGGTAAGGGTGACACTGAAGAATCTTTGAAGACTGG      416
31RP           TATCCACGCCGGCCAAGATGACTTAGGTAAGGGTGACACTGAAGAATCTTTGAAGAACTG      420
               **************** ***************************************   *

31FP           TAATGCCGGTCCAAGACCACCCTGTGGTGTCATTGTTCTAACCAACTAAAAAAAGAA 424
SOD1           TAATGCCGGTCCAAGACCAGCCTGTGGTGTCATTGGTCTAACCAACTAA-------- 465
31RP           GTAAGT--------------------------------------------------- 426
                 * *
```
