# Supplementary material for: Saccharomyces cerevisiae DJ-1 paralogs maintain genome integrity through glycation repair of nucleic acids and proteins
Source: eLife. 2023 Aug 7;12:e88875. doi: 10.7554/eLife.88875 (PMC10431920; doi:10.7554/eLife.88875)
Supplement: Supplementary file 1. [file elife-88875-supp1.docx]

**TABLES**

**Supplementary file 1a.** List of yeast strains used in this study.

| Strain | Genotype | Source |
| --- | --- | --- |
| BY4741 WT | *MATa his3Δ1 leu2Δ0 met15Δ0 ura3Δ0* | Open Biosystems |
| Δ*hsp31* | *MATa his3Δ1 leu2Δ0 met15Δ0 ura3Δ0* Δ*hsp31::KanMX4* | (Bankapalli et al., 2015) |
| Δ*hsp32* | *MATa his3Δ1 leu2Δ0 met15Δ0 ura3Δ0* Δ*hsp32::hphNT1* | (Bankapalli et al., 2015) |
| Δ*hsp33* | *MATa his3Δ1 leu2Δ0 met15Δ0 ura3Δ0* Δ*hsp33::hphNT1* | (Bankapalli et al., 2015) |
| Δ*hsp34* | *MATa his3Δ1 leu2Δ0 met15Δ0 ura3Δ0* Δ*hsp34::URA3* | (Bankapalli et al., 2015) |
| Δ*31*Δ*32* | *MATa his3Δ1 leu2Δ0 met15Δ0 ura3Δ0* Δ*hsp31::KanMX4;* Δ*hsp32::hphNT1* | (Bankapalli et al., 2020) |
| Δ*31*Δ*33* | *MATa his3Δ1 leu2Δ0 met15Δ0 ura3Δ0* Δ*hsp31::KanMX4;* Δ*hsp33::hphNT1* | (Bankapalli et al., 2020) |
| Δ*31*Δ*34* | *MATa his3Δ1 leu2Δ0 met15Δ0 ura3Δ0* Δ*hsp31::KanMX4;* Δ*hsp34::URA3* | (Bankapalli et al., 2015) |
| Δ*31*Δ*32*Δ*33* (Δ*T*) | *MATa his3Δ1 leu2Δ0 met15Δ0 ura3Δ0* Δ*hsp31::KanMX4;* Δ*hsp32::hphNT1*;  Δ*hsp33::HIS3* | This study |
| Δ*31*Δ*32*Δ*33*Δ*34* (Δ*Q*) | *MATa his3Δ1 leu2Δ0 met15Δ0 ura3Δ0* Δ*hsp31::KanMX4;* Δ*hsp32::hphNT1*;  Δ*hsp33::HIS3*; Δ*hsp34::URA3* | This study |
| Δ*glo1* | *MATa his3Δ1 leu2Δ0 met15Δ0 ura3Δ0* Δ*glo1::hphNT1* | This study |
| WT/Hsp31-GFP | *MATa his3Δ1 leu2Δ0 met15Δ0 ura3Δ0 Hsp31-GFP::hphNT1* | This study |
| WT/Hsp32-GFP | *MATa his3Δ1 leu2Δ0 met15Δ0 ura3Δ0 Hsp32-GFP::hphNT1* | This study |
| WT/Hsp33-GFP | *MATa his3Δ1 leu2Δ0 met15Δ0 ura3Δ0 Hsp33-GFP::hphNT1* | This study |
| WT/Hsp34-GFP | *MATa his3Δ1 leu2Δ0 met15Δ0 ura3Δ0 Hsp34-GFP::hphNT1* | This study |
| WT/Rnr3-HA | *MATa his3Δ1 leu2Δ0 met15Δ0 ura3Δ0 RNR3-HA::NAT* | This study |
| Δ*31*Δ*32*Δ*33*Δ*34* (Δ*Q*)/ Rnr3-HA | *MATa his3Δ1 leu2Δ0 met15Δ0 ura3Δ0* Δ*hsp31::KanMX4;* Δ*hsp32::hphNT1*;  Δ*hsp33::HIS3*; Δ*hsp34::URA3*; *RNR3-HA::NAT* | This study |
| WT/Hsp31-HA | *MATa his3Δ1 leu2Δ0 met15Δ0 ura3Δ0 HSP31-HA::NAT* | This study |
| WT/Hsp32-HA | *MATa his3Δ1 leu2Δ0 met15Δ0 ura3Δ0 HSP32-HA::NAT* | This study |
| WT/Hsp33-HA | *MATa his3Δ1 leu2Δ0 met15Δ0 ura3Δ0 HSP33-HA::NAT* | This study |
| WT/Hsp34-HA | *MATa his3Δ1 leu2Δ0 met15Δ0 ura3Δ0 HSP34-HA::NAT* | This study |
| WT/RAD52-GFP | *MATa his3Δ1 leu2Δ0 met15Δ0 ura3Δ0 RAD52-GFP::LEU2* | This study |
| Δ*31*Δ*32*Δ*33*Δ*34* (Δ*Q*)/ RAD52-GFP | *MATa his3Δ1 leu2Δ0 met15Δ0 ura3Δ0* Δ*hsp31::KanMX4;* Δ*hsp32::hphNT1*;  Δ*hsp33::HIS3*; Δ*hsp34::URA3*; *RAD52-GFP::LEU2* | This study |
| WT/Hsp31-HA | *MATa his3Δ1 leu2Δ0 met15Δ0 ura3Δ0 HSP31-HA::hphNT1* | (Bankapalli et al., 2015) |
| WT/Hsp31-GFP | *MATa his3Δ1 leu2Δ0 met15Δ0 ura3Δ0 HSP31-GFP::hphNT1* | (Bankapalli et al., 2015) |

**Supplementary file 1b.** List of primers used in this study.

| Primer  Name | 5’→3' Sequence | Comments |
| --- | --- | --- |
| P1 | CTAGAACACTTTTCTCCTTCATTCAAAAAGAAAACTGGCCTTGCACGTACGCTGCAGGTCGAC | Forward, excision of Hsp32 or Hsp33 |
| P2 | CAAGCCAAAAAAAAGAAAAAAAAAGGAAAAAAAAGAAAACACAGCATCGATGAATTCGAGCTCG | Reverse, excision of Hsp32 or Hsp33 |
| P3 | TTTTTGACACTTCAGACCAGATACGCCACCAGCTACAAACTAACACGTACGCTGCAGGTCGAC | Forward, excision of Glo1 |
| P4 | CATTATTGTAAAAAAAAAATACTAACCTCCTATTCATTTCAACTTTATCGATGAATTCGAGCTCG | Reverse, excision of Glo1 |
| P5 | GAAACAAAAGACAAGGGAATTGTTG | Forward, excision confirmation of Hsp32 or Hsp33 |
| P6 | GGAGGGTATTCTGGGCCTCC | Reverse, excision confirmation of Hsp32 or Hsp33 |
| P7 | CGTGTACGCATGTAACATTATAC | Forward, excision confirmation of Glo1 |
| P8 | CCATCAAATTAATTTACTTAC | Reverse, excision confirmation of Glo1 |
| P9 | GTACTGCTCCTACTCCAGAAGCTTGTGAGTCATGTTCCGGTCGTACGCTGCAGGTCGAC | Forward, Rnr3-HA tagging |
| P10 | CCAAGTTAGATAAGGAAAGGGAAAAATGCCACCAGAAAGAAATCGATGAATTCGAGCTCG | Reverse, Rnr3-HA tagging |
| P11 | GTCTAATACTGGTTGAATTGTGCC | Reverse, tagging confirmation of Rnr3 |
| P12 | CCTATTCGACCACAATTAGAGCTATAAACGCATTATATAGCCGTACGCTGCAGGTCGAC | Forward, Hsp32/33/34-HA and GFP tagging |
| P13 | AAGCCTCCCCTTCAACAATTCCCTTGTCTTTTGTTTCAATCGATGAATTCGAGCTCG | Reverse, Hsp32/33/34-HA and GFP tagging |
| P14 | AAGATATTGGTTGGCAGAAATGTTTAATAAAAC | Reverse, tagging confirmation of Hsp32/33 |
| P15 | GAAGACCAAAGATCAATCCCCTGCATGCACGCAAGCCTACTGATATCGAATTCCTGCAGCCCGG | Forward, RAD52 GFP tagging |
| P16 | TAATGATGCAAATTTTTTATTTGTTTCGGCCAGGAAGCGTTGGAATATGTTCATAGGGTAGACGAAAC | Reverse, RAD52 GFP tagging |
| P17 | GGCCTTACCTTCTTCAGGC | Forward, tagging confirmation of RAD52 |
| P18 | CCTAAGGATTCCGCTGAAAAC | Reverse, tagging confirmation of RAD52 |
| P19 | ATGGTTCAAGCAGTCGCAGTGTTAAAGGG | Forward, amplification of yeast SOD1 |
| P20 | TTAGTTGGTTAGACCAATGACACCACAGGC | Reverse, amplification of yeast SOD1 |
| P21 | ATGACTCCCGAACAAAAGGCC | Forward, amplification of RAD14 |
| P22 | TTAAATGTCAATTTCTTCAGTTTCTAGC | Reverse, amplification of RAD14 |
| P23 | ATGTTAGATTTATTAAGATTACAATTAACAAC | Forward, amplification of COX2 |
| P24 | TTATTGTTCATTTAATCATTCCAAAAATTTAG | Reverse, amplification of COX2 |

**Supplementary file 1c.** List of plasmids used in this study.

| Plasmid Name | Comments |
| --- | --- |
| pRS 415_TEF_-Hsp31 | For complementation analysis |
| pRS 415_GPD_-Hsp32 | For complementation analysis |
| pRS 415_GPD_-Hsp33 | For complementation analysis |
| pRS 415_GPD_-Hsp34 | For complementation analysis |
| pRSF-Duet Hsp31 | For protein purification |
| pRSF-Duet Hsp32 | For protein purification |
| pRSF-Duet Hsp33 | For protein purification |
| pRSF-Duet Hsp34 | For protein purification |
| pRS 415_TEF_-MTS-mCherry | To visualize mitochondria |

**Supplementary reference**

Bankapalli, K., Saladi, S., Awadia, S. S., Goswami, A. V., Samaddar, M., & D'Silva, P. (2015). Robust glyoxalase activity of Hsp31, a ThiJ/DJ-1/PfpI family member protein, is critical for oxidative stress resistance in Saccharomyces cerevisiae. *J Biol Chem, 290*(44), 26491-26507. doi:10.1074/jbc.M115.673624

Bankapalli, K., Vishwanathan, V., Susarla, G., Sunayana, N., Saladi, S., Peethambaram, D., & D'Silva, P. (2020). Redox-dependent regulation of mitochondrial dynamics by DJ-1 paralogs in Saccharomyces cerevisiae. *Redox Biol, 32*, 101451. doi:10.1016/j.redox.2020.101451
